# Mechanical interactions dynamically influence rosette morphogenesis in the migrating zebrafish posterior lateral line primordium

**DOI:** 10.1242/dev.204973

**Published:** 2026-07-15

**Authors:** Abhishek Mukherjee, Michael Hilzendeger, Arin Rinvelt, Sana Fatma, Megan Schupp, Damian Dalle Nogare, Ajay B. Chitnis

**Affiliations:** Section on Neural Developmental Dynamics, Division of Developmental Biology, Eunice Kennedy Shriver National Institute of Child Health and Human Development, Bethesda, MD 20892, USA

**Keywords:** Agent-based models, NetLogo, CompuCell3D, Self-organization, Rosettes, Collective migration

## Abstract

A pre-pattern of Fibroblast growth factor signaling triggers formation of epithelial rosettes as protoneuromasts form periodically in the migrating posterior lateral line primordium. However, the number and size of epithelial rosettes is influenced by the balance of mechanical interactions that promote or oppose their formation. Selective slowing of leading cells in the primordium can result in the fusion of two rosettes to form one larger one, while slowing of trailing cells can result in splitting of a previously formed rosette to form two smaller ones. These observations can be accounted for by mechanics-based models, whereby local interactions associated with apical constriction and cell adhesion promote the formation of rosettes, while tension along the length of the primordium, influenced by the relative efficacy of leading and trailing cell migration, opposes their formation. We describe computational models that illustrate how the relative speed of leading versus trailing cells, as well as changes in cell adhesion and mechanical coupling, can influence the pattern of protoneuromast formation and deposition by the migrating primordium. Our studies illustrate how signaling and mechanics together influence morphogenesis in the migrating primordium.

## Teaser

**Summary:** Local interactions that promote epithelial rosette formation are opposed by tension associated with migration, which inhibits their formation, influencing the number and size of nascent neuromasts in the migrating primordium.

## INTRODUCTION

An embryo represents a myriad of self-organizing processes resulting from interactions at multiple scales to determine robust emergence of fate, form and function in the growing animal. Conceptual frameworks developed to account for development often emphasize interactions operating at specific scales or via specific cellular, molecular, genetic and physical mechanisms. The relative simplicity and accessibility of the zebrafish posterior lateral line system for live imaging and for cellular, molecular and genetic manipulation has led to its emergence as an attractive system in which to study development in a more integrative manner (Ghysen and Dambly-Chaudiere, 2007).

The zebrafish posterior lateral line primordium (PLLp) is a group of about 140 cells (Nogare et al., 2017) that migrates under the skin, over the horizontal myoseptum, from near the ear to the tip of the tail, periodically forming and depositing sensory organs called neuromasts. These neuromasts spearhead formation of the posterior lateral line, a sensory system that evolved to allow fish and amphibians to detect the pattern of water flow over their body surface (Ghysen and Dambly-Chaudiere, 2007). Each neuromast has sensory hair cells at its center, which are surrounded by support and mantle cells that cluster to form an epithelial rosette. Nascent neuromasts, or ‘protoneuromasts’, are periodically formed within the migrating primordium as interactions between primordium cells determine the specification of a central sensory hair cell progenitor. Each central sensory hair cell progenitor is surrounded by epithelialized cells that apically constrict and are recruited to form epithelial rosettes. Formation of protoneuromasts starts at the trailing end of the migrating primordium; as they mature, new protoneuromasts are sequentially formed progressively closer to the leading end of the primordium. Eventually, trailing cells lose their capacity for migration and they are deposited as neuromasts if they were successfully incorporated into protoneuromasts, or as inter-neuromast cells between the periodically deposited neuromasts, if not.

The sequential formation of protoneuromasts and collective migration of the primordium along the horizontal myoseptum is coordinated by polarized Wnt and Fibroblast growth factor (FGF) signaling in the primordium (Aman and Piotrowski, 2008). Wnt activity dominates at the leading end, while it is relatively low at the trailing end. In the context of this polarized activity, Wnt signaling promotes expression of Fgf3 and Fgf10. However, Wnt active cells also express factors that inhibit them from responding to the FGF ligands they produce (Aman and Piotrowski, 2008; Matsuda et al., 2013). Instead, these FGFs initiate FGF signaling at a distance at the trailing end, where there is less inhibition of FGF signaling by Wnt activity. FGF signaling in this trailing domain determines expression of the diffusible Wnt inhibitor Dkk1b, where, together with other factors, it inhibits Wnt activity. By inhibiting FGF activity and by promoting expression of factors that promote Wnt activity, Wnt active cells indirectly and directly promote Wnt activity. The local activation of Wnt signaling, coupled with its long-range inhibition by FGF signaling, constitutes a patterning system that has the potential to initiate FGF signaling in a center-biased pattern (Dalle Nogare and Chitnis, 2020). This center-biased FGF signaling center initiates formation of a nascent protoneuromast by coordinating both the specification of a central *atoh1a*-expressing sensory hair cell progenitor and reorganization of its surrounding cells to form an epithelial rosette (Lecaudey et al., 2008; Nechiporuk and Raible, 2008). While Wnt activity inhibits FGF signaling and morphogenesis of protoneuromasts, FGF signaling promotes epithelialization, expression of factors such as *shroom3* that promote apical constriction (Ernst et al., 2012), and reorganization of cells to form epithelial rosettes. Center-biased FGF signaling also determines specification of a central *atoh1a*-expressing hair cell progenitor, which subsequently contributes to maturation of the protoneuromast by stabilizing the reorganization of surrounding cells as epithelial rosettes (Kozlovskaja-Gumbriene et al., 2017; Lecaudey et al., 2008; Matsuda and Chitnis, 2010). Maturation of the trailing protoneuromast also contributes to inhibition of Wnt signaling in the trailing zone. This restricts Wnt activity to a smaller leading zone, establishing the conditions for the emergence of another center-biased FGF signaling domain in the wake of the shrinking Wnt system, and the formation of a new protoneuromast. With each cycle, new protoneuromasts form progressively closer to the leading end.

FGFs secreted by leading Wnt-active cells also serve as directional migratory cues for trailing cells (Dalle Nogare et al., 2014). As FGF-responsive trailing cells migrate toward the leading cells, the caudal migration of the primordium follows a path defined by the expression of the chemokine Cxcl12a (previously known as Sdf1a) (Haas and Gilmour, 2006; Li et al., 2004; Sapede et al., 2005), guided by a self-generated local gradient of chemokine activity. Wnt activity in the primordium determines polarized expression of two chemokine receptors, chemokine (C-X-C motif) receptor 4b (Cxcr4b) and atypical chemokine receptor 3b (Ackr3b, previously Cxcr7b) (Dambly-Chaudiere et al., 2007; Haas and Gilmour, 2006; Valentin et al., 2007). Wnt activity promotes *cxcr4b* expression in a leading zone, while it inhibits expression of *ackr3b*, resulting in the restriction of its expression to a trailing zone, where Wnt signaling is low (Aman and Piotrowski, 2008). Ackr3b-expressing cells in the trailing zone internalize Cxcl12a and degrade it, leading to formation of a local Cxcl12a gradient (Bussmann and Raz, 2015; Dona et al., 2013; Venkiteswaran et al., 2013). Cxcr4b-expressing cells in the leading zone respond to this self-generated gradient to determine directed migration of the primordium. In this manner, chemokine and FGF signaling in leading and trailing cells, respectively, play complementary roles in coordinating directed migration of the primordium.

One consequence of leading and trailing cells migrating in response to different mechanisms is that mechanical tension along the length of the primordium, associated with collective migration, is expected to be influenced by the relative efficiency of migration determined by these two signaling systems. Although a pre-pattern of FGF signaling seeds periodic formation of protoneuromasts, this study shows that morphogenesis of the epithelial rosettes is initially unstable, and that the number and size of the rosettes can be altered, at least transiently, by manipulations that potentially alter mechanical tension along the length of the primordium. Selective slowing of leading cells in the primordium can result in the fusion of two rosettes to form one larger one, while slowing of trailing cells can result in splitting of a previously formed rosette to form two smaller ones. We describe the development of two classes of computational models that illustrate how the local interactions associated with apical constriction and cell adhesion could promote clustering and formation of rosettes, while tension along the length of the primordium could serve as a long-range force that opposes such aggregation. In this manner, the models illustrate how a mechanical version of local activation coupled with long-range inhibition could influence the pattern of cell clustering along the length of the primordium. We suggest that while FGF signaling creates a pre-pattern that coordinates the formation of protoneuromasts with a sensory hair cell progenitor at the center of each epithelial rosette, mechanical interactions within the primordium have the potential to determine self-organization of periodic cell clusters independent of this pre-pattern, i.e. signaling and mechanics collectively influence the pattern of cell clustering in the migrating primordium.

## RESULTS

### Selective slowing of the leading PLLp cells results in fusion of rosettes

**Fig. 1.**
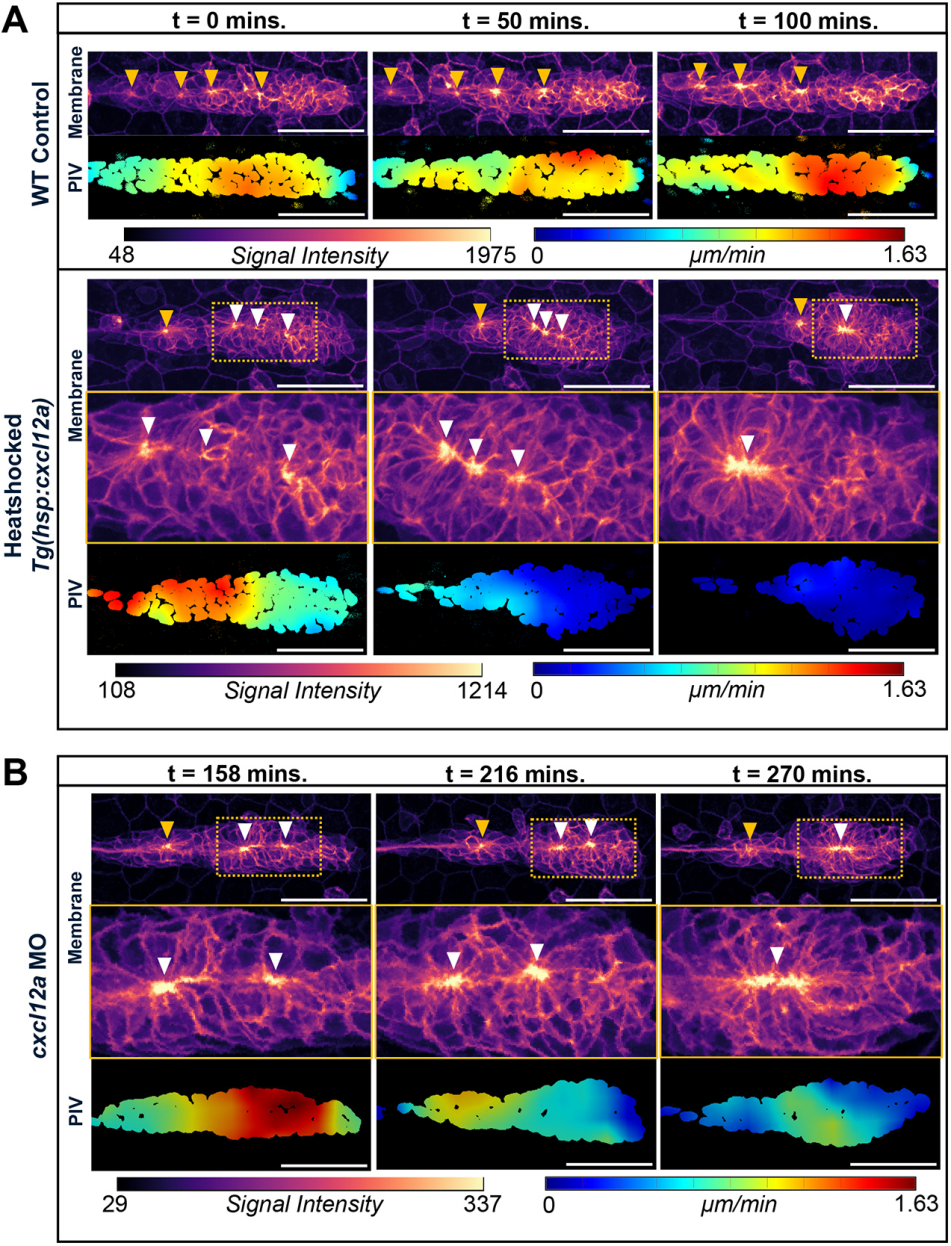
**Differential slowing of the leading cells in the PLLp results in fusion of rosettes.** (A) Fluorescent primordium membrane images in *Tg(cldnb:lyn-egfp)* embryos with corresponding particle image velocimetry (PIV) maps, overlaid on PLLp cell nuclei. A wild-type (WT) control PLLp (top panel) is compared to a heat-shocked *Tg(hsp:cxcl12a)* PLLp (bottom panel). Membrane images are shown with an intensity-based hue (warm=high signal intensity) to visualize apical constrictions. Warm hues in PIV maps indicate relatively high cell velocities (μm/min). Magnified images of the fusion event (orange dotted rectangles) are shown in the middle panel. Fusion of rosettes upon heat shock was observed in 10/10 PLLps. (B) A PLLp of a *cxcl12a* morpholino (MO)-injected embryo with membrane (top panel), magnified membrane (middle panel) and PIV maps (bottom panel) are shown across time. Fusion of rosettes was observed in 2/8 PLLps. Orange arrowheads indicate pre-existing constrictions that do not fuse over time. White arrowheads indicate constrictions that fuse. Corresponding color bars are shown below the respective panels. Note that deposited neuromasts are shifted out of the frame of view. Scale bars: 50 μm.

Chemokine-dependent migration can be disrupted by induction of exaggerated broad *cxcl12a* expression or by partially knocking down *cxcl12a*. To exaggerate *cxcl12a* expression, *Tg(cldnb:lyn-egfp); TgBAC(cxcr4b:h2a-mcherry); Tg(hsp:sdf1a)* triple transgenic embryos were heat-shocked between 27 and 28 hours post-fertilization at 37.5°C for 20 min. Broad induction of *cxcl12a* expression initially slows cells in the leading domain of the primordium before primordium migration eventually stalls (Fig. 1A; Movies 1 and 2) (Dalle Nogare et al., 2014). After stalling for approximately 7-8 h, migration eventually resumes in the caudal direction (Movie 2) (Lau et al., 2020; Wong et al., 2020).

Analysis of cell movement with particle image velocimetry (PIV) showed that, following heat shock-induced overexpression of *cxcl12a*, leading cells initially moved slower than trailing cells (Fig. 1A, bottom panel; Movies 3 and 4). In 10/10 imaged primordia, the relative slowing of leading cells coupled with continued movement of trailing cells was accompanied by some apical constrictions moving closer to each other and eventually fusing to form a single larger rosette (white arrowheads in Fig. 1A, bottom panels). Rosettes did not fuse in the wild-type control shown (Fig. 1A, top panels), where the leading domain predominantly migrated at relatively higher speeds than the trailing domain.

**Fig. 2.**
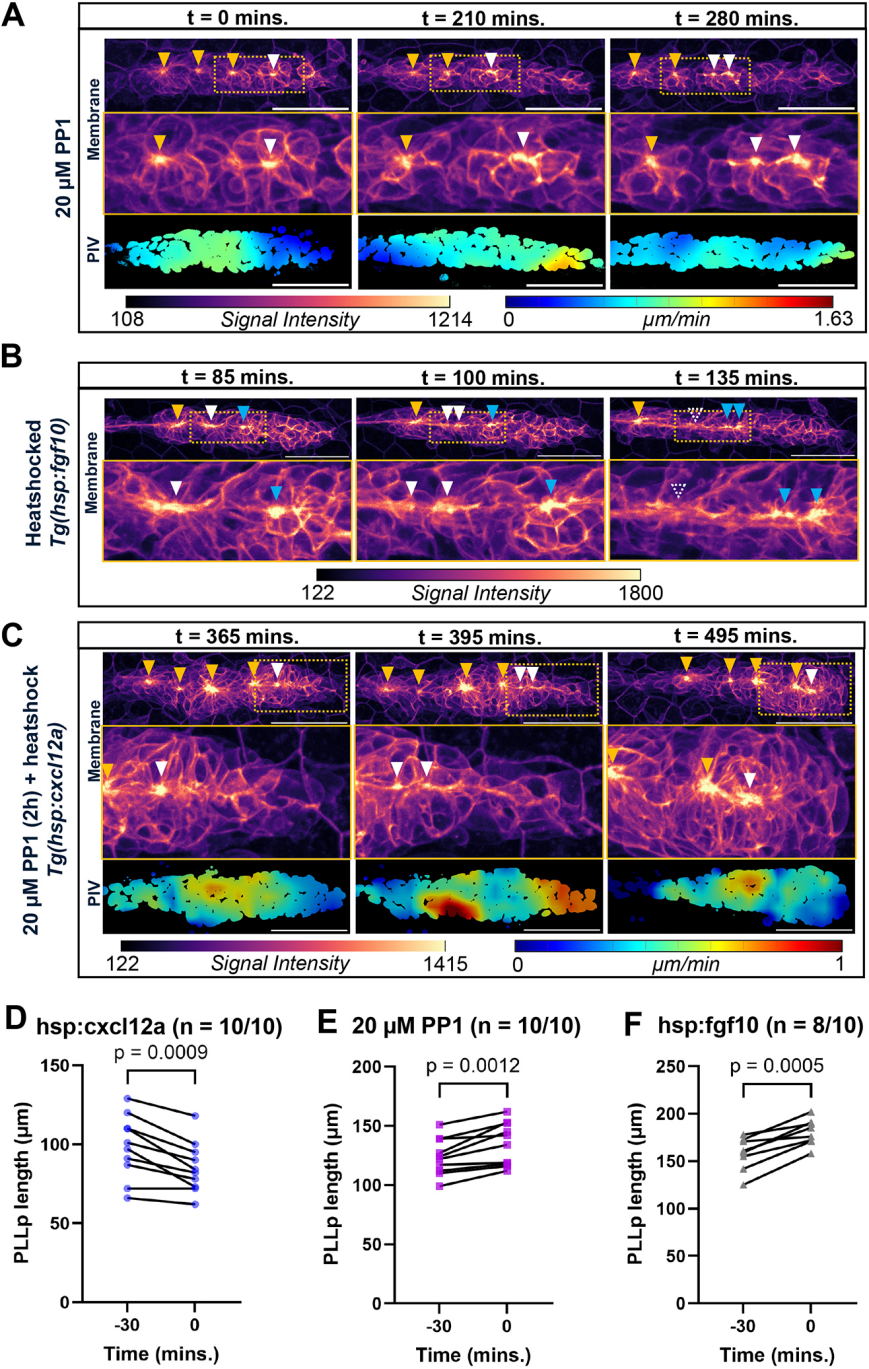
**Selective slowing of the trailing domain results in splitting of rosettes.** (A) Membrane images (top panel) of 20 μM PP1 drug-treated PLLp with their corresponding PIV maps (bottom panel) across time. Splitting of rosettes was observed in 10/10 PLLps. Orange arrowheads indicate constrictions that do not split. White arrowheads indicate split constrictions. (B) A *Tg(hsp:fgf10)* PLLp heat-shocked for *fgf10* overexpression is shown. Splitting of rosettes was observed in 8/10 PLLps. White arrowheads indicate the first constriction that splits (middle panel) and then dissolves (dotted arrowhead, right panel), while cyan arrowheads denote the second instance of constriction splitting (right panel). This panel is reproduced in Fig. S1A, with a graph of the distances measured shown in Fig. S1B. (C) A PLLp treated with 20 μM PP1 for 2 h before being heat-shocked to overexpress *cxcl12a* depicts splitting and subsequent fusion of a constriction (white arrowheads). Splitting and then re-fusing of rosettes was observed in 5/16 PLLps, while 11/16 PLLps demonstrated only splitting of existing rosettes. Membrane images (top and middle panels) and PIV maps (bottom panel) are shown. Corresponding color bars are shown. Scale bars: 50 μm. (A-C) Warm hues in PIV maps and membrane images indicate relatively high cell velocities (μm/min) and high signal intensity, respectively. Magnified membrane images are of dotted rectangular regions. (D-F) Change in PLLp length over 30 min time duration, with 0 denoting the time instant when rosette fusion or split occurs, quantified upon overexpression of *cxcl12a* (D), 20 μM PP1 treatment (E) and overexpression of *fgf10* (F). Only those PLLps where rosettes were observed to either fuse or split were included in this analysis. All datasets (three conditions and two per condition) were found to pass the Shapiro–Wilk Normality test. Statistical significance for each condition was calculated between −30 and 0 using two-tailed, paired *t*-tests.

Partial knockdown of *cxcl12a* with injection of 2.0 ng of *cxcl12a* morpholino into zebrafish embryos at the one-cell stage intermittently slowed or stalled migration of the primordium (Fig. 1B; Movies 5 and 6), potentially because it created patches of low or absent *cxcl12a*. Although this manipulation had an opposite effect on *cxcl12a* levels, effects on epithelial rosette formation were like those observed with overexpression. That is, the leading cells stalled or slowed down briefly leading to fusion of some apical constrictions. As effects of partial knockdown of *cxcl12a* were variable, this phenomenon was only observed in 2/8 primordia that were imaged. Similar effects suggested that reorganization of apical constrictions does not correlate with changes in the level of chemokine signaling, but, rather, to their common effect of selectively slowing migration of leading cells in the primordium, while trailing cells continue to move, resulting in concertina-like compression of the migrating primordium (Fig. 2D).

### Selective slowing of the trailing PLLp cells results in splitting of rosettes

PP1, a Src kinase inhibitor, has previously been used to inhibit collective migration of the primordium (Neelathi et al., 2018). PIV analysis showed that, as primordium migration slows in response to 20 μM PP1, trailing cells begin to slow earlier than leading cells (Fig. 2A, t=210 min), before the entire primordium eventually slows down (Fig. 2A, t=280 min; Movie 7). In this context, selective slowing of trailing cells caused the primordium to stretch (Fig. 2E), and this was accompanied, in the example shown, by splitting of a leading apical constriction to form two constrictions (Fig. 2A, t=280 min). The splitting of constrictions was observed in 10/10 primordia imaged following PP1 exposure.

Heat shock-induced Cxcl12a overexpression disrupted leading cell migration, so we hypothesized that FGF overexpression, which guides trailing cells, would similarly impair their migration. Consistent with this prediction, heat shock-induced expression of Fgf10 using *Tg(hsp:fgf10); Tg(cldnb:lyn-egfp)* embryos led to transient slowing of the trailing domain and subsequent stretching of the PLLp (Fig. 2F; Fig. S1B). This was associated with splitting of constrictions (t=100 and 135 min; Fig. 2B, white and blue arrowheads), and, in the example shown, eventual loss of a constriction (Fig. 2B, white dashed arrowhead; Fig. S1A; Movie 8). The splitting of constrictions was observed in 8/10 primordia imaged following heat shock-induced Fgf10 expression. Furthermore, a combination of manipulations could be used to reverse the splitting of rosettes, resulting from slowing of trailing cells with 20 μM PP1 for 2 h, in 5/16 primordia by additionally slowing leading cells with heat shocked-induced *cxcl12a* expression (Fig. 2C, white arrowheads; Movie 9).

Apical constriction fusion or splitting events were most obvious in experimentally manipulated embryos in association with slowing and speeding of the leading domain. However, such fusion and splitting events were also sometimes observed in control embryos (Fig. S1C,D) in association with periodic slowing and speeding of the primordium. Together, our results demonstrate that rosettes split when selective slowing of trailing cells stretches the PLLp, while differential slowing of leading cells results in compaction that is associated with rosette fusion (Fig. 2D-F). Splitting or fusion of constrictions was typically observed in the less-mature protoneuromasts toward the leading end. It was observed in relatively mature trailing protoneuromasts in only 3/44 primordia, suggesting greater instability in less mature protoneuromasts.

### Models to account for dynamic reorganization of apical constrictions

The fusion or splitting of pre-existing apical constrictions, when leading or trailing cells preferentially slow down, suggested that formation of epithelial rosettes is influenced by the tension along the length of the primordium. When leading cells are slower than trailing cells, the primordium length decreases (Fig. 2D), and the tension along the length of the primordium, which opposes cell clustering, reduces. This allows pre-existing apical constrictions to come together to form fewer, larger rosettes. Conversely, when trailing cells selectively slow down and leading cells keep moving, the primordium lengthens (Fig. 2E,F), tension opposing cell clustering increases, and pre-existing rosettes are pulled apart forming more and smaller rosettes, or they disappear. Two sets of models were developed to visualize this emergent behavior, one an agent-based model (ABM) developed with NetLogo (Wilensky, 1999) and the other a Cellular Potts model (CPM) developed with CompuCell3D (Swat et al., 2012).

### An ABM of dynamic cell clustering in the primordium

**Fig. 3.**
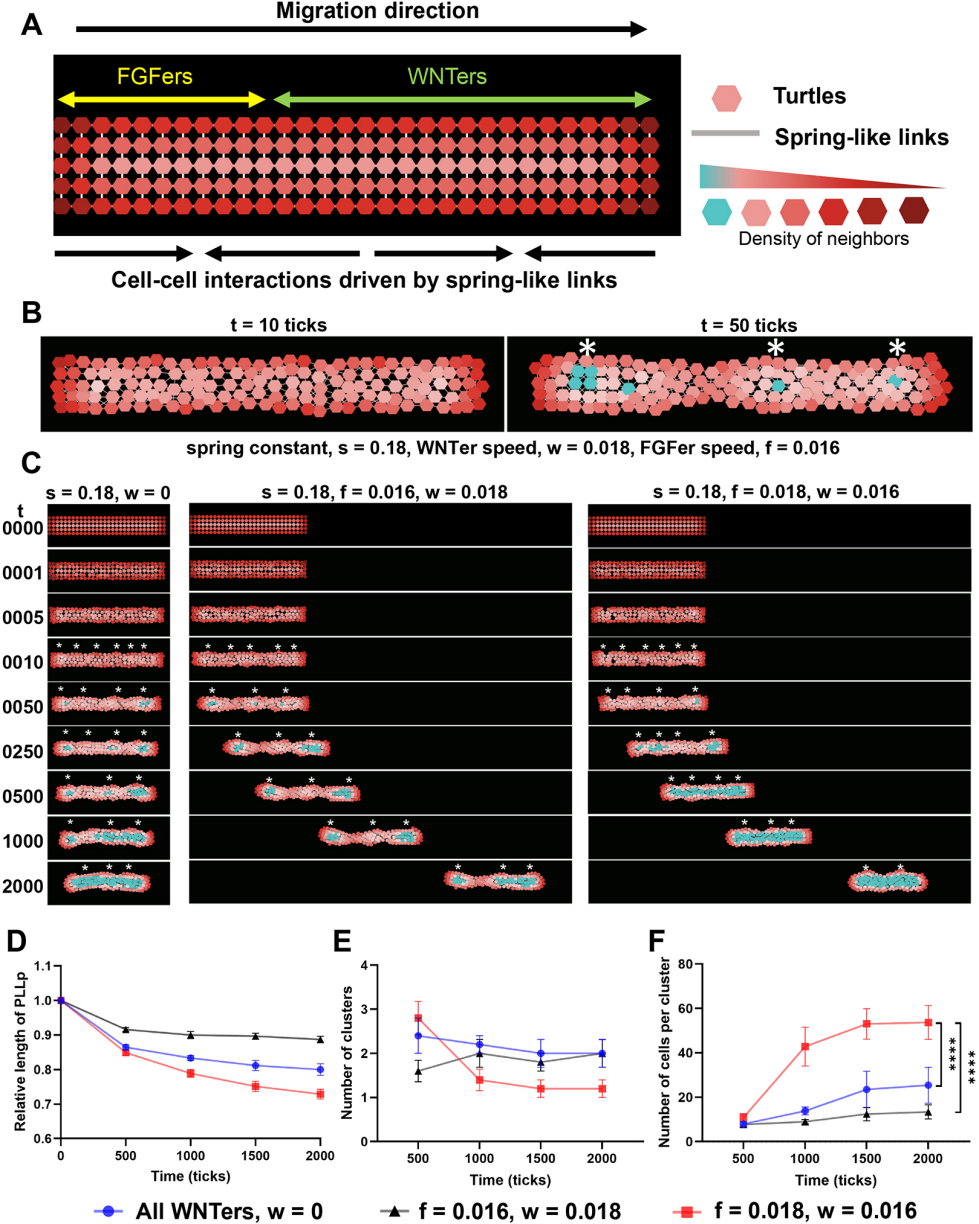
**An ABM of the PLLp illustrates how mechanical interactions can determine self-organization of cell clustering.** (A) Model primordium with leading WNTers and trailing FGFer turtles. Spring-like links mechanically couple turtles whose emergent density is represented by a color code. (B) Self-organized clustering in an example simulation with the following parameters: spring constant, s=0.18; WNTer speed, w=0.018; FGFer speed, f=0.016. Local clusters are visually identified as lateral bulges in the column and marked with white asterisks. (C) Sequential output of simulations without migration (left column; Wnt domain=100% of PLLp length, w=0), with WNTer speed (w)>FGFer speed (f) (middle column), and with WNTer speed (w)<FGFer speed (f) (right column). Local clusters with visible lateral bulges are marked with white asterisks. The left column is also shown in Fig. S2A. (D) Relative change in length of model PLLps over time compared to their original lengths at the start of the simulation. (E) Change in the number of clusters over time. (F) Number of cyan cells per cluster. ****P<0.0001. In D-F, the three simulation conditions are represented by blue (w=0), black (f=0.16, w=0.18) and red (f=0.18, w=0.16). Data were collated over five different simulations per condition. Means of five simulations per condition for time=500, 1000, 1500 and 2000 ticks are represented as datapoints while error bars signify s.d. Statistical significance was calculated by two-way ANOVA and Tukey's multiple comparisons tests.

**Fig. 4.**
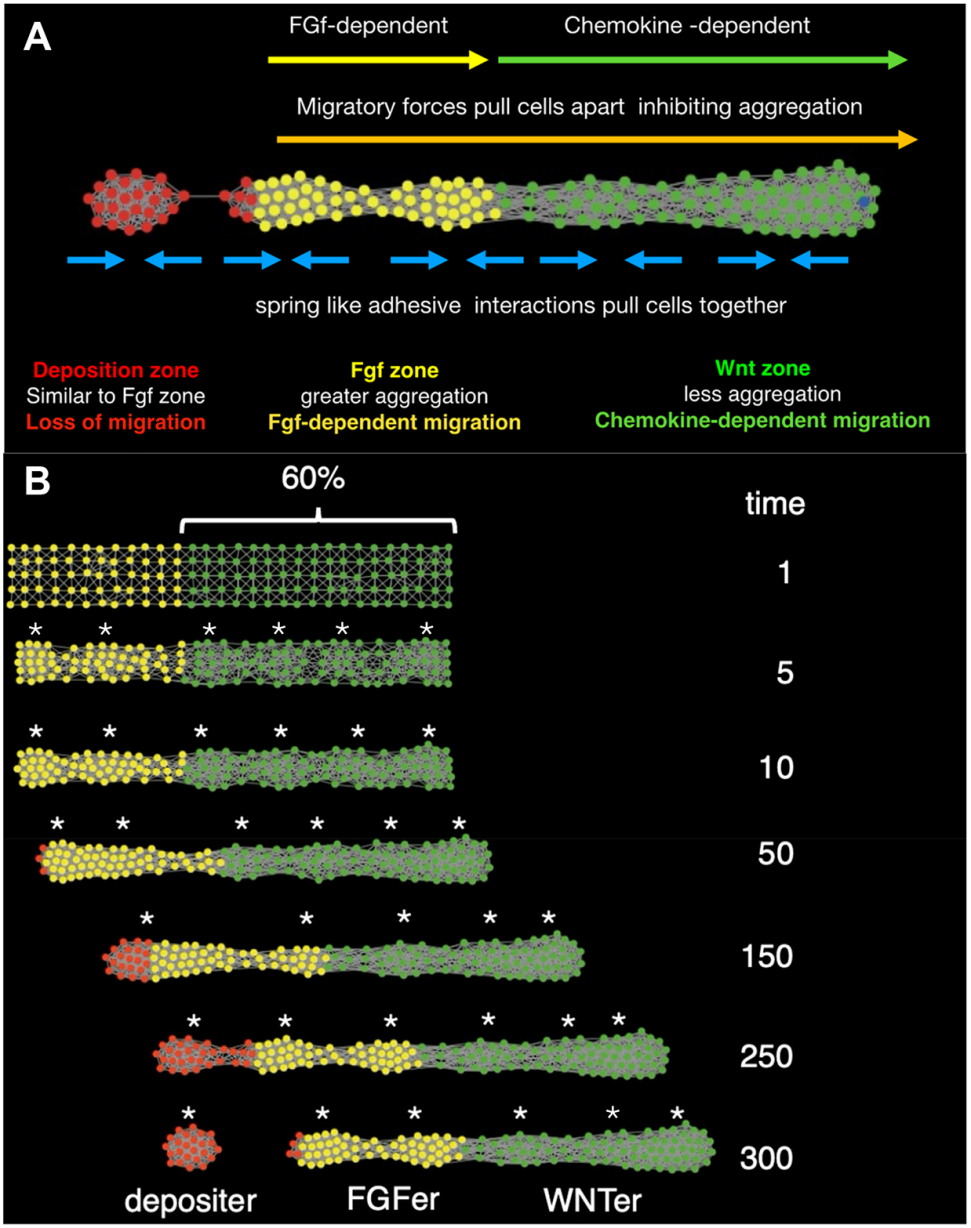
**Agent-based PLLp model with neuromast deposition incorporated.** (A) This ABM PLLp has three types of cells: WNTers (green), FGFers (yellow) and Depositers (red). The WNTer domain shrinks at a defined rate and the FGFer domain is dynamically shrunk so that WNTer domain is consistently 60% the length of the primordium (WNTer+FGFer domains). Turtles trailing the FGFer domain are designated as Depositers. At each iteration, each turtle makes links with other turtles within a two-patch radius. Details and parameters used in model illustrated are described in Materials and Methods. Arrows indicate forces associated with springs that pull cells together (blue) while tension associated with migration pulls them apart (orange). The net tension is determined by relative efficacy of ‘chemokine-dependent’ movement of WNTers and FGF-dependent movement of FGFers. (B) Timed progression of the model PLLp. Self-organized clusters are identified visually and denoted by white asterisks. Five different simulation runs were performed.

The primordium in the NetLogo ABM was represented as a column of 150 turtles, 5 turtles wide and 30 long, approximating the number of primordium cells *in vivo* (Figs 3 and 4) (Dalle Nogare and Chitnis, 2017a). ‘WNTer’ and ‘FGFer’ turtles represented cells in the domains dominated by Wnt and FGF activity, respectively. Based on observations *in vivo*, WNTers typically occupied the leading 60% of the model primordium (Fig. 3A).

The model was used to visualize how spring-like interactions between cells can determine self-organized clustering of cells and how varying the ‘spring-constant’ of links or the relative ‘speed’ of WNTers and FGFers influences the pattern of cell aggregation. Aggregation of cells was visualized in two ways; first, the shade of red used for each turtle reflected how many neighbors it had within a defined radius of three patches (a brighter shade for more, dark shades for fewer). Second, when the number of neighbors within the defined radius of a turtle exceeded a defined threshold (≥32 neighbors in Fig. 3B), its color was automatically set to cyan. In the simplest case, in which the model PLLp was composed of only WNTers, connected with links that had a spring-constant (s) of 0.18, and there was no migration (Fig. 3C, left panel; Movie 10, top panel), turtles along the entire primordium began to form local clusters (white asterisks). Initially, a larger number of smaller clusters formed, which progressively coalesced together to form fewer but larger aggregates as the length of the primordium progressively decreased (Fig. 3D-F, blue curves). The likelihood of turtles aggregating increased with increasing spring constant (Fig. S2). When the speed of the leading WNTers was higher than the trailing FGFers (Fig. 3C, middle panel; Movie 10, middle panel), the resulting tension along the length of the primordium stretched the primordium, creating three relatively stable clusters (Fig. 3D-F, black curves). However, as the WNTers in the leading 60% of the primordium move at the same speed, eventually the leading two clusters began to coalesce to form a larger dense cluster (compare t=1000 and t=2000 in Fig. 3C, middle panel). When WNTers had a slower speed than FGFers (WNTer speed, w=0.016; FGFer speed, f=0.018), coalescing of cells to form aggregates was accelerated and two dense clusters were formed by t=2000 (Fig. 3C, right panel; Movie 10, bottom panel). These results, taken together, suggest that, while contractile spring-like forces can promote aggregation of cells, tension caused by differential migration speeds can pull them apart and inhibit aggregation. Our results illustrate that even a simple model can demonstrate how cell clustering and morphogenesis can be modulated by the interplay of contractile and tensile forces.

During the migration of the primordium in the embryo, the Wnt system progressively shrinks, as new protoneuromasts form in its wake. However, as the Wnt system shrinks, the primordium shrinks as well, and its length scales with the length of the Wnt system (Dalle Nogare and Chitnis, 2017a). In the model, the Wnt domain could be set to shrink at a defined rate, and the length of the model primordium was adjusted so that the Wnt domain was consistently 60% the length of the primordium (Fig. 4). FGFers that extended in the trailing direction beyond the computed length of the primordium at any time were re-specified as a third breed of turtles called ‘Depositers’. In the version of the model illustrated in Fig. 4, the active migration speed of FGFers was set at 0 and their movement was entirely dependent on mechanical coupling with each other and with leading WNTers through links for which the break-threshold was set above ten to tolerate stretching of links without breaking to ensure their collective migration. WNTer speed was set at 0.05 patches/tick. Depositer parameters matched those of FGFers, they also had a migration speed of 0 and did not proliferate. In contrast to WNTers and FGFers, which had a relatively high break-threshold, Depositers made links with a low break-threshold, which was set at 1. So that as the rest of the turtles in the model primordium kept moving, Depositers broke their links with the FGFers and were ‘deposited’. In this manner, the model was used to visualize how cell clusters form and deposit in the wake of a progressively shrinking Wnt system (Fig. 4B; Movie 11). Setting active migration speed of FGFers at 0 increased stretch in the model PLLp and helped illustrate how strong mechanical coupling can determine collective migration, and how low mechanical coupling can regulate deposition. Details of the model shown in Fig. 4, and parameters used are provided in Materials and Methods and Table S2 (Netlogo code).

One important limitation of NetLogo ABMs is that each cell is represented as a point on a lattice with no real spatial dimension, so spring-like connections between turtles are used to simulate interactions that are likely to arise from both cell–cell adhesion and cell contractility. CPMs (Glazier–Graner–Hogeweg models) using the CompuCell3D modeling environment (Swat et al., 2012) were developed to model these distinct interactions more explicitly. In a CPM, each cell is represented as a set of pixels that define the position, size/shape, and degree of contact with other cells in the model. The first step in the development of the model is to define the different types of cells that will be interacting, their initial configuration, and features of the environment in which the cells will operate. Each different cell type has unique target parameters, including size (perimeter, surface area), degree of adhesivity to other cells of the same or different type, and movement in response to potential chemotactic influences on the cell, all factors contributing towards determining the energy of the system (Eqns 1 and 4; Materials and Methods). At each iteration of the program, pixels from the set representing each cell are randomly added, subtracted, or relocated within or adjacent to the cell's current pixel set. This results in growth, shrinkage, shape alteration, net movement of each cell, or variation in contact with adjacent cells. Whether this change in the cell pixel position is accepted or rejected at each iteration depends on an energy function that computes whether the resulting change moves the cell closer to or further from pre-defined target parameters (Eqn 2; Materials and Methods). Deviation of the new parameters for each cell from its pre-defined target parameters is associated with an energy cost, and the sum of these energies is used to determine whether moving the pixel is associated with a net reduction in the energy state of the cell; if the net energy is reduced, the change is accepted, if not it is either rejected, or accepted at a low probability (Eqn 3; Materials and Methods). Iteration of this process of randomly moving pixels associated with each cell, coupled with accepting or rejecting the change based on whether it lowers net energy of the system, allows it to evolve over time. In this way, the model simulates the potential trajectory of the system as cells dynamically change size, shape, position, and association with neighboring cells.

### A CPM recapitulates rosette formation and emergent behavior of the primordium

**Fig. 5.**
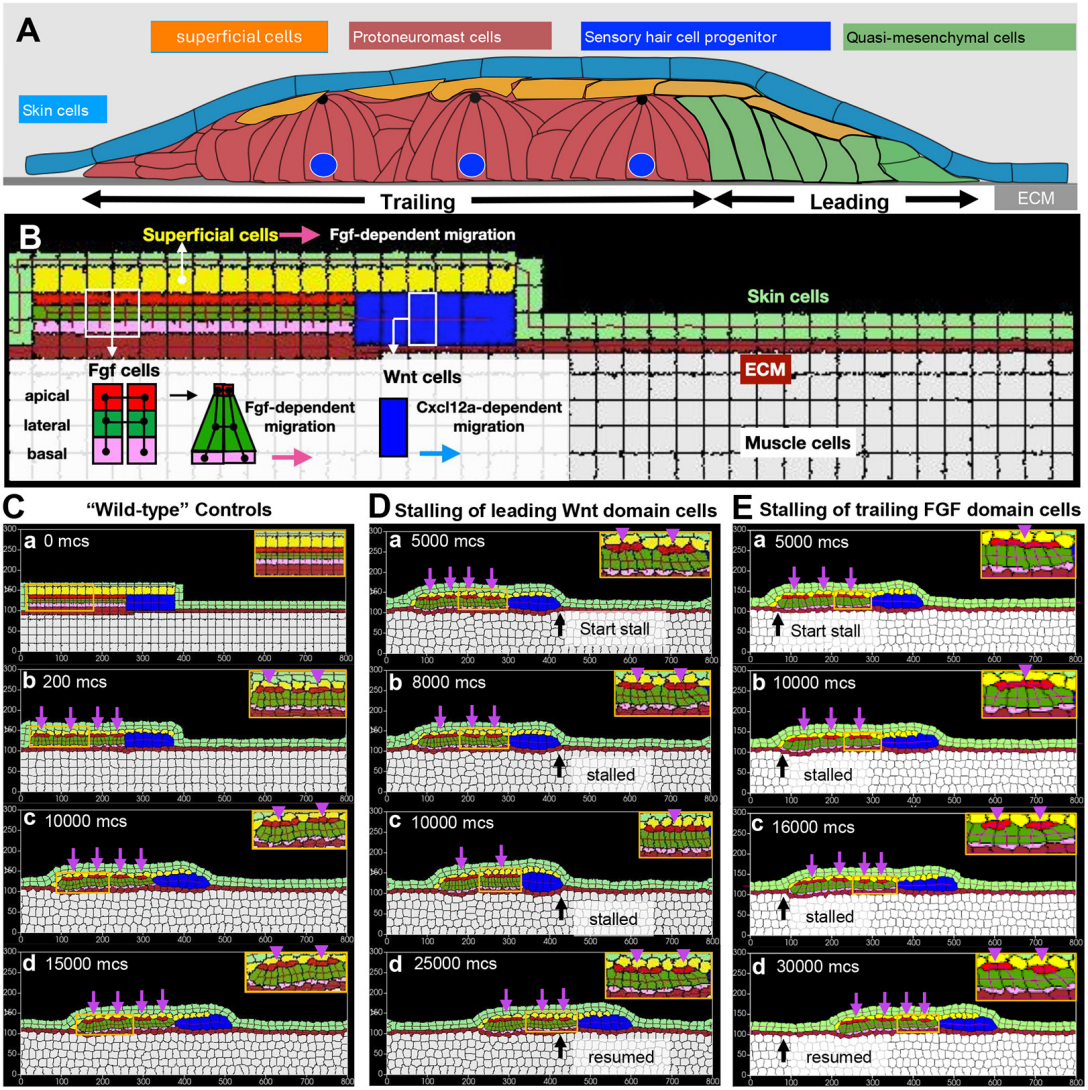
**CPM of PLLp recapitulates physical phenomena of rosettes fusing or splitting due to compression or tension along PLLp length.** (A) Schematic showing the broad categories of cells that compose a typical wild-type PLLp and the extracellular environment. (B) A CPM simulation setup, incorporating different types of cells that constitute the PLLp and the main components of their extracellular environment. A schematic (inset) is shown where apicobasally polarized cells in the trailing FGF domain are represented as composites of three compartments, apical (red), lateral (green) and basal (pink). Non-apicobasally polarized cells in the leading Wnt domain are shown in blue. FPP links are shown in black. Increasing the spring-constant of the FPP link between adjacent apical compartments simulates apical constriction (black arrow). Pink arrow represents a force that determines FGF-dependent migration of the basal compartment of FGF cells and superficial (sheath) cells, blue arrow represents force associated with Cxcl12a-dependent migration of Wnt cells. (C) Progression of migration and rosette formation of a model wild-type PLLp, showing the start of a simulation (a), and re-organization of rosettes (b-d). (D) Progression of a model PLLp simulating stalling of the leading unpolarized blue cells, depicting start of the stalling event (a), rosette fusion (b, c), and splitting of a fused rosette (d) (*n*=5/5 simulations). Black arrows denote the approximate location of the leading edge of the Wnt domain when stalling started. (E) Progression of a model PLLp simulating stalling of the trailing polarized FGF cells, depicting start of the stalling event (a), and progressive splitting of an existing rosette to form smaller rosettes (b-d) (*n*=4/5 simulations). Black arrows denote the approximate location of the trailing edge of the FGF domain when stalling started. In C-E, insets show magnified views of regions marked with solid rectangles. Magenta arrowheads mark rosettes in the insets, while magenta arrows mark those in the main figure panels. *x*-axis and *y*-axis represent the *x*- and *y*-dimensions (in pixels) of 2D space in which the model PLLp migrates.

The lateral line primordium migrates between the skin above and extracellular matrix (ECM) below with three types of cells that contribute to its organization (Dalle Nogare et al., 2020) (Fig. 5A). Corresponding to this schematic, the CPM model had six types of cells. The primordium was represented by WNT, FGF and superficial or sheath cells, which migrated in confinement between skin cells above, and the ECM and muscle cells below (Fig. 5B; see Materials and Methods section for details). Sensory hair cell progenitors were not represented in the model.

The quasi-mesenchymal cells without apical basal polarity in the leading Wnt active domain were represented by WNT cells that migrate in response to a force vector representing Cxcl12a signal-induced migration. The apicobasally polarized FGF cells in the trailing domain were represented as composites of three compartments, apical, basal and lateral, each represented by individual cell types, Fgfapi, Fgflat and Fgfbas, respectively, that were assigned target parameters and spring-like ‘FocalPointPlasticity’ (FPP) links representing their unique features.

Although direct information about the adhesive nature of each cell was not available, a number of assumptions were made based on the fact that there was little mixing of cells (Nogare et al., 2017), some cells or cell compartments are more or less likely to be in close apposition, and some cells or cell compartments were expected to have different levels of motility. While detailed parameters are provided in the Supplementary Materials and Methods section and Tables S3-S6, the following section summarizes how these features influenced parameters chosen for selective adhesion between different cell types of compartments.

Adhesive interactions between adjacent skin cells above and between ECM cells below had to be especially strong so that the primordium would not break through these barriers but migrate sandwiched between them. Additionally, FPP links were created and maintained between adjacent skin cells and adjacent ECM cells to preserve integrity of these layers. Adhesion between individual Wnt cells in the leading zone and FGF cells in the trailing zone had to be adequate to prevent cells within these domains from separating during migration. Furthermore, as there was a discrete boundary between the leading domain with Wnt cells and trailing domain with FGF cells, especially strong adhesion had to be maintained at the interface between Wnt and FGF cell domains to prevent them from separating during migration. Furthermore, FPP links were specified between all the Wnt cells, the Fgflat domains of FGF cells, and between the cells at the Wnt–FGF boundary to promote mechanical coupling. Adhesion between compartments of adjacent FGF cells was progressively weaker along their apical-basal axis; strongest adhesion between adjacent apical compartments (Fgfapi-Fgfapi) facilitated spontaneous organization of individual cell clusters or rosettes along the domain of FGF cells, adhesion between lateral compartments (Fgflat-Fgflat) was strong enough to maintain cohesive migration of FGF cells, while weakest adhesion between basal compartments (Fgfbas-Fgfbas) allowed these FGF compartments to move more freely without getting impeded by excessive attachment to basal compartments of FGF cells ahead or behind them. Adhesion of FGF and Wnt cells to the underlying ECM, and adhesion between sheath cells and Wnt cells to the overlying skin cells, was set to provide adequate attachment without getting stuck to these substrates during migration. Strong internal adhesion was specified between adjacent compartments within the tripartite FGF cells to maintain their integrity and polarization. Structural integrity and apico-basal polarization of FGF cells were further reinforced by internal FPP links between adjacent compartments within each cell. Such internal FPP links were meant to simulate the overall effects of cytoskeletal elements that impart structure to cells. Weak adhesion was specified between cells and cell compartments not expected to be in contact with each other.

Simulations using the CPM model, based on the assumptions about selective cell adhesion described above, recapitulated many aspects of primordium behavior. First, the primordium cells migrated as a cohesive entity between the skin cells above and the ECM below. Second, the adhesivity of superficial sheath cells to the overlying skin, and of the Fgfbas compartments to the underlying ECM were high enough to keep them attached to these substrates, but low enough to allow detachment as the primordium moved forward. Third, as the primordium cells began to migrate, adhesive and spring-like interactions between FGF cells determined the spontaneous clustering of cells to form ‘rosettes’. While initially some of the rosettes were unstable, the system settled into a stable configuration with four rosettes (Fig. 5C; Movie 12). In ten runs of the simulation, the system achieved a stable configuration of three (6/10) or four rosettes (3/10) within 5000 mcs (Monte Carlo steps) (Fig. S3). In 1/10 simulations (Fig. S3E), the system underwent further reorganization into three rosettes from four at 10,000 mcs.

### Recapitulation of rosette reorganization following experimental manipulation

The CPM models enabled us to simulate experiments in which either Cxcl12a-dependent movement of leading or FGF-dependent movement of trailing cells was transiently compromised. As in the experiments, selective slowing of leading cells resulted in transient fusion of pre-existing, leading Wnt cells (Fig. 5D; Movie 13). At 5000 mcs, the simulation showed a stable pattern of four cell clusters. Stalling of the leading Wnt cells resulted in progressive fusion of pre-existing four rosettes to form first three and then two larger rosettes. Then, as migration of leading Wnt cells was restored, one of the fused rosettes split again to form two, recapitulating the phenomenon that had been seen *in vivo* (Fig. 1B; Movie 6). This phenomenon was observed in 5/5 simulations. Transient stalling of the trailing FGF cells in a simulation in which a stable pattern of three clusters was established by 5000 mcs caused stretching and eventual splitting of the rosette closest to the Wnt domain (Fig. 5E; Movie 14); 4/5 simulations were able to reproduce this phenomenon. In the example shown, the rosette remained split at 30,000 mcs even after trailing cell migration resumed at 20,000 mcs.

**Fig. 6.**
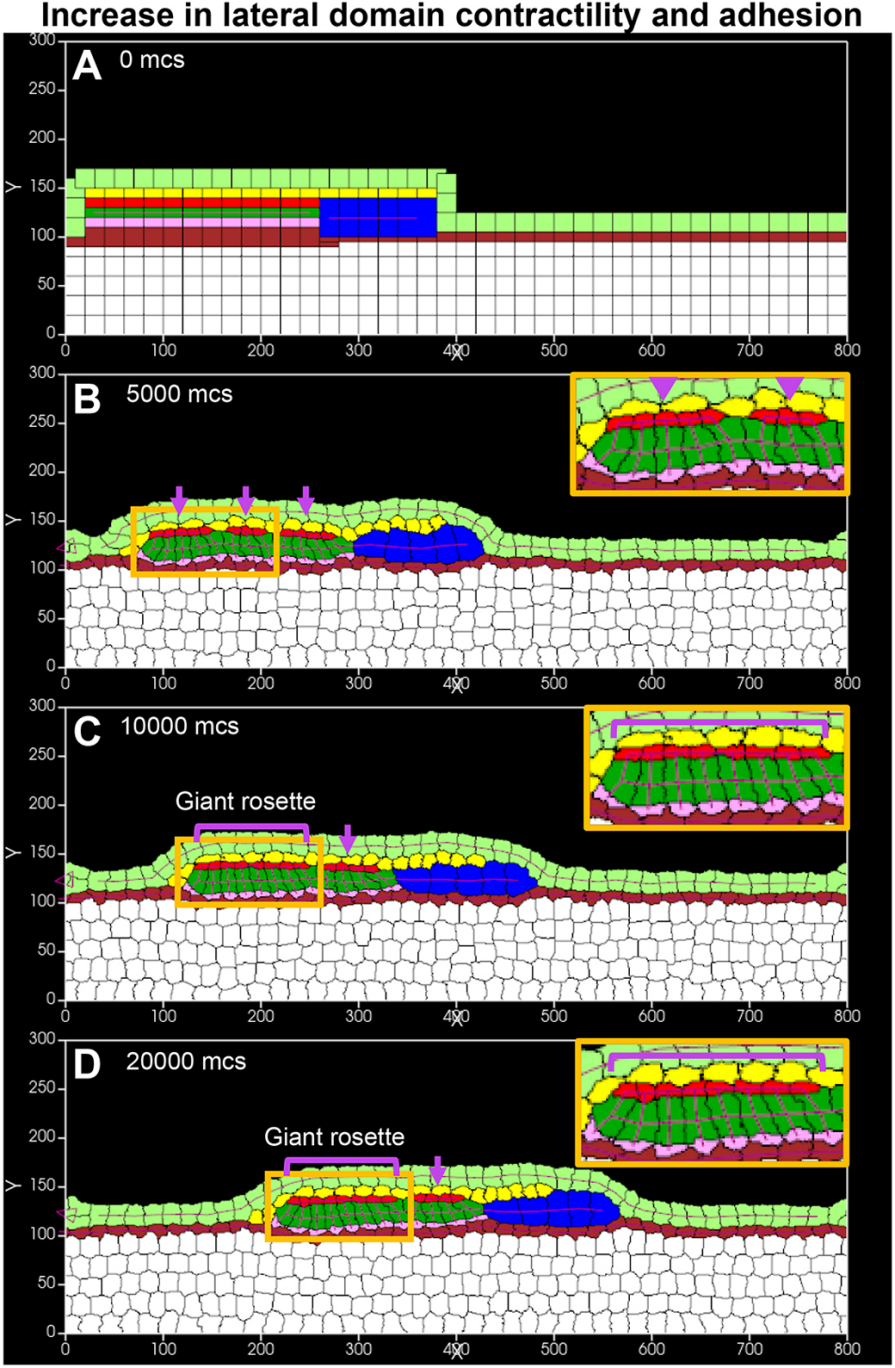
**Increasing cell–cell adhesion and contractility results in formation of giant rosettes.** (A-D) Example simulation in which lateral domain contractility (*λ*_*fgflat*−*fgflat*_=40) and cell–cell adhesion (*E*_*fgflat*−*fgflat*_=1) have been increased, showing start of simulation (A), and progressive fusion of two smaller rosettes into one giant rosette (B-D) (*n*=5/5 simulations). Smaller rosettes are marked with magenta arrows, and the relatively larger ones are denoted with magenta brackets. Orange boxes mark the regions shown in the insets. *x*-axis and *y*-axis represent the *x*- and *y*-dimensions (in pixels) of 2D space in which the model PLLp migrates.

Previous studies have shown that when Notch signaling is broadly activated within the entire primordium, there is a reconfiguration of the pattern of rosettes so that cells coalesce to form one or two large trailing rosettes (Kozlovskaja-Gumbriene et al., 2017). Notch activation in this context was shown to increase the expression of cell adhesion molecules and junctional proteins, suggesting that the reconfiguration of the rosettes was the result of enhanced adhesive interactions between neighboring cells (Kozlovskaja-Gumbriene et al., 2017). To recapitulate the effects of Notch activation, the contractility of FPP links between adjacent Fgflat compartments was increased from 10 to 40, and adhesion strength was increased by reducing of Fgflat-Fgflat Contact Energy from 5 to 1 (Supplementary Materials and Methods, Tables S4, S6). Such a change led FGF cells to gradually aggregate such that initially formed rosettes fused to form a large trailing rosette (Fig. 6; Movie 15), recapitulating observations of Kozlovskaja-Gumbriene et al.

## DISCUSSION

The periodic and sequential formation of protoneuromasts within the migrating primordium requires the coordination of two patterning events: one involving cell fate specification, whereby an *atoh1a*-expressing cell becomes specified as a sensory hair cell progenitor at the center of each nascent protoneuromast, and the other involving morphogenesis, whereby surrounding cells reorganize to form an epithelial rosette. Previous studies have shown that FGF signaling initiates both these events; first, it promotes *atoh1a* expression, which gives cells the potential to become sensory hair cell progenitors, and second, FGF signaling promotes epithelialization of cells and the expression of factors such as *shroom3*, which promote apical constriction and reorganization of cells to form epithelial rosettes (Ernst et al., 2012; Lecaudey et al., 2008; Nechiporuk and Raible, 2008).

The self-organization of center-biased FGF signaling centers in the wake of a shrinking Wnt activity domain (Dalle Nogare and Chitnis, 2020) create a pre-pattern of FGF signaling that initiates formation of protoneuromasts with their central *atoh1a*-expressing cell and surrounding cells organized to form epithelial rosettes. Once *atoh1a* expression becomes restricted to the central cell, it becomes the source of signals that activate FGF and Notch signaling in its neighbors, consolidating their organization as epithelial rosettes. While the FGF pre-pattern helps understand how specification of a central Atoh1a-expressing cell becomes coupled to surrounding cells that form epithelial rosettes, our observations suggest that these events can become uncoupled. Manipulations that selectively slowed the leading cells allowed the column of primordium cells to compress along its length and become more rounded; this was accompanied by pre-existing apical constrictions coming together to form fewer, larger rosettes. As leading cells resumed migration, single constrictions sometimes split to form two once again, reflecting the relative plasticity of cell clustering. Similarly, when trailing cells were selectively slowed, the column of primordium cells stretched, and pre-existing constrictions split to form two constrictions. These observations illustrate how epithelial rosette formation within the migrating primordium is ultimately dependent on mechanical interactions between primordium cells. While a pre-pattern of FGF signaling and the subsequent establishment of a stable central Atoh1a-expressing cell helps coordinate specification of a central sensory hair cell progenitor that is surrounded by cells that reorganize to form epithelial rosettes during periodic formation of neuromasts, primordium cells are primed to determine self-organization of epithelial rosettes independently of Atoh1a, and the size and number of the epithelial rosettes can be influenced by manipulations that influence the nature of the mechanical interactions between cells.

The relative independence of *atoh1a*-dependent sensory hair cell specification and epithelial rosette morphogenesis was suggested earlier by *atoh1a* knockdown experiments which showed that this does not prevent periodic epithelial rosette formation; the primordium continues to migrate, forming and depositing ‘neuromasts’ that form relatively unstable epithelial rosettes (Lecaudey et al., 2008). While FGF normally initiates formation of epithelial rosettes, it has been shown that a pre-pattern of FGF signaling activity is not required for self-organization of multiple epithelial rosettes in the migrating primordium. In primordia in which there was broad induced expression of an activated form of Notch, without any pre-pattern, a few relatively large rosettes formed (Kozlovskaja-Gumbriene et al., 2017). Similarly, when FGF signaling was inhibited in control primordia, epithelial rosettes disassembled, and their formation was inhibited. However, when FGF signaling was inhibited in primordia with broad ectopic Notch activation, cells self-organized to form about three rosettes in the absence of any pre-pattern of Notch or FGF signaling. Together, these observations suggest that, while FGF signaling may help initiate epithelial rosette formation, Notch activation is adequate to determine expression of factors that promote self-organization of primordium cells to form rosettes. This includes promotion of adhesion factors such as Cdh1 (E-cadherin) and EpCAM, along with tight junction components such as CldnB and CldnE (Kozlovskaja-Gumbriene et al., 2017), which are expected to promote adhesion and apical constriction. These observations show that while a pre-pattern of individual FGF signaling centers may more reliably initiate periodic formation of protoneuromasts with their central *atoh1a*-expressing cells and associated epithelial rosettes, broad unpatterned expression of factors that promote adhesion and apical constriction is adequate to determine self-organization of cell clustering within the migrating primordium.

To demonstrate that reorganization of apical constrictions following stretching or compression of the migrating primordium is independent of changes in the pre-pattern of FGF signaling, we initially compared the number of apical constrictions with the number and distribution of *atoh1a-*expressing cells in the primordium. The goal was to use *atoh1a* expression as a proxy for the FGF pre-pattern that coordinates epithelial rosette formation. While each constriction and its epithelial rosette is eventually associated with a single *atoh1a* cell (Fig. S4A), examination of over 100 primordia revealed that the number and pattern of *atoh1a* expression is much more dynamic than we appreciated, making the distribution of *atoh1a*-expressing cells an unreliable proxy for individual FGF signaling centers. While there were clear examples of primordia where a single apical constriction was associated with multiple *atoh1a*-expressing cells, potentially reflecting the fusion of pre-existing rosettes formed in response to the FGF pre-pattern, these data were excluded but an example is provided in Fig. S4B. Establishment of transgenic lines that report the dynamic pattern of *atoh1a* and FGF signaling in the primordium will be used in future studies to better define how stretching and compression of the primordium might also influence signaling and how the interplay between signaling and mechanics may be influencing dynamic reorganization of epithelial rosettes.

ABMs and CPMs were used to illustrate how mechanical interactions mediated by cell adhesion and apical constriction could determine self-organization of cell clustering along the length of the primordium independently of a pre-pattern that biases where they form. The underlying principle in both types of models is that adhesive interactions coupled with apical constrictions pull cells together to promote cell clustering. Unimpeded, this process is self-amplifying, and the clusters can grow progressively larger. However, cell attachment to the substrate or migratory behavior that stretches the primordium generates tension along its length, opposing cell aggregation. Together, the combination of local interactions that pull cells together, opposed by range inhibition via tension, determines self-organization of cell clusters. Conceptually, this self-organizing process can be thought of as a mechanical version of the same principles of local activation and long-range inhibition associated with Wnt and FGF signaling described earlier that have the potential to determine self-organization of FGF signaling centers. The mechanical interactions that determine the spontaneous formation of cell clusters and their similarity to reaction-diffusion mechanisms described independently by Turing and Meinhardt (Gierer and Meinhardt, 1972; Meinhardt, 2012) had been demonstrated by Oster et al. (1983) and by Harris et al. (1984) more than 50 years ago.

The NetLogo models represent complex apicobasally polarized cells as simple points in a 2D universe connected to their neighbors with spring-like links. The simplicity of these models made it easier to visualize how the mechanical interactions that pull cells together, coupled with tension that pull cells apart, can result in self-organized clustering. In the context of this simple 2D representation, clustering of turtles correlates with rosette formation. The development of the CPM helped extend the ideas developed with this initial simple representation with NetLogo turtles and links, through which cell shape, polarization, and distinctions between apical constriction and cell adhesion could not be represented. Nevertheless, the simple NetLogo model helped develop a conceptual framework for understanding how a balance of interactions that pull apical compartments together, coupled with tension along the length of the primordium associated with migration could influence the pattern of cell clustering to form ‘rosettes’. By representing FGF cells as tripartite composites with basal, lateral and apical compartments, the CPM model was better able to explore emergent organization because of interactions between these distinct compartments in adjacent cells. It was possible to visualize how, for a set of selected initial conditions, a relatively stable pattern of rosettes emerges, how slowing of leading or trailing domains results, respectively, in fusion or splitting of rosettes, and how increasing adhesive interactions between adjacent lateral compartments of FGF cells can promote their reorganization to form a large trailing cluster.

While our simulations recapitulate many of the experimental observations, these 2D representations remain caricatures and the primary strength of these models is conceptual. Much of the complexity of cell biology associated with apical constriction, rosette formation and collective migration is not incorporated in these models. Furthermore, while turtles associated with spring-like links in NetLogo, and energy minimization associated with target parameters in CPMs, simulate emergent organization and collective behavior of primordium cells, these models are not based on a literal representation of measured real-world physical interactions, space or time. Rather, these models illustrate the power of ABMs, which can be used to predict emergent organization based on rules that define how the agents interact and behave at different scales. Nevertheless, these models provide a conceptual framework for future models that could be developed when quantitative information related to physical interactions becomes available.

Previous analysis of signaling interactions mediated by the Wnt, FGF and Notch signaling pathways has shown how they can determine self-organization of periodic FGF signaling centers with central *atoh1a*-expressing cells (Dalle Nogare and Chitnis, 2020). Here, we have described how mechanical interactions between cells can also determine periodic cell clustering within the primordium. Although these patterning mechanisms could operate independently, they mutually amplify each other and eventually become synchronized. As *atoh1a* expression becomes self-sustaining through autoregulation in the central sensory hair cell progenitor in the most trailing mature protoneuromasts, it becomes a stable source of Delta and FGF, which activates Notch and FGF signaling in neighbors. This locks the relationship between the central *atoh1a*-expressing cell and surrounding cells, ensuring they form stable epithelial rosettes less susceptible to changes in tension along the length of the primordium. In this manner, while signaling and mechanics initiate the self-organization of potentially unstable protoneuromasts, their maturation, spearheaded by *atoh1a*, determines reliable, robust periodic formation and deposition of stable neuromasts by the migrating primordium.

The mechanisms that determine specification of a central sensory hair cell progenitor and reorganization of surrounding cells to form epithelial rosettes reflect the merging of two evolutionarily conserved patterning systems. The first is related to the mechanism described earlier in *Drosophila* development, whereby Notch-mediated lateral inhibition leads to the specification of a central progenitor cell within a proneural cluster (Muskavitch, 1994). Second, the combination of signaling and mechanical interactions that determine periodic clustering of cells to form epithelial rosettes has similarities to mechanisms that determine periodic clustering of cells to form feather buds and hair follicles in the skin of birds and mammals, respectively (Aman et al., 2018; Dalle Nogare and Chitnis, 2017b; Glover et al., 2017; Shyer et al., 2017). Together, the observations presented in this study illustrate how the zebrafish PLLp serves as an attractive model system for understanding how cell fate specification and tissue morphogenesis emerges from an interplay between signaling and mechanics. It also serves as a platform for recognizing the many ways in which conserved mechanisms have evolved and have been reused in combination to coordinate reproducible and robust processes in development.

## MATERIALS AND METHODS

### Zebrafish lines, embryo manipulation and drug treatment

Zebrafish embryos were generated by natural spawning, maintained under standard conditions (28.5°C), and staged according to Kimmel et al. (1995). Long-term time-lapse imaging was performed on *Tg(cldnb:lyn-egfp)* (Haas and Gilmour, 2006); *TgBAC(cxcr4b:h2a-mcherry)* (Wang et al., 2018);*Tg(hsp:sdf1a)* triple transgenic embryos. All embryos were between 27 and 28 hours post-fertilization when imaging started. Embryos used in the overexpression of *sdf1a* (Fig. 1A) or of *fgf10* (Fig. 2B) were heat-shocked in a water bath maintained at 37.5°C for 20 min. Non-heat-shocked triple transgenic embryos and wild-type double transgenic embryos [*Tg(cldnb:lyn-egfp);TgBAC(cxcr4b:h2a-mcherry)*] were used as controls. All embryos were anesthetized with MS-222 (stock 4 g/l; Syncaine, Syndel) mixed in 1:25 proportion (final 160 mg/l) in E3 medium and mounted in 1% low-melt agarose (NuSieve GTG). For PP1 drug treatment experiments (Fig. 2A), all embryos were anesthetized with MS-222 in E3 medium containing 20 μM PP1 and mounted in 1% low-melt agarose containing the same concentration of the drug. Imaging was started within 10 min of the end of heat shock or the addition of the drug. For the recovery of split rosettes experiment (Fig. 2C), embryos were treated with 20 μM PP1 as described above and imaged for 2 h before being heat-shocked directly on the microscope incubation stage at 37.5°C for 45 min. The duration of the on-stage heat shock had to be longer than that in a water bath to compensate for environmental heat dissipation and time taken for the incubation setup to reach the desired temperature.

### Time-lapse microscopy, image processing and quantification

Long-term time-lapse confocal images of embryos were acquired using a Nikon Ti2 inverted microscope with Yokogawa CSU-W1 spinning disk confocal and the Hamamatsu Orca Flash 4 version 3 camera with a 40×W 1.1NA objective. Expression data of *Tg(cldnb:lyn-egfp)* and *TgBAC(cxcr4b:h2a-mcherry)* were acquired with 488 nm and 561 nm excitation lasers, respectively. Images were acquired at 5-min intervals over a period ranging from approximately 2 to 14 h. The sample stage was manually shifted intermittently to prevent the sample from going out of the imaging frame. Acquired images were then stitched using a custom in-house macro in Fiji (Schindelin et al., 2012).

### PIV analysis

PIV analysis was carried out using PIVLab (Thielicke and Sonntag, 2021) in MATLAB [version: 24.2 (R2024b) Update 2; MathWorks]. Images were preprocessed in Fiji prior to importing into PIVLab. A Gaussian smoothing filter (σ=2.0) was applied to the nuclei channel of acquired time-lapse movies after background subtraction to reduce noise in the images. Binary masks of nuclei were created at each timepoint using the Yen thresholding method. Smoothed nuclei images were then subtracted from the generated nuclei masks. In a densely packed tissue, such as the zebrafish PLLp, this process led to the generation of nuclei outlines, as well as graded intensities within each nucleus, with the intention that this would eventually facilitate high-resolution PIV computation. These resulting images were then imported into PIVLab, where image contrast was enhanced using the CLAHE (contrast limited adaptive histogram equalization) algorithm with a window size of 64 pixels (Pizer et al., 1987) implemented in PIVLab. Image correlation was assessed in two passes, matching a 150×150 px^2^ window with a 75×75 px^2^ sliding window in the first pass, followed by matching a 75×75 px^2^ window with a 38×38 px^2^ sliding window in the second pass. The last pass was repeated until the quality slope was less than 0.025. Boundary effects were neglected during computation. The magnitudes of velocities in the direction of migration were then computed in PIVLab and exported as images. These images were then superimposed on binary nuclei masks generated as detailed earlier by utilizing the ‘AND’ image calculator function in Fiji.

### Computational modeling

#### ABMs

Agent-based modeling was carried out using the NetLogo programming environment (Wilensky, 1999). NetLogo allows users to define rules governing the behavior of three types of agents: turtles, patches and links. Turtles are agents that can move and can be used to represent different types (breeds) of cells. Patches are stationary agents resembling a grid over which turtles move. They can represent the extracellular environment and can be used to simulate diffusion of signaling molecules produced by cells and deposited on the patches. Links are agents that connect turtles and can be used to represent mechanical spring-like coupling between cells resulting from a combination of inter-cellular adhesion and apical constriction. The links can have turtle breed-specific parameters including a spring-constant (stiffness or resistance to length extension of a link), spring-length (the length all links try to achieve by pushing or pulling on connected nodes) and repulsion-constant (the force with which connected nodes push each other to avoid overcrowding). The Fruchterman–Reingold layout algorithm (Fruchterman and Reingold, 1991), used to model the layout of nodes in a network, integrates the influence of the spring parameters.

The specific parameters used in our NetLogo models, and their ranges are detailed in Table S1. Two ‘breeds’ of turtles were defined, WNTers and FGFers, to represent cells in leading Wnt active and trailing FGF active domains, respectively (Fig. 3). A third breed called Depositers was used in simulations described later, where progressive shrinking of the Wnt system and the periodic deposition of the cell clusters was incorporated (Fig. 4).

Various parameters could be set to determine the behavior of WNTers and FGFers. This includes their migration speed, proliferation rate and various parameters associated with links made with neighboring turtles. Among these parameters are the radius within which WNTers and FGFers could form links with neighbors, as well as the spring parameters of the links. In addition, the links were broken if they were stretched beyond a defined break-threshold or could be turned over at a low but defined rate (not used in the simulations shown in this study).

The PLLp model illustrated in Fig. 4 has three types of cells, WNTers (green), FGFers (yellow) and Depositers (red). To initiate the program, a column of turtles is created with defined width (Pllp-width=3) and length (Pllp-length=30). Links with defined spring properties are initially made with surrounding turtles. Pllp-length helps define initial Pllp-size. The relative size of the WNTer and FGFer fraction is defined by ‘wnt-fraction’ (0.6) and is used to define the initial length of the WNTer domain (‘wnt-size’). Whether Wnt fraction shrinks can be set by On/Off switch. If on, a slider defines after how many ticks (shrink-cycle=90) the Pllp-size shrinks by one patch. Then the wnt-size is shrunk to keep the leading domain of WNTers at the desired fraction. The trailing FGFer domain is defined as the turtles within the column of turtles of length Pllp-size that are not WNTers. Turtles in positions trailing the defined Pllp-size are designated as Depositers.

At each ‘tick’ of the program, each turtle makes links with other turtles within a defined radius, specific to each breed. In Fig. 4, links were made with any turtle within a two-patch radius (wnt-radius=fgf-radius=depositer-radius=2). Links are broken if they stretch a defined multiple of their defined spring-length (spring-length*break-threshold). Finally, links are maintained or re-made with turtles within the radius defined for that breed of turtle (wnt-radius, fgf-radius, depositer-radius).

At each tick, WNTers and FGFers move forward a distance defined by their speed (patches/tick). For the model in Fig. 4, WNTer speed (cxcl12a-speed) was 0.05 patches/tick, FGFer speed (fgf-speed) was set at 0.0001 patches/tick or 0. Depositer speed was always 0. With this slow speed, FGFer migration was dependent on mechanical coupling with leading WNTers through links and it determined effective stretching of the model primordium. Furthermore, ‘deposition’ of Depositers was determined not so much by their slow speed but rather by the low threshold for breaking links (depositer-break-threshold=1) wide, as opposed to links between WNTers and FGFers whose links were relatively resistant to breaking (Wnt-break-threshold=Fgf-break-threshold=10). This illustrates how, while deposition can be determined by setting the migration speed for Depositers at 0, it can also be determined by regulating mechanical coupling and break-thresholds.

During the simulation, the organization and movement of turtles in the primordium can be visualized in two ways: the turtles can be visualized by distinct breed color (WNTers, green; FGFers, yellow; Depositers, red), or in shades of red reflecting the density of turtles. This ‘density’ mode makes it easier to visualize patterns of spontaneous clustering of turtles under various conditions. In this mode, when turtle (hexagon-shaped) packing exceeds a threshold density, the turtles are given a cyan color to highlight the pattern of cell clustering in the model primordium (Fig. 3A). An aggregate containing at least three cyan cells was defined as a cluster for quantification purposes in Fig. 3E,F and Fig. S2C,D. Discrete clusters had at least one non-cyan cell separating two cyan cells. Five independent simulation runs per condition were performed.

#### CPMs

We implemented CPMs in the CompuCell3D software package (Swat et al., 2012). The CPM framework allows one to simulate changes in cell morphologies based on energy minimization. Total system energy *J* is calculated as:
(1)

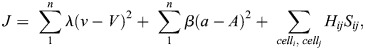
where *λ* is the degree of penalty imposed if the volume of the cell (*v*) varies from the target volume (*V*). Similarly, *β* is the degree of penalty imposed for variation of cell area (*a*) from target area (*A*). *H*_*ij*_ and *S*_*ij*_, where the subscripts *ij* refer to *cell*_*i*_ and *cell*_*j*_, together represent cell–cell adhesion energy. Change in energy (Δ*J*) over a Monte Carlo step (mcs) can be represented as:
(2)

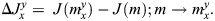
Since Δ*J* in the CPM framework is translated into a pixel copy, thereby effecting a change in system shape, the probability of accepting such a pixel copy is determined by:
(3)

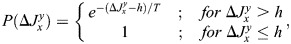
where *h* represents an energy threshold and *T* represents fluctuation temperature, a parameter governing stochastic dynamics of the system. Therefore, the probability of accepting a pixel copy increases with a decrease in 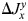 or an increase in *T*.

We created a two-dimensional simulation environment (800 pixels×300 pixels×1 pixel) square lattice with periodic boundary conditions in the *x*-dimension to prevent the unintended effects of artificial rigidity and adhesivity in the system that might occur due to a fixed boundary (Fig. 5B). The 2D Cellular Potts primordium model was developed representing the primordium in a side-view (Fig. 5A), migrating between skin cells (light green) above and a layer of cells representing a thin layer of ECM (brown) below, as has been recently characterized by Yamaguchi et al. (2022). In addition, below the ECM cells is a layer of cells representing a combination of the deeper myoseptum and muscle cells (white). Here again, the muscle layer was made thick enough (five cells thick vertically), to minimize artificial rigidity effects of the system boundary on the model primordium. The primordium itself is made of three types of cells: (1) a leading domain of Wnt cells (blue), representing cells with a relatively non-apicobasally polarized morphology that migrate in response to a self-generated chemokine gradient, (2) a trailing set of FGF cells [a composite of red (apical; Fgfapi), green (lateral; Fgflat) and pink (basal; Fgfbas) domains], represents polarized epithelial cells with apicobasal polarity that apically constrict and move in response to FGF signals from the leading domain, and (3) a set of sheath cells (yellow) between the deeper Wnt and FGF cells and below the overlying skin cells. Previous studies have shown these superficial sheath cells are essential for effective collective migration of the primordium in confinement between skin above and ECM below (Dalle Nogare et al., 2020). An additional cell type called ‘Medium’, a mandatory default cell type in CompuCell3D, signifying all the remaining space in the system, is also included in the model.

‘Volume’ and ‘Surface’ plugins were used to specify 2D surface area and perimeter constraints, respectively, for each cell type. The ‘Contact’ plugin helped us specify the relative adhesive energies between the various cell types. Cells that have relatively high contact energy defined between them are less likely to adhere to each other than cells that have low contact energy. Additionally, the internal adhesive energies between the three compartments of FGF cells were defined using the ‘ContactInternal’ plugin. Migratory forces (using the ‘lambdaVecX’ property within the ‘ExternalPotential’ plugin) were imparted only to Wnt and sheath cells and Fgfbas domains of FGF cells to simulate chemokine-dependent migration of Wnt cells and the influence of FGF signaling on sheath cells and the basal feet of FGF cells. A negative lambdaVecX value represents the amount of force applied on the cell along the positive *x*-direction.

The ‘FocalPointPlasticity’ plugin in CompuCell3D was used to create intercellular spring-like links between neighboring cells of the following types: skin-skin, Wnt-Wnt, Fgflat-Fgflat, Fgfapi-Fgfapi and ECM-ECM. To maintain cohesive migration of the model primordium as a single unit, the last Wnt cell and the first Fgflat cell from the leading end were connected by a spring-like link. This plugin was also used to generate intracellular links within FGF cells linking the two adjacent pairs of domains (Fgfapi-Fgflat and Fgflat-Fgfbas), thereby simulating cytoskeletal elements within cells that impart structural rigidity and apicobasal elongation. Three parameters must be defined to simulate a spring-like link, namely: strength (equivalent to spring constant, *λ*), target distance (the length a link tries to attain, *L*), and maximum distance (the maximum length a link can stretch to before it breaks). Tuning these three parameters helped us achieve varied behaviors such as cell-scale or tissue-scale contractility, cell–cell adhesivity, cohesive collective migration, and structural rigidity. Incorporation of these links changed Eqn 1 into Eqn 4:
(4)

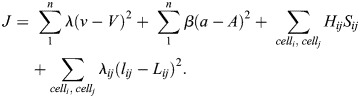
Once intercellular links between neighboring Fgfapi compartments extended beyond their maximum length, they broke and could only be re-established if those neighboring compartments came into contact again over time. Such dynamic remodeling of the Fgfapi-Fgfapi contractile links allowed us to simulate cell–cell interactions and rosette formation in the FGF domain. The ‘NeighborTracker’ plugin is a helper module for the ‘FocalPointPlasticity’ plugin and was used to track all the neighbors of every cell in the system. All the parameters and variables in the model used in the current study are detailed in Tables S2-S5.

### Rationale for relative values of model parameters in the CPMs

#### Relative adhesivity of Wnt and FGF cells to their environment

As there was no explicit data about relative adhesivity between different types of cells in the primordium, some educated guesses about the relative strength with which different cell types or cell compartments are likely to adhere to each other, helped define relative contact energy associated interaction between different types of cells: low contact energy is associated with strong adhesion between cell types and high contact energy is associated with low adhesivity (Table S3). Although the complexity of the system prevented us from exhaustively testing a very wide range of alternate relative adhesive interactions, the iterative tweaking of adhesion parameters, starting with some basic assumptions, allowed us to converge on parameters that resulted in the model primordium recapitulating many aspects of its *in vivo* behavior. These are not exclusive parameters and relative adhesivity in the model matters more than the absolute contact energy associated with each type of contact. The broad rationale for defining specific contact energies used for most simulations presented in this study is described below.

As there was minimal mixing of different cell types within the system, it was assumed that cells adhere best to cells of the same type. So, contact energy between many cells of the same type was set low (Wnt-Wnt=1; Fgfapi-Fgfapi=1; Muscle-Muscle=1). To make sure the migrating primordium does not break through the single-cell layer of overlying skin cells, contact energy associated with skin-skin contact was set at 0 to ensure especially strong adhesion. Similarly, ‘cells’ representing underlying ECM cells, also had strong adhesion to each other to prevent the primordium from breaking through this substrate. However, as the ECM cells lay above a multilayer bed of muscle cells that provided an additional barrier to the migrating primordium, adhesion between ECM was not required to be as strong as between skin cells and contact energy for ECM-ECM was set at 0.5.

To make sure the leading domain of Wnt cells did not separate from trailing column of FGF cells, the contact energy associated with interactions between Wnt cells and the lateral compartment of trailing FGF cells (Wnt-Fgflat) was set relatively low at 0.5. The Wnt and FGF domain cells, are in discrete compartments in this model, which makes them vulnerable to separation without such strong adhesion. However, changes in adhesion between leading Wnt active and trailing FGF active domains may be more gradual *in vivo*, and the especially strong adhesion required between leading Wnt and trailing FGF cells, required in this model, may not be required *in vivo*.

Contact energy representing adhesive interaction between Wnt cells or the basal compartment of FGF cells (Fgfbas) and the underlying ECM cells was low enough to allow determine strong adhesive traction and facilitate migration (Wnt-ECM=5; Fgfbas-ECM=2). Adhesion between the Fgfbas compartment of trailing FGF cells and the underlying ECM was stronger than that between leading Wnt cells and the underlying ECM. Although established empirically in the model, it potentially corresponds to reported differences in traction reported between trailing and leading cells with the underlying ECM (Yamaguchi et al., 2022). However, in our simulations, this difference was not essential for the model primordium to recapitulate *in vivo* behavior as described.

As the apicobasally polarized FGF cells were represented as a composite of apical, lateral and basal compartments (Fgfapi, Fgflat, Fgfbas), internal contact energy was used to define adhesion between these compartments within each FGF cell. Internal contact energy for contacts between juxtaposed FGF cell compartments was low (Fgfapi-Fgflat=1; Fgflat-Fgfbas=1). By contrast, apical and basal compartments of an individual FGF cell, which are not expected to be in contact with each other, were associated with relatively high contact energy (Fgfapi-Fgfbas=10). This ensured that each multicompartment FGF cell migrated as a single cohesive entity and maintained its polarized structure.

Contact energies associated with adhesion between adjacent FGF cells were specified individually for each of its compartments. Adhesion was strongest between adjacent apical compartments (Fgfapi-Fgfapi=1), representing strong adhesion mediated by apical junctional complexes, weaker but still strong adhesion between adjacent lateral domains (Fgflat-Fgflat=5), representing adhesion mediated by additional adhesion molecules expressed on lateral surfaces, and weakest between adjacent basal compartments (Fgfbas-Fgfbas=8), which represented basal lamellipodia. These progressive changes in adhesion along the apicobasal axis between compartments of adjacent FGF cells facilitated adequate adhesion to determine effective cohesive migration of FGF cells. However, especially strong adhesion between apical compartments facilitated self-organization of FGF cell clusters or rosettes. The relatively weak adhesion between adjacent FGF basal compartments facilitated migratory behavior of individual FGF cells with the basal compartments simulating the behavior of lamellipodia as they move with relatively strong adhesion to the underlying ECM in response to FGF signals.

To maintain overall organization of the system, cells and cell compartments of adjacent cells that should not be in contact were assigned relatively high contact energies. For example, contact energies associated with Fgfapi-Fgfbas, Fgfapi-skin, Fgfapi-ECM, Fgfapi-muscle, Fgflat-ECM, Fgflat-skin, Fgflat-muscle, Fgfbas-skin and sheath-muscle were set at 20.

#### Relative contractility and adhesivity of Wnt and FGF cells

Contractility was prescribed to cells via the FPP links as described in the previous section. Since constrictions and rosettes are observed in the middle and trailing domains in wild-type control PLLps, we prescribed the highest spring strength (*λ*) and lowest target distance (*L*) to the apical compartments of FGF cells in the model. *λ* and *L* were higher and lower, respectively, for the lateral compartments of FGF cells relative to the unpolarized Wnt cells. A spring link with intermediate *λ* and *L* connecting the last Wnt cell and the lateral compartment of the first FGF cell from the leading edge was created to ensure cohesive migration of the model PLLp. Short ‘maximum distance’ values simulate dynamic breaking and re-forming of Fgfapi-Fgfapi FPP links, thereby allowing us to visualize clustering and re-organization of rosettes, whereas large values (Fgflat-Fgflat) ensured that the FPP links connecting them did not break.

#### Spring links in the skin and ECM

Spring parameters were chosen such that they function to simply maintain integrity of the skin and ECM in our model. These springs have low *λ*, high *L* and high maximum distance.

#### Model parameters for sheath cells

Sheath cells in the model were not connected via springs, as they have been observed to be a much more motile population than the rest of the primordium (Dalle Nogare et al., 2020). For this reason, we placed weaker constraints on their perimeter and surface area than the other cells in the model PLLp. They were also set to be weakly adhesive to each other to promote motility (sheath-sheath=6).

### Statistical analyses

Statistical significance for each condition in Fig. 2D-F was calculated between −30 and 0 using two-tailed, paired *t*-tests. Two-way ANOVA, followed by Tukey's multiple comparisons test between the three conditions in Fig. 3C, was performed to test statistical significance in Fig. 3D-F. Normality of all datasets was assessed by Shapiro–Wilk test, and they were all found to be normally distributed.

## Supplementary Material



10.1242/develop.204973_sup1 Supplementary information
