# Supplementary material for: Mechanical interactions dynamically influence rosette morphogenesis in the migrating zebrafish posterior lateral line primordium
Source: Development. 2026 Jul 15;153(13):dev204973. doi: 10.1242/dev.204973 (PMC13405218; doi:10.1242/dev.204973)
Supplement: Supplementary information [file develop-153-204973-s1.pdf]

Development: doi:10.1242/dev.204973: Supplementary information

Supplementary Materials and Methods

Netlogo code

```
globals  [repetitions] ;Variable to assist with creating movies of
simulations
breed [wnters wnter] ;Leading wnt zone cells
breed [fgfers fgfer] ;Trailing fgf zone cells
breed [depositers depositer] ;Deposited cells


to startup ;Startup procedure is automatically run when the model is opened
  setup
end

to setup ;Setup resets the simulation and prepares it to be run

  clear-all
  set-default-shape turtles cell-shape
  ask patches [
    if abs pycor < pllp-width and pxcor < ((-1 * max-pxcor) + pllp-length)
[sprout 1] ;Spawns turtles in a rectangle with set proportions
  ]
  ask patches [set pcolor black]
  set pllp-size pllp-length
  set wnt-size pllp-length * wnt-fraction
  set-breeds
  ask wnters [ ;Only turtles with the breed "wnters" follow these commands
    create-links-with other turtles in-radius wnt-radius ;Wnter turtle
creates links with turtles that are less than or equal to a set distance
    color-turtles
  ]
  ask fgfers [ ;Only turtles with the breed "fgfers" follow these commands
    create-links-with other turtles in-radius fgf-radius ;Fgfer turtle
creates links with turtles that are less than or equal to a set distance
    color-turtles
  ]
  ask depositers [ ;Only turtles with the breed "depositers" follow these
commands
    create-links-with other turtles in-radius depositer-radius ;Depositer
turtle creates links with turtles that are less than or equal to a set
distance
    color-turtles
  ]
  ask links [set color grey]
  reset-ticks

end


to set-breeds ;Sets the breeds of the turtles based on their location and
sizes of wnt/fgf zones

  ask turtles [
    if xcor > max [xcor] of turtles - wnt-size [
```

Development • Supplementary information

```
      set breed wnters ;sets leading zone turtles breed to wnter
      color-turtles
    ]
    if (xcor >= max [xcor] of turtles - pllp-size) and (xcor <= max [xcor] of
turtles - wnt-size) [
      set breed fgfers ;sets trailing zone turtles breed to fgfer
      color-turtles
    ]
    if xcor < max [xcor] of turtles - pllp-size [
      set breed depositers ;sets deposited cells to depositers
      color-turtles
  ]]

end



to shrink-wnt-domain ;Shrinks the size of the leading wnt zone at a set rate

  if ((ticks mod shrink-cycle) = 0) and (wnt-size >= 0) [
    set pllp-size (pllp-size - 1)
    set wnt-size (pllp-size * wnt-fraction)
  ]

end

to color-turtles ;Colors the turtles based on their breeds or density

  set size cell-size
  ifelse show-density? [
    set color scale-color red (count turtles in-radius 3) 0 density-scale
;Density is measured by counting the number of turtles within 3 units of this
turtle
      if count turtles in-radius 3 > cluster-threshold [set color cyan]
  ][
    if breed = wnters [
      set color green
    ]
  if breed = fgfers [
      set color yellow
    ]
  if breed = depositers [
      set color red
  ]]

end

to go ;When the "go" button is pushed on the interface, is procedure is run
repeatedly until the button is pressed again
      ;to run this code only once, press "go one step"
  if  max [xcor] of turtles >  max-pxcor - 1 [stop]
  set-breeds
  ask wnters [
    if ticks > migration-delay [
      set heading 90 ;sets the wnter forward direction to the right
```

```
        fd cxcl12a-speed ;moves wnter forward by set amount
      ]
      ask my-links [
        if link-length > wnt-break-threshold * wnt-spring-length [die] ;Breaks links if they stretch too far
      ]
      if random 100 < wnt-turnover [
        ask my-links [die] ;Randomly breaks links
      ]
      create-links-with other turtles in-radius wnt-radius
      layout-spring link-neighbors my-links Wnt-spring-constant Wnt-spring-length Wnt-repulsion-constant ;Arranges the turtles that are linked to this turtle as if their links are springs with the set variables
      color-turtles
    ]

    ask fgfers [
      if ticks > migration-delay [
        set heading 90 ;sets the fgfer forward direction to the right
        fd fgf-speed ;moves fgfer forward by set amount
      ]
      ask my-links [
        if link-length > fgf-break-threshold * fgf-spring-length [die] ;Breaks links if they stretch too far
      ]
      if random 100 < fgf-turnover [
        ask my-links [die] ;Randomly breaks links
      ]
      create-links-with other turtles in-radius fgf-radius
      layout-spring link-neighbors my-links fgf-spring-constant fgf-spring-length fgf-repulsion-constant ;Arranges the turtles that are linked to this turtle as if their links are springs with the set variables
      color-turtles
    ]

    ask depositers [
      ask my-links [
        if link-length > depositer-break-threshold * depositer-spring-length [die] ;Breaks links if they stretch too far
      ]
      if random 100 < depositer-turnover [
        ask my-links [die] ;Randomly breaks links
      ]
      create-links-with other turtles in-radius depositer-radius
      layout-spring link-neighbors my-links depositer-spring-constant depositer-spring-length depositer-repulsion-constant ;Arranges the turtles that are linked to this turtle as if their links are springs with the set variables
      color-turtles
    ]

    ask links [set color grey] ;Recolors new links to be grey

    if wnter-proliferate? [
      if count wnters > 0 [
```

```
      ask one-of wnters [
        if (random 100 / 1000) < proliferation-rate [
          hatch-wnters 1 [set ycor 0] ;Creates a new wnter turtle
  ]]]]

  if shrink-wnt-zone = true [shrink-wnt-domain]

  if fgfer-proliferate? [
    if count fgfers > 0 [
      ask one-of fgfers [
        if ( random 100  / 1000 ) < proliferation-rate [
          hatch-fgfers 1 [set ycor 0] ;Creates a new fgfer turtle
  ]]]]

  tick ;adds 1 to tick counter

end
```

Netlogo interface with set up for Figure 3

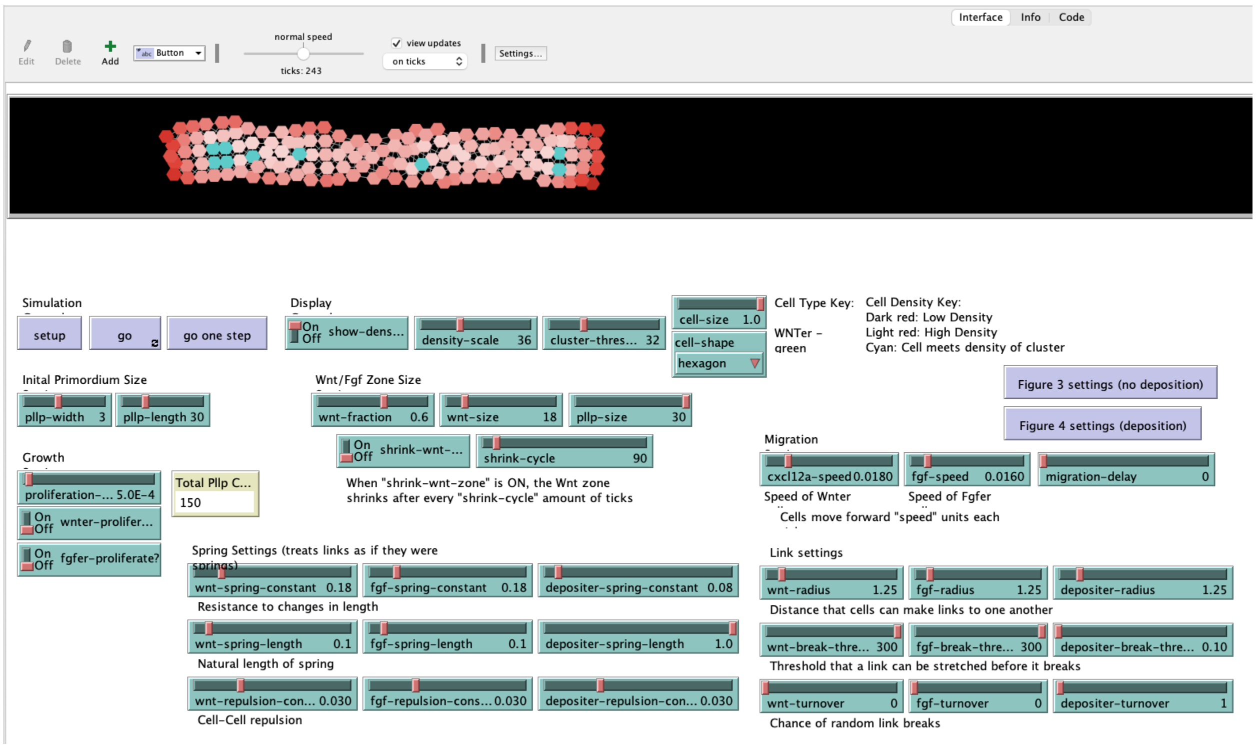

Available for download at
https://journals.biologists.com/dev/article-lookup/doi/10.1242/dev.204973#supplementary-data

Development • Supplementary information

Table S1. Details of the agent-based model created in NetLogo for Fig. 3

Variable Name	Physiological representation	Value	Units
cxcl12a-speed	Speed of leading cells	0, 0.015 – 0.018	patches/tick
fgf-speed	Speed of trailing cells	0, 0.015 – 0.018	patches/tick
pllp width	PLLp width	5	patches
pllp length	PLLp length	30	patches
proliferation-rate	Rate of cell divisions	5×10^{-4}	$tick^{-1}$
wnt-fraction	Fraction of PLLp with high Wnt activity	0.6 or 1	-
wnt-size		30	
pllp-size		30	
wnt-spring-constant	Adhesivity + Contractility of leading cells	0.14 – 0.2	-
fgf-spring-constant		0.14 – 0.2	-
wnt-spring-length	Refers to the region of influence of intercellular adhesion in the leading and trailing domains	0.1	patches
fgf-spring-length		0.1	patches
wnt-repulsion-constant	Refers to how close cells are allowed to come before being repelled	0.03	-
fgf-repulsion-constant		0.03	-
wnt-radius		1.25	patches
fgf-radius		1.25	patches
wnt-break-threshold	Determines stretch threshold (link length) at which adhesive interaction is lost (links die)	300	
fgf-break-threshold		300	
cluster-threshold	For visualization purposes only	32	number of turtles
density-scale	For visualization purposes only	36	number of turtles

Development • Supplementary information

Netlogo interface with set up for Fig. 4

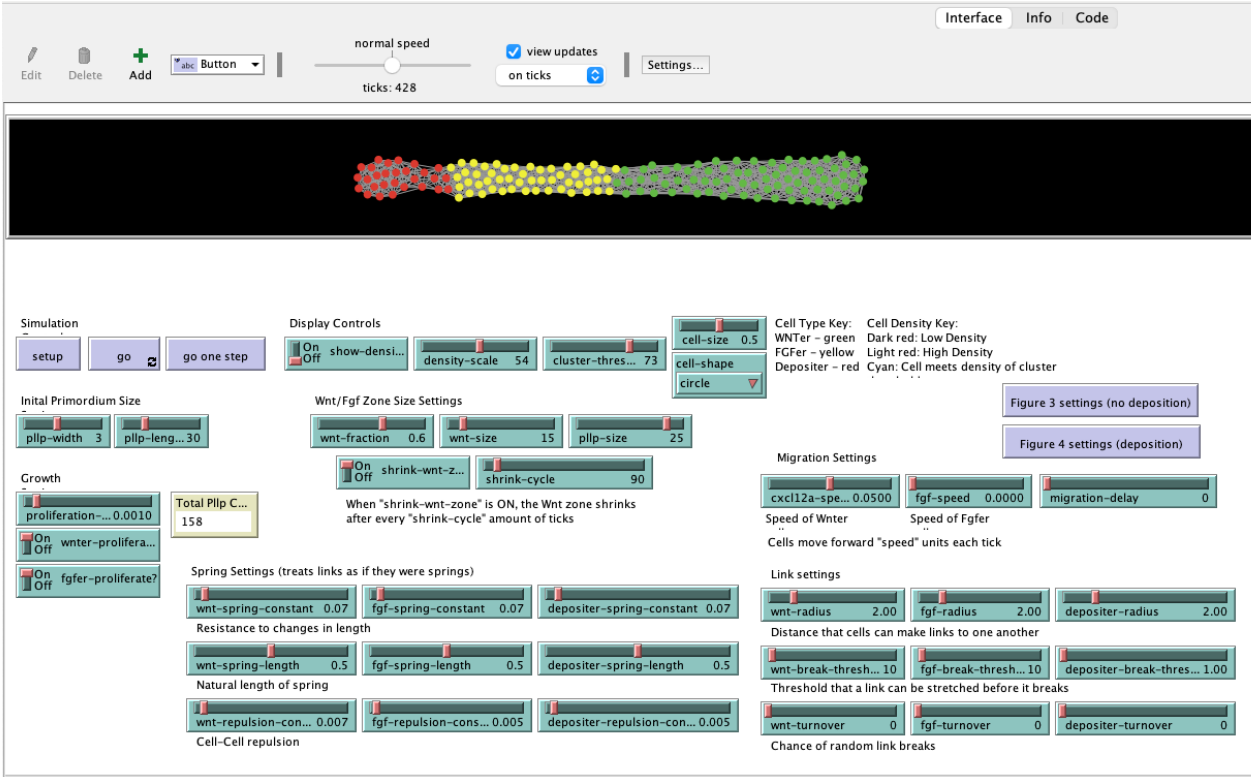

Available for download at
https://journals.biologists.com/dev/article-lookup/doi/10.1242/dev.204973#supplementary-data

Development • Supplementary information

Table S2. Details of the agent-based model created in NetLogo for Fig. 4

Variable Name	Physiological representation	Value	Units
cxcl12a-speed	Speed of leading cells	0.050	patches/tick
fgf-speed	Speed of trailing cells	0.000	patches/tick
depositer-speed	Speed of depositing cells	0	
pllp-width	PLLp width	5	patches
pllp-length	PLLp length	30	patches
proliferation-rate	Rate of cell divisions	0.001	$tick^{-1}$
wnt-fraction	Fraction of PLLp with high Wnt activity	0.6	-
wnt-size		30	
pllp-size		30	
wnt-spring-constant	Adhesivity + Contractility of leading cells	0.07	-
fgf-spring-constant		0.07	-
depositer-spring-constant		0.06	
wnt-spring-length	Refers to the region of influence of intercellular adhesion	0.5	patches
fgf-spring-length		0.5	patches
depositer-spring-length		0.5	patches
wnt-repulsion-constant	Refers to how close cells are allowed to come before being repelled	0.007	-
fgf-repulsion-constant		0.005	-
depositer-repulsion constant		0.005	
wnt-radius	Efficacy with which adhesive interactions form within radius of cell	2.0	patches
fgf-radius		2.0	patches
depositer-radius		2.0	
cluster-threshold	For visualization purposes only	32	number of turtles
density-scale	For visualization purposes only	36	number of turtles

Table S3. Cell shape constraints of the CPM created in CompuCell3D

Parameter	Cell type	Strength (λ)	Target Value
Surface Area ($pixels^2$)	Sheath	0.2	200
	Wnt	2	800
	Fgfapi	2	100
	Fgflat	0.1	400
	Fgfbas	2.5	100
	ECM	2	200
	Skin	2	400
	Muscle	20	400
Perimeter (pixels)	Sheath	0.2	100
	Wnt	0.3	120
	Fgfapi	0.3	25
	Fgflat	0.5	100
	Fgfbas	0.2	25
	ECM	0.8	80
	Skin	1	100
	Muscle	1	80

Table S4. Cell-cell adhesion energies within the CPM

Parameter	Cell-Cell interaction	Contact Energy
Contact Energies	Wnt-Sheath	6
	Wnt-Wnt	1
	Wnt-Fgfapi	3
	Wnt-Fgflat	0.5
	Wnt-Fgfbas	3
	Wnt-ECM	5
	Wnt-Skin	5
	Wnt-Muscle	10
	Fgfapi-Sheath	6
	Fgfapi-Fgfapi	1
	Fgfapi-Fgflat	10
	Fgfapi-Fgfbas	20
	Fgfapi-ECM	20
	Fgfapi-Skin	20
	Fgfapi-Muscle	20
	Fgflat-Sheath	10
	Fgflat-Fgflat	[1, 5]
	Fgflat-Fgfbas	10
	Fgflat-ECM	20
	Fgflat-Skin	20
	Fgflat-Muscle	20
	Fgfbas-Sheath	5
	Fgfbas-Fgfbas	8
	Fgfbas-ECM	2
	Fgfbas-Skin	20
	Fgfbas-Muscle	10
	ECM-Sheath	8
	ECM-ECM	0.5
	ECM-Skin	6
	ECM-Muscle	1
	Sheath-Sheath	6
	Sheath-Skin	4
	Sheath-Muscle	20

Development • Supplementary information

	Skin-Skin	0
	Skin-Muscle	10
	Muscle-Muscle	1
Internal Contact Energies (only for compartmental FGF cells)	Fgflat-Fgfbas	1
	Fgfapi-Fgflat	1
	Fgfapi-Fgfbas	10

Table S5. Cell Migration details in CPM

Cell type	Migratory Force (λ_x) (+x-direction)	Cell fluctuation amplitude
Wnt	[-0.1, -2]	[10, 50]
Fgfbas	[-0.1, -3]	[10, 30]
Sheath	-1	50

Table S6. *FocalPointPlasticity* links details in CPM

Cell-cell interaction	Strength (λ)	Target distance (L) (pixels)	Maximum distance (pixels)
Wnt-Wnt	5	10	200
Wnt-Fgflat	10	30	40
Fgfapi-Fgfapi	5	8	20
Fgflat-Fgflat	[10, 40]	8	100
Skin-Skin	10	15	100
ECM-ECM	4	10	40
Fgfapi-Fgflat	8	15	30
Fgflat-Fgfbas	8	15	30

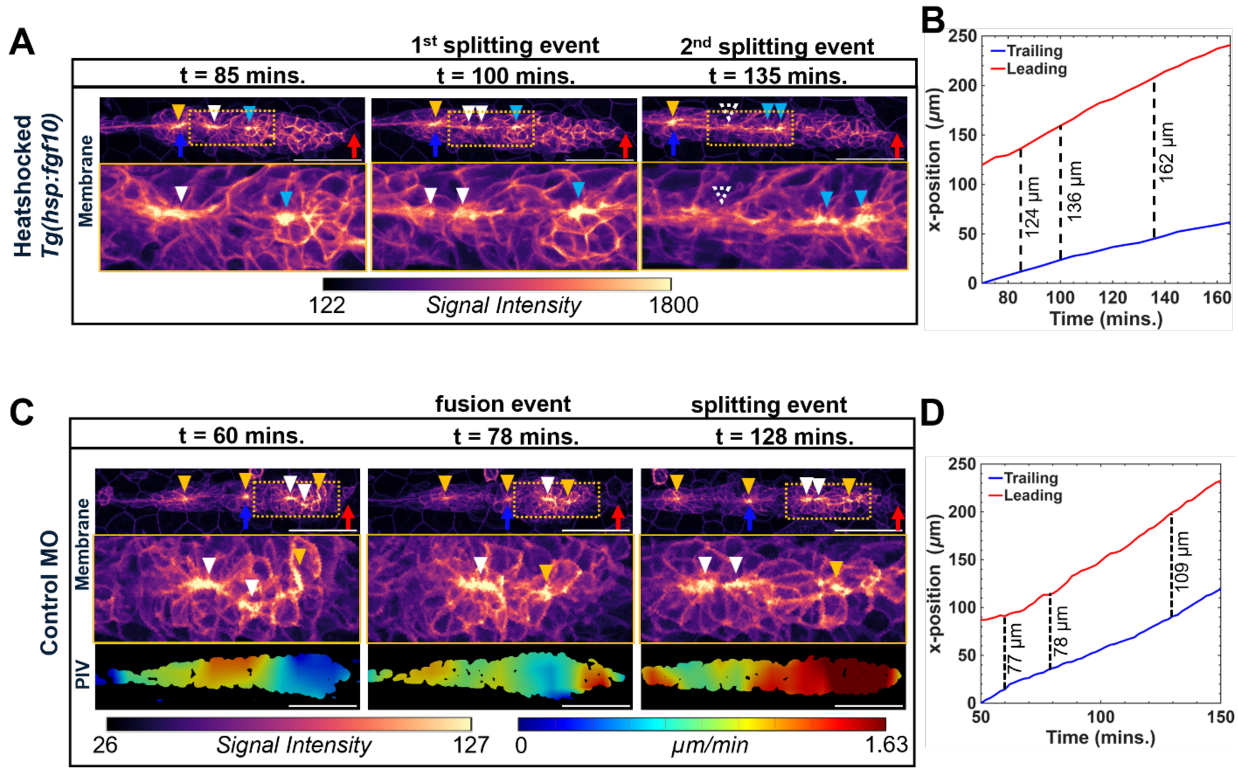

Fig. S1. (A) The heatshocked *Tg(hsp:fgf10)* PLLp images from Fig. 2B are reproduced here. Lower panels show magnified views of regions marked with dotted rectangles in the top panels. White arrowheads indicate the first constriction that splits (middle column) and then dissolves (dotted arrowhead, right column), while cyan arrowheads denote the second instance of constriction splitting (right column). Blue and red arrows indicate the trailing constriction and leading cell respectively, which were tracked over time. **(B)** Stretching of the PLLp in the direction of migration **(A)** as measured by tracking the x-positions (x-coordinates) of the leading cell (red curve) and the trailing constriction (blue curve) over time. Dashed lines correspond to the time points shown in (A). PLLp length is quantified and noted alongside the dashed lines at the 3 time points. Scale bars = 50 μm.

Development • Supplementary information

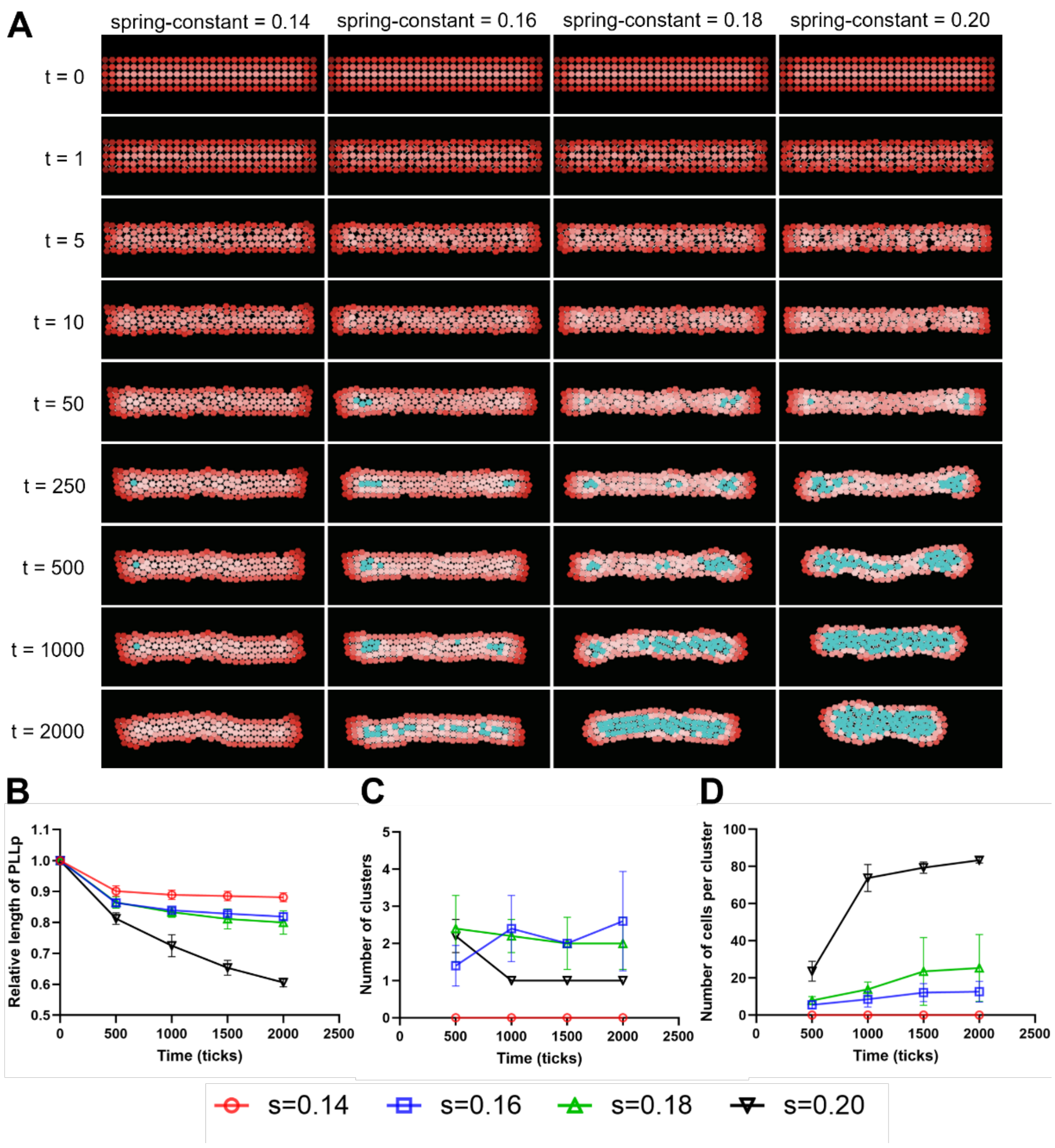

Fig. S2. (A) Evolution of turtle aggregation dynamics over time as a function of spring-constant (s) of links. Migration was not imparted to the turtles in these simulations. The third column is also shown in Fig. 3C. **(B)** Change in the relative length of model PLLp over time as compared to its original length at the start of simulation for the 4 conditions (s = 0.14, red; s = 0.16, blue; s = 0.18, green; s = 0.20, black) in **(A)**. **(C)** Variation in the number of clusters over time. An aggregate containing at least 3 cyan cells was defined as a cluster for quantification purposes. Discrete clusters had at least 1 non-cyan cell separating 2 cyan cells. **(D)** Number of cyan cells per cluster for each of the 4 conditions. **(B-D)** Data is presented by combining 5 independent simulation results for each condition. Datapoints represent the mean while error bars signify standard deviation.

Development • Supplementary information

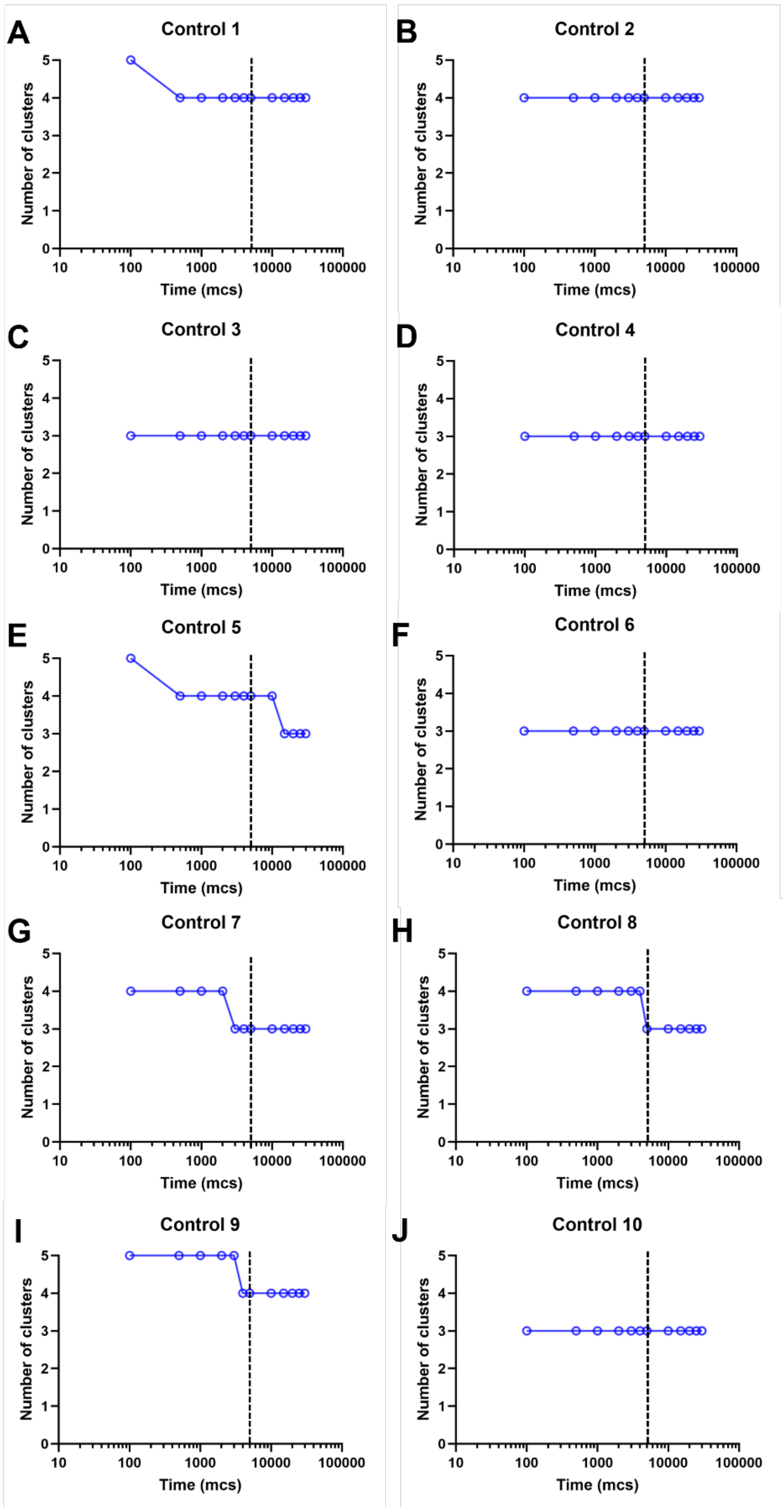

Fig. S3. (A-J) Variation in the number of clusters in the "Wild-type" Control CPM quantified over time in 10 replicate simulations of the wild-type control PLLp. Black dashed lines indicate simulation time = 5000 mcs.

Development • Supplementary information

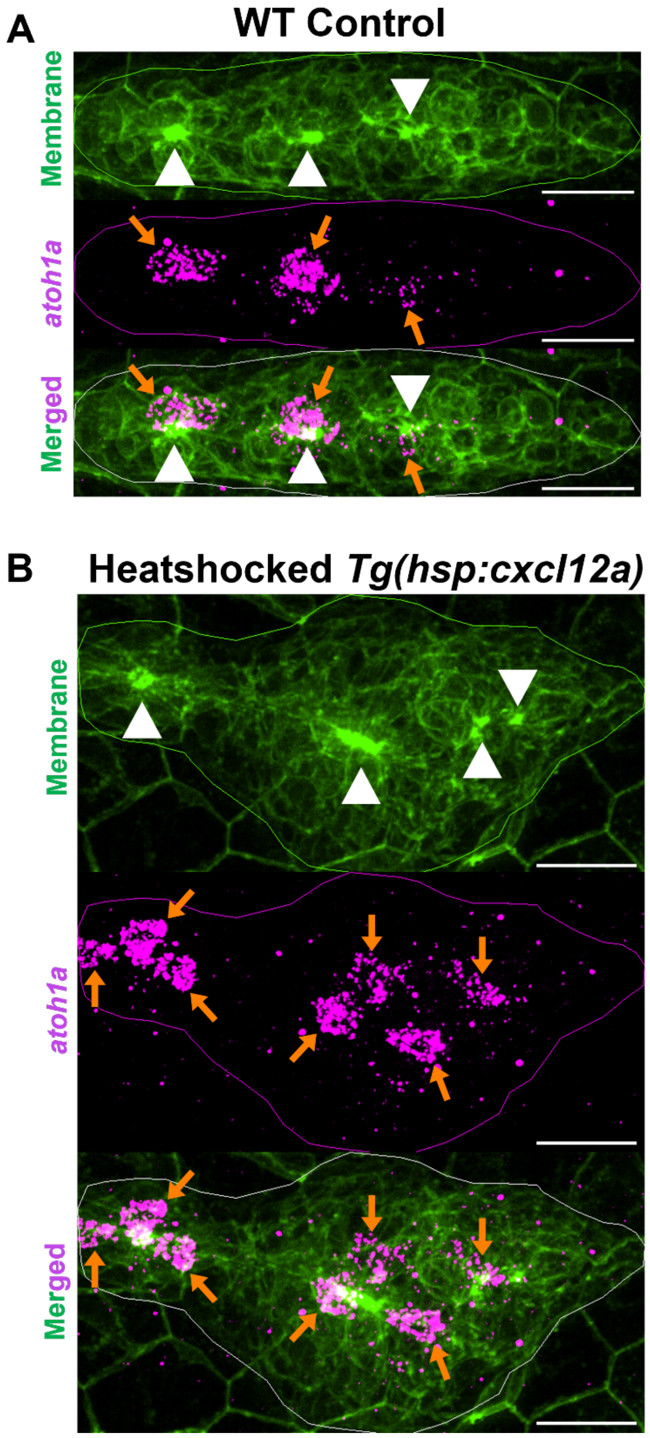

Fig. S4. (A) A representative example of a WT Control PLLp showing the one-to-one association of individual constrictions (white arrowheads) with discrete *atoh1a* expression (orange arrows). **(B)** A representative example of a WT Control PLLp showing the association of individual constrictions (white arrowheads) with multiple discrete *atoh1a* expression areas (orange arrows).

Development • Supplementary information

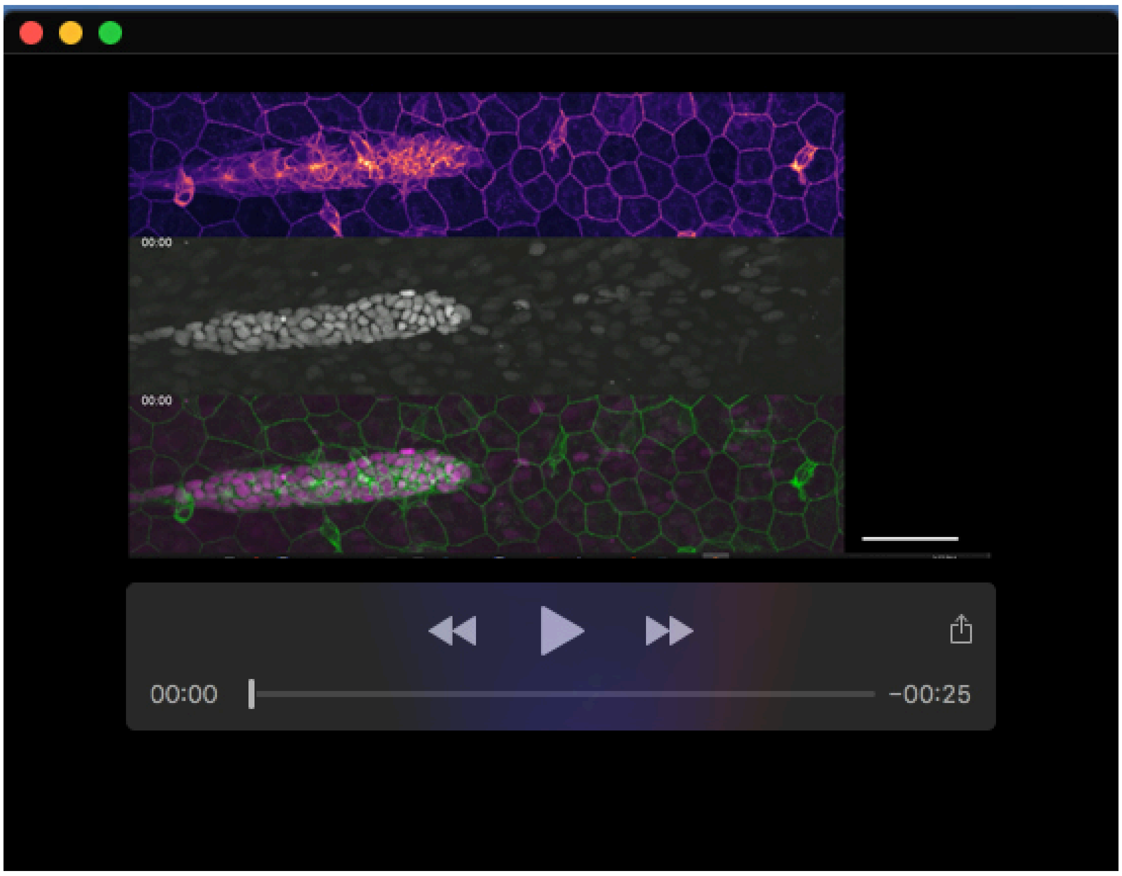

Movie 1. A migrating wildtype control PLLp showing cell membranes (top, high intensity = warm hues), nuclei (middle, gray), and merged (bottom, green = membranes, magenta = nuclei) images.

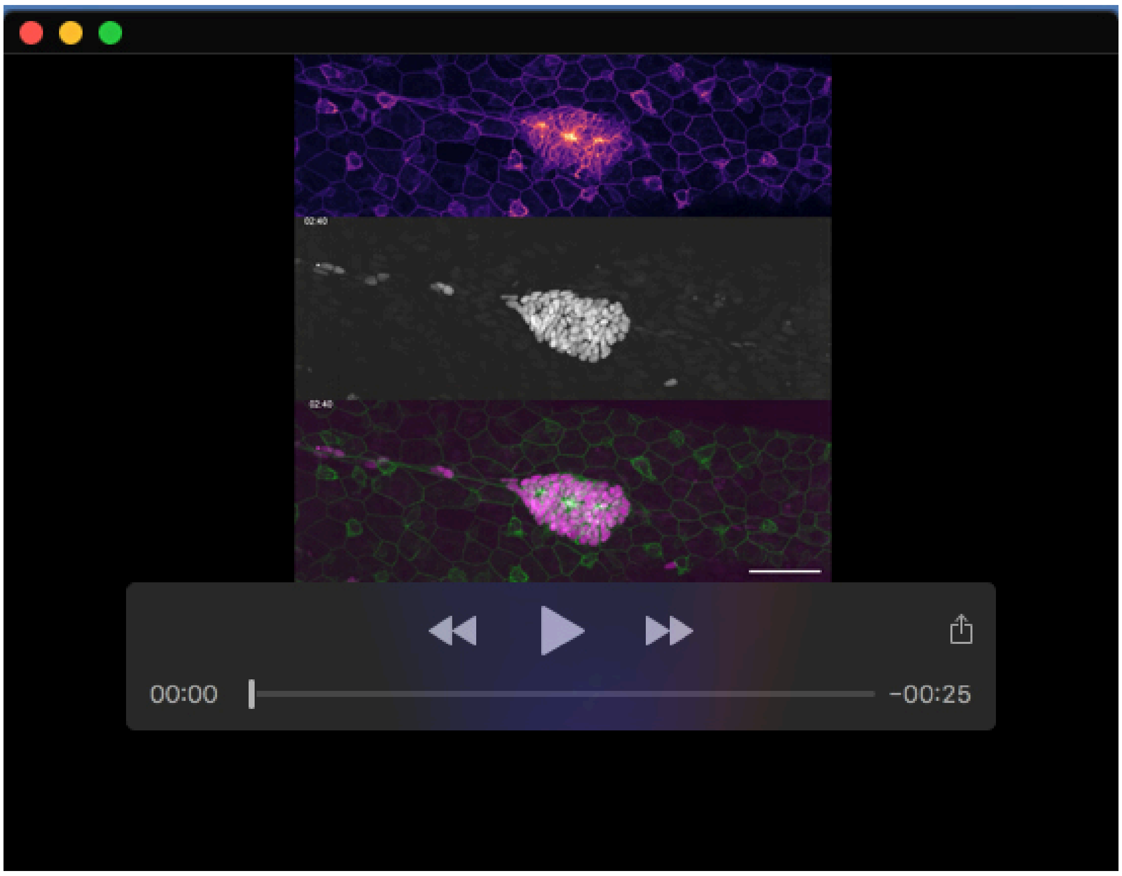

Movie 2. A migrating PLLp in a heat-shocked *Tg(hsp:cxcl12a)* embryo showing cell membranes (top, high intensity = warm hues), nuclei (middle, gray), and merged (bottom, green = membranes, magenta = nuclei) images.

Development • Supplementary information

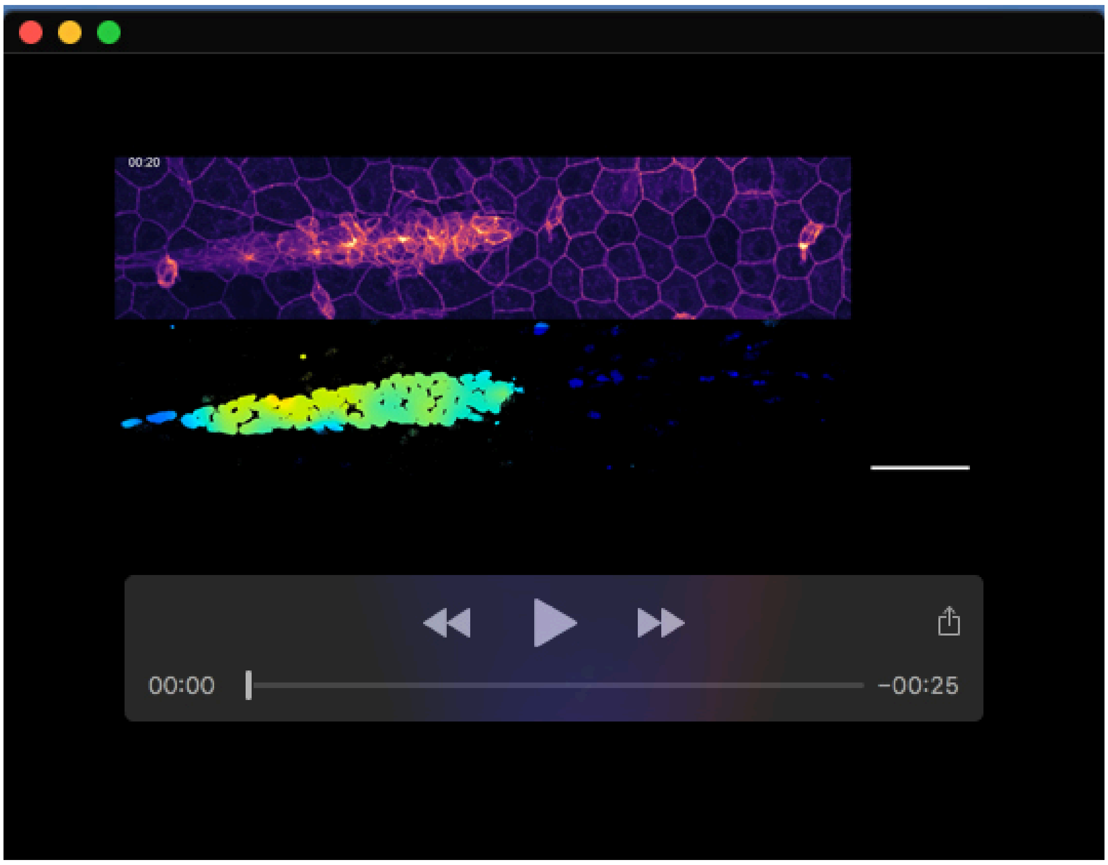

Movie 3. The migrating wildtype control PLLp from Movie 1, showing cell membranes (top, high intensity = warm hues) and the corresponding PIV maps overlaid on cell nuclei (bottom, high migration velocity = warm hues).

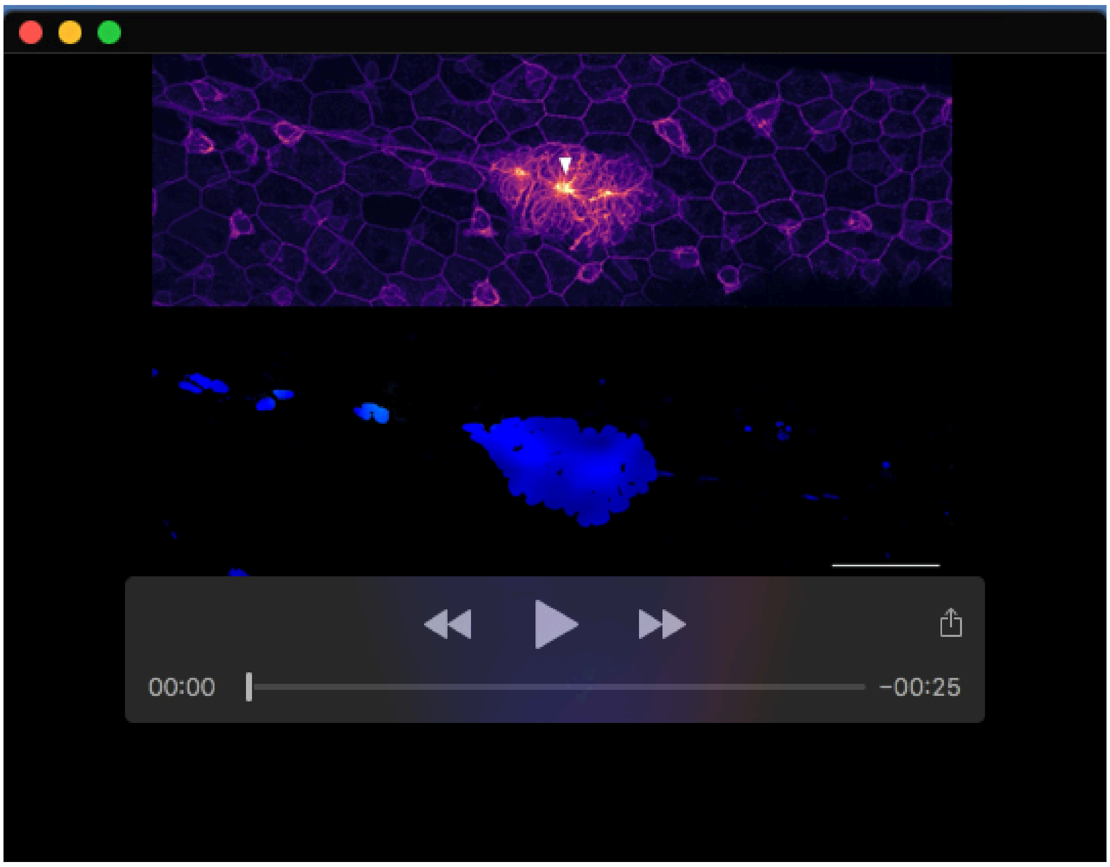

Movie 4. The migrating PLLp in the heat-shocked *Tg(hsp:sdf1a)* embryo from Movie 2, showing cell membranes (top, high intensity = warm hues) and the corresponding PIV maps overlaid on cell nuclei (bottom, high migration velocity = warm hues). White arrowheads mark fusing constrictions.

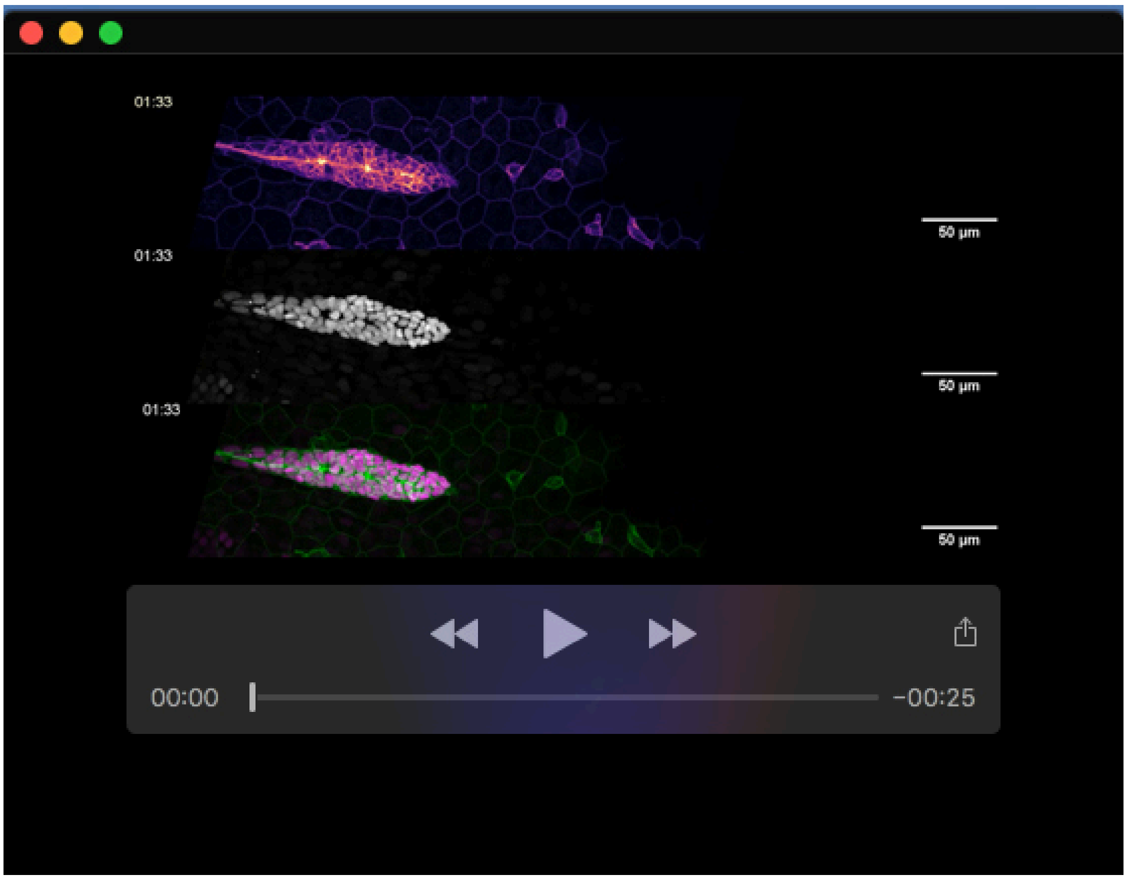

Movie 5. A migrating PLLp in a 2ng *cxcl12a* morpholino-injected embryo showing cell membranes (top, high intensity = warm hues), nuclei (middle, gray), and merged (bottom, green = membranes, magenta = nuclei) images.

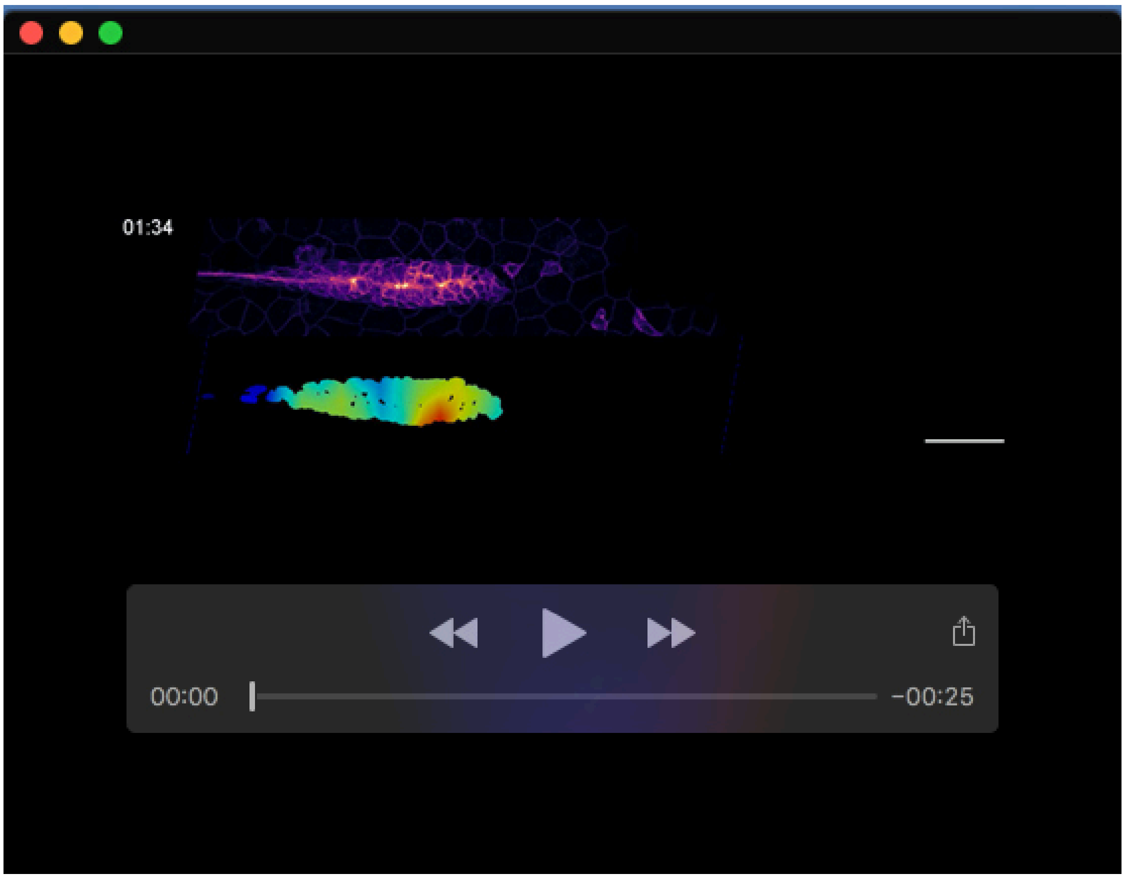

Movie 6. The migrating PLLp from Movie 5, shown with cell membranes (top, high intensity = warm hues) and the corresponding PIV maps overlaid on cell nuclei (bottom, high migration velocity = warm hues). White arrowheads mark constrictions that fuse and then split.

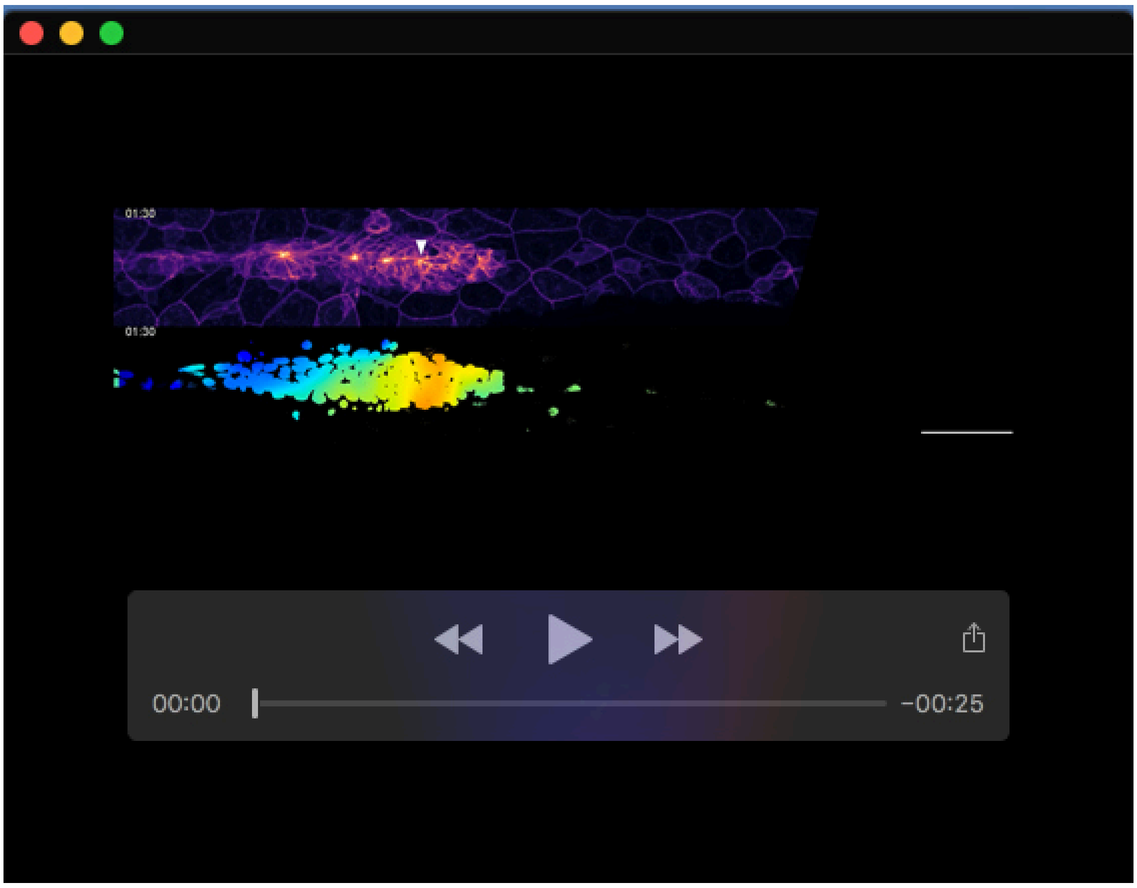

Movie 7. A migrating PLLp treated with 20 μM PP1 showing cell membranes (top, high intensity = warm hues) and the corresponding PIV maps overlaid on cell nuclei (bottom, high migration velocity = warm hues). White arrowheads mark constrictions that split.

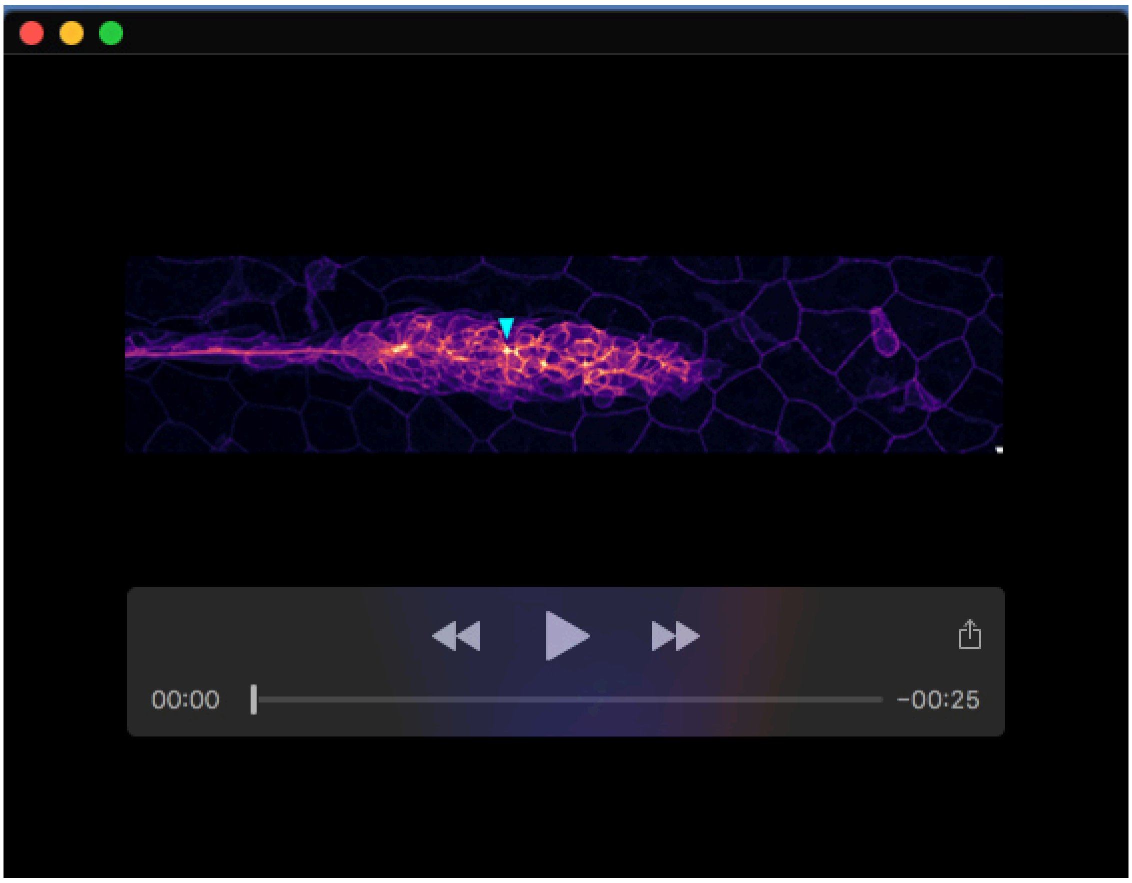

Movie 8. A migrating PLLp in a heat-shocked *Tg(hsp:fgf10)* embryo showing cell membranes (top, high intensity = warm hues). White and cyan arrowheads mark the first and second constrictions that split.

Development • Supplementary information

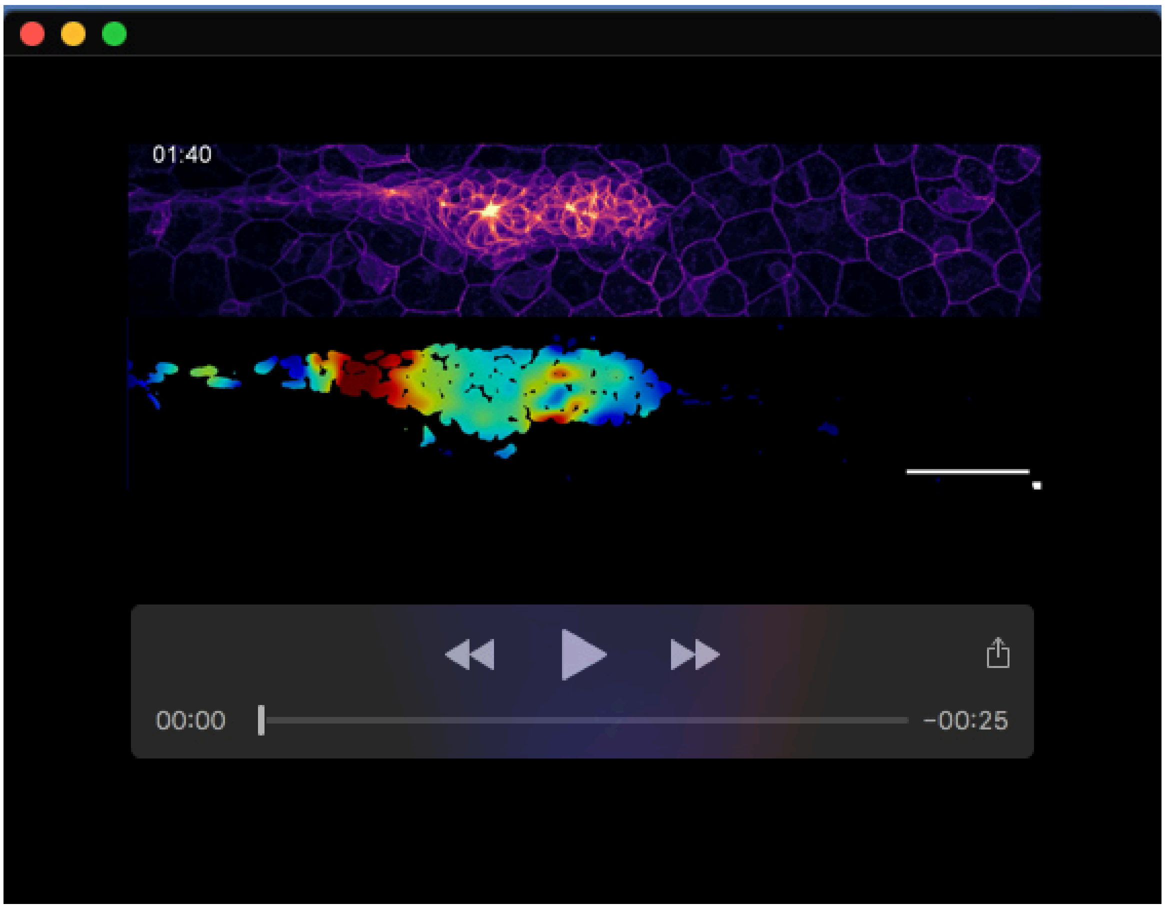

Movie 9. A migrating PLLp in a *Tg(hsp:fgf10)* embryo treated with 20 µM PP1 for 2 hours before heatshocking. Cell membranes (top, high intensity = warm hues) and the corresponding PIV maps overlaid on cell nuclei (bottom, high migration velocity = warm hues). White arrowheads mark constrictions that split and then re-fuse.

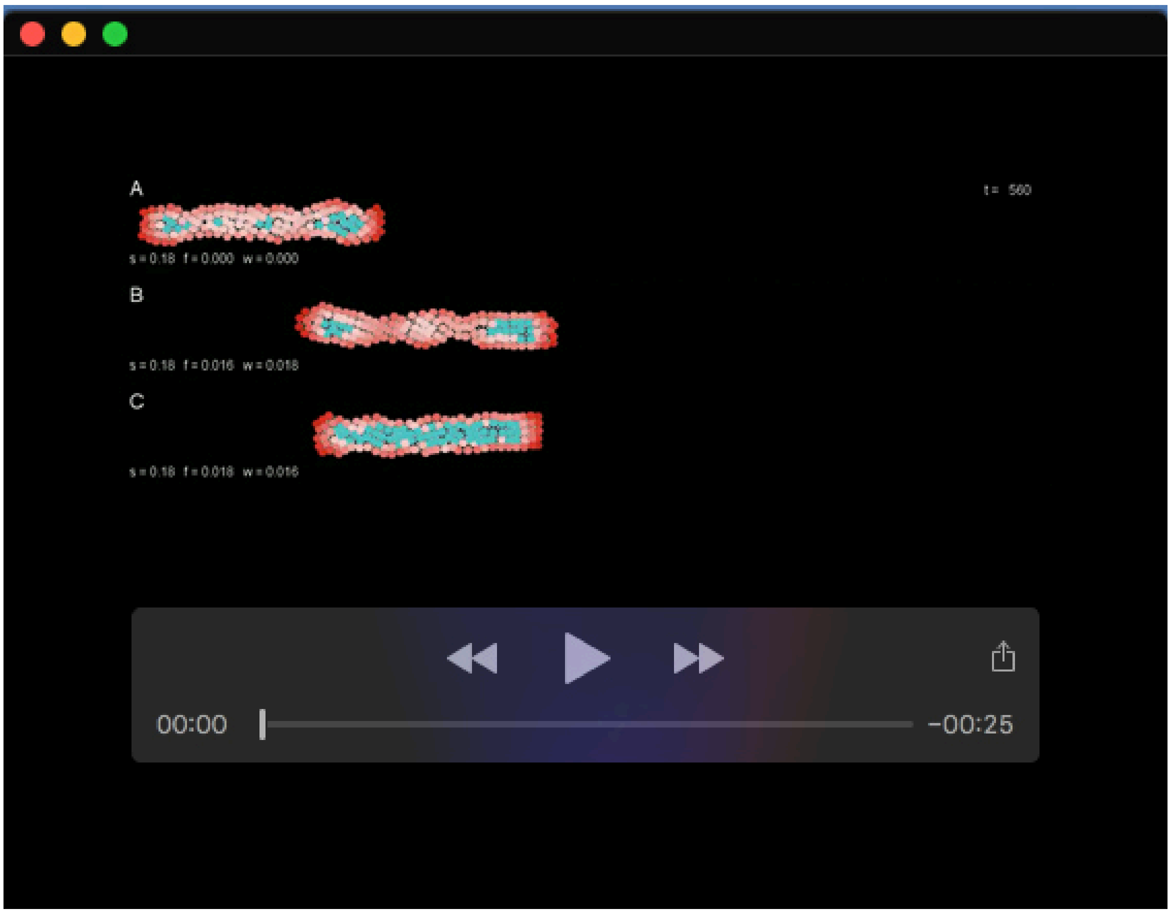

Movie 10. A representative set of agent-based model simulations. Simulations showing aggregation of turtles (agents) without migration (Top), with **WNTers** moving faster than **FGFers** (middle), and with **WNTers** moving slower than **FGFers** (bottom).

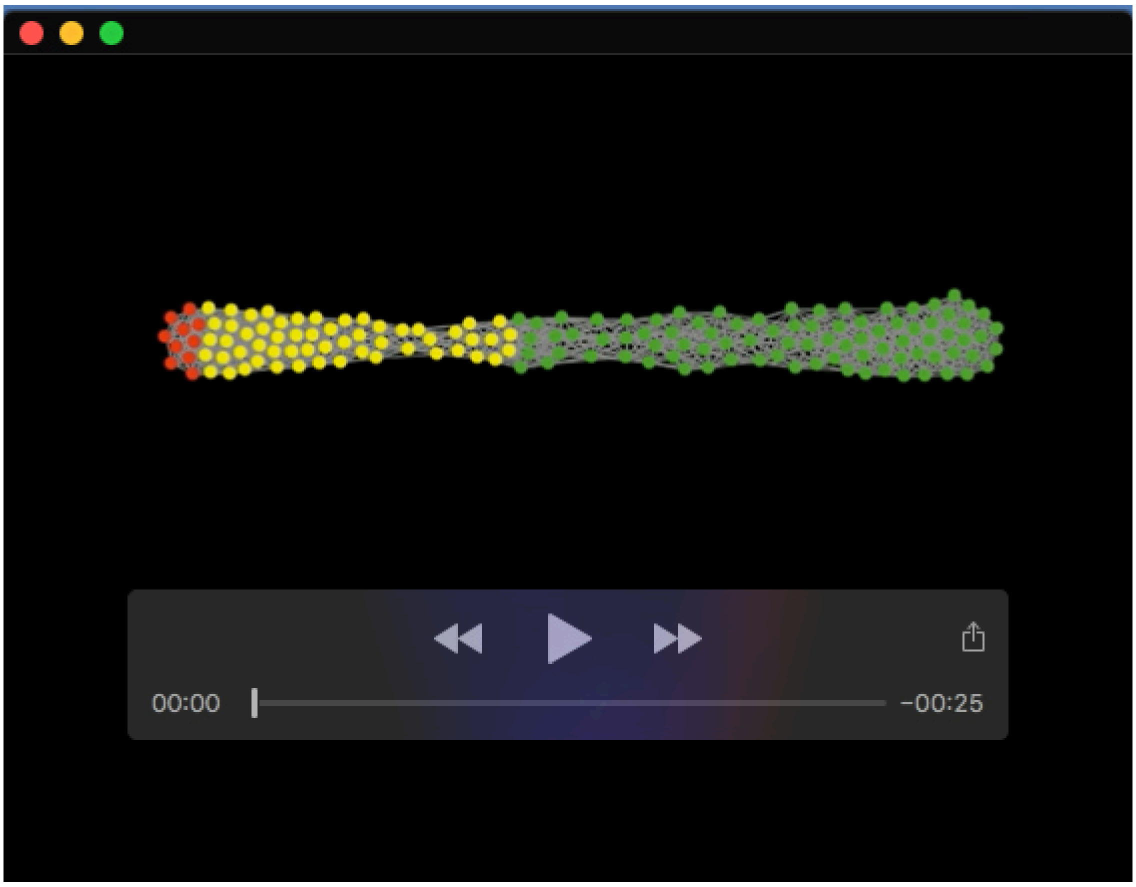

Movie 11. A representative agent-based model simulation showing the shrinkage of Wnt zone (green **WNTers**), and consequent re-specification of FGF zone (yellow **FGFers**) and deposition of red **Depositers**.

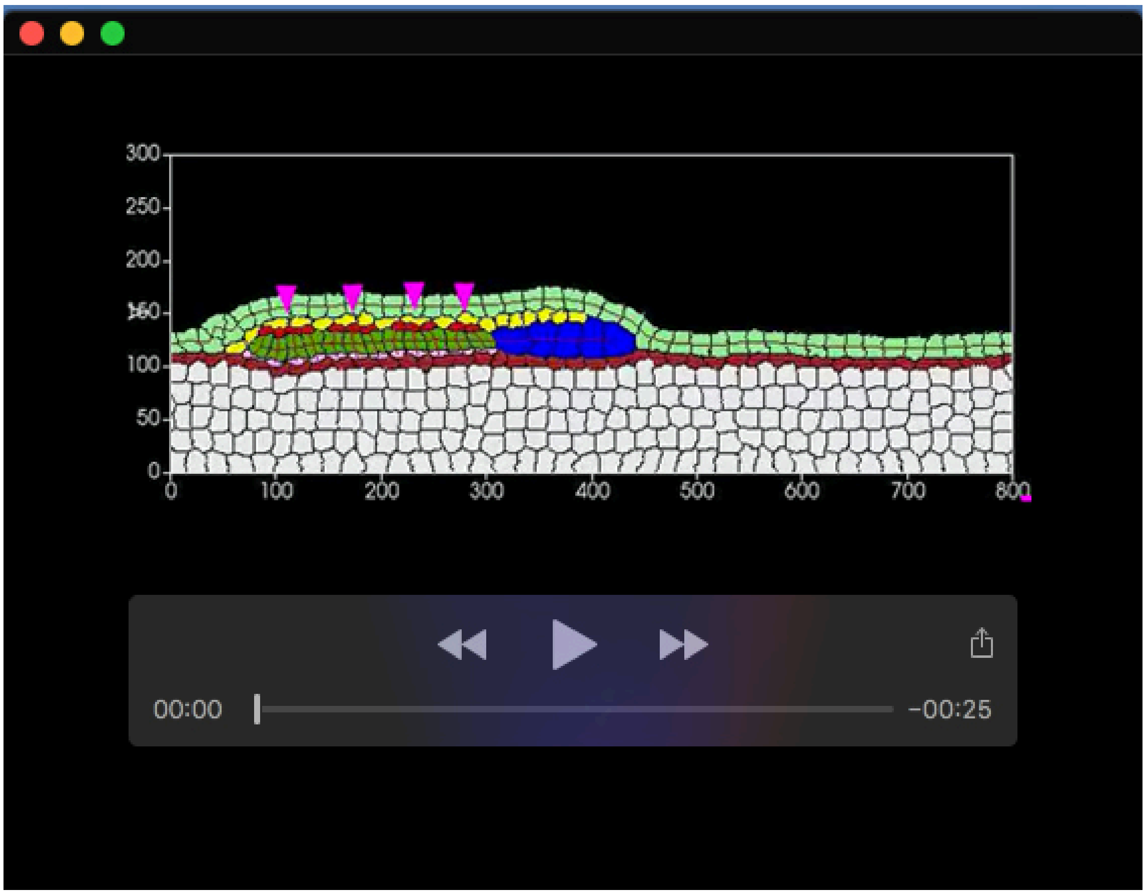

Movie 12. A representative Cellular Potts model PLLp simulating wild-type control conditions. Magenta arrowheads indicate rosettes.

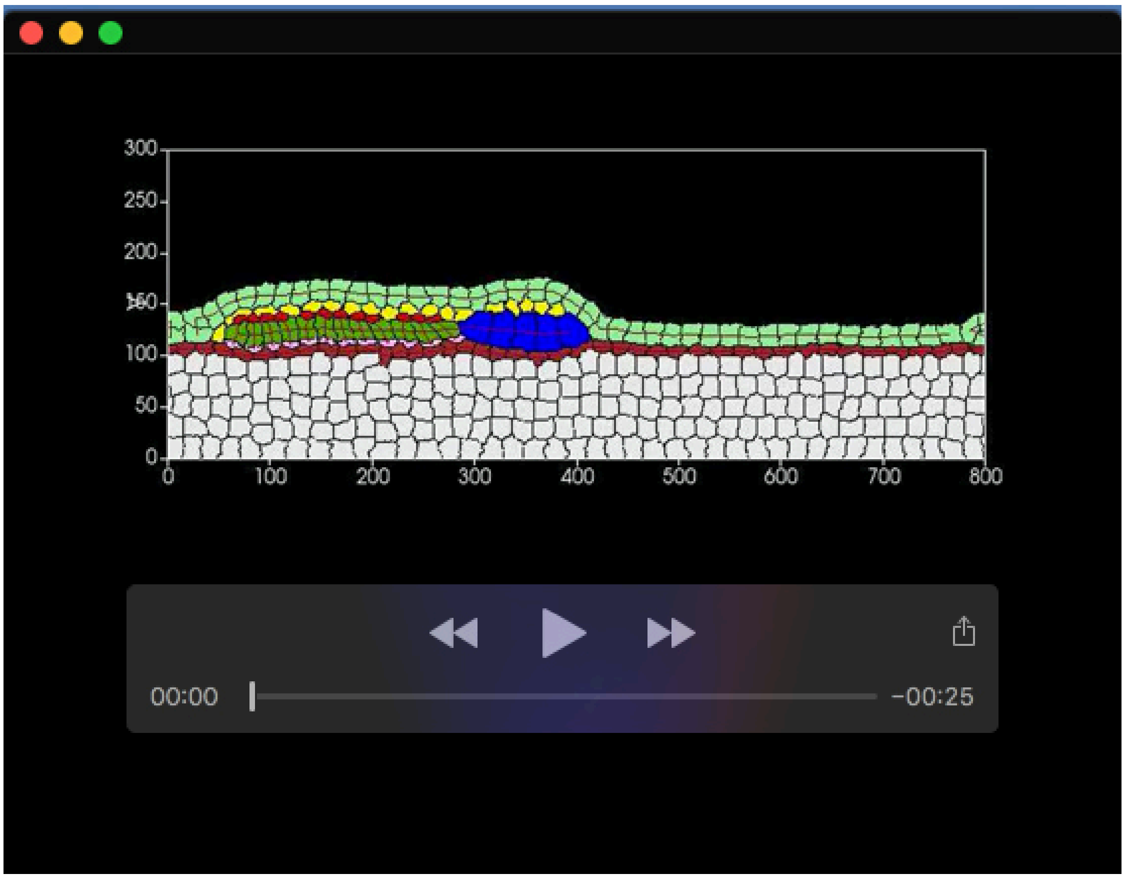

Movie 13. A representative Cellular Potts model PLLp where leading cells slow down before resuming migration, thereby simulating the heat-shocked *Tg(hsp:cxcl12a)* phenotype. Magenta arrowheads indicate rosettes that fuse when the leading domain stalls and then split after migration resumes. Black arrow indicates approximate location of leading cell tip when the blue **Wnt** cells start slowing.

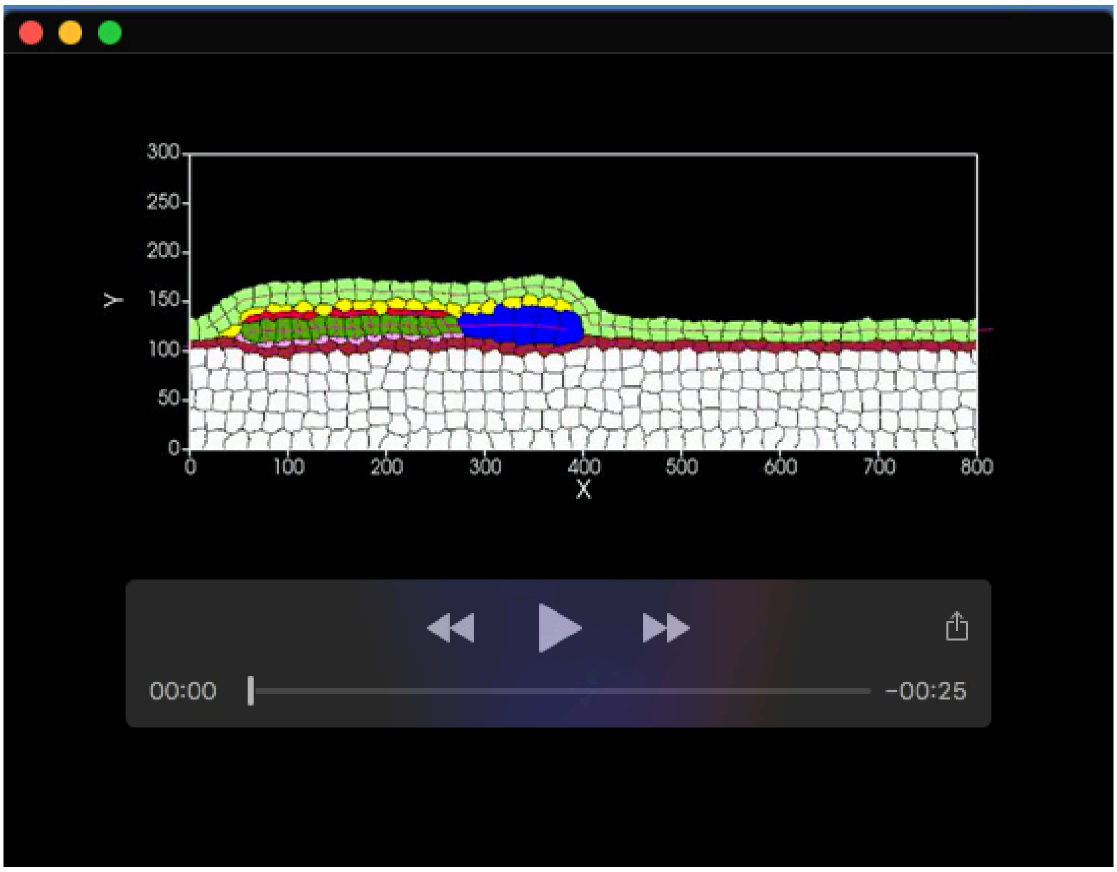

Movie 14. A representative Cellular Potts model PLLp where trailing cells slow down before resuming migration, thereby simulating the 20 μM PP1 treatment phenotype. Magenta arrowheads indicate rosettes that split. Black arrow indicates approximate location of basal domain of the trailing cell when the **FGF** cells start slowing.

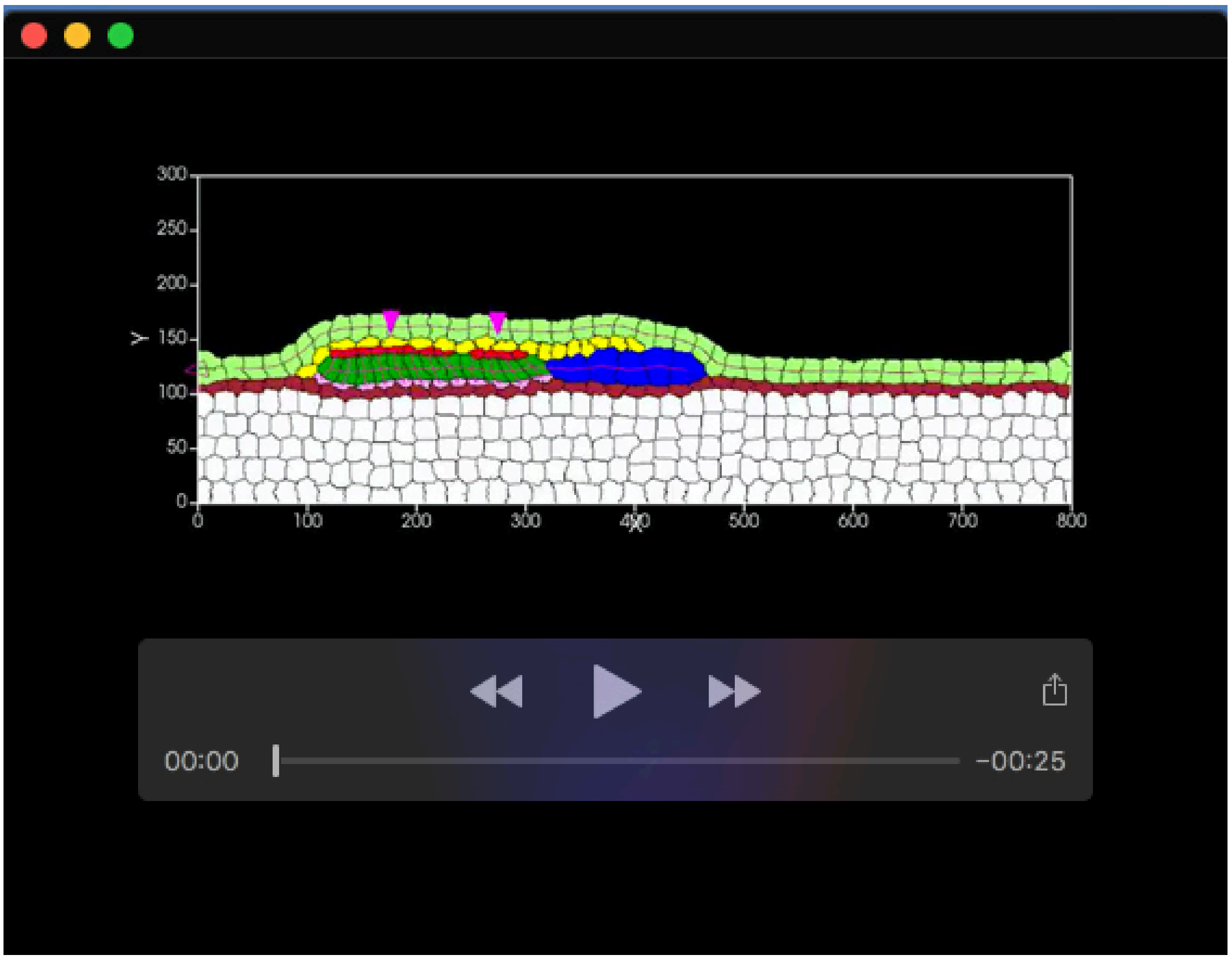

Movie 15. A representative Cellular Potts model PLLp where an increase in **FGF** lateral domain (**Fgflat**) contractility and adhesion leads to formation of a giant rosette. Magenta arrowheads indicate rosettes.

Development • Supplementary information
