# Supplementary material for: Mechanical interactions dynamically influence rosette morphogenesis in the migrating zebrafish posterior lateral line primordium
Source: Development. 2026 Jul 15;153(13):dev204973. doi: 10.1242/dev.204973 (PMC13405218; doi:10.1242/dev.204973)
Supplement: Supplementary file 1 [file develop-153-204973_review_history.pdf]

Mechanical interactions dynamically influence rosette morphogenesis in the migrating zebrafish posterior lateral line primordium

Abhishek Mukherjee, Michael Hilzendeger, Arin Rinvelt, Sana Fatma, Megan Schupp, Damian Dalle Nogare and Ajay B. Chitnis

DOI: 10.1242/dev.204973

Editor: Steve Wilson

Review timeline

Original submission:	24 May 2025
Editorial decision:	27 June 2025
First revision received:	24 April 2026
Editorial decision:	20 May 2026
Second revision received:	29 May 2026
Accepted:	4 June 2026

Original submission

First decision letter

MS ID#: dev.204973

MS TITLE: Mechanical interactions dynamically influence rosette morphogenesis in the migrating zebrafish Posterior Lateral Line primordium

AUTHORS: Abhishek Mukherjee; Michael Hilzendeger; Arin Rinvelt; Sana Fatma; Megan Schupp; Damian Dalle Nogare; Ajay Balwant Chitnis

Dear Ajay,

I have now received all the referees' reports on the above manuscript, and have reached a decision. The referees' comments are appended below, or you can access them online: please go to:

As you will see, the referees express considerable interest in your work, but have some suggestions for improvements and clarifications. If you are able to revise the manuscript along the lines suggested, I will be happy receive a revised version of the manuscript. Please also note that Development will normally permit only one round of major revision. If it would be helpful, you are welcome to contact us to discuss your revision in greater detail. Please send us a point-by-point response indicating your plans for addressing the referees' comments, and we will look over this and provide further guidance.

Please attend to all of the reviewers' comments and ensure that you clearly highlight all changes made in the revised manuscript. Please avoid using 'Tracked changes' in Word files as these are lost in PDF conversion. I should be grateful if you would also provide a point-by-point response detailing how you have dealt with the points raised by the reviewers in the 'Response to Reviewers' box. If you do not agree with any of their criticisms or suggestions please explain clearly why this is so.

Reviewer 1

© 2026. Published by The Company of Biologists under the terms of the Creative Commons Attribution License (https://creativecommons.org/licenses/by/4.0/).

SUMMARY OF THE ADVANCE MADE IN THIS PAPER AND ITS POTENTIAL SIGNIFICANCE TO THE FIELD

In this manuscript, Mukherjee et al address an interesting and important question of how signaling and mechanics are coordinated to drive the morphogenesis of the zebrafish lateral line primordium. In particular, the authors focus on the process of rosette formation within emerging neuromasts. Using an agent-based and a cellular potts mechanical models, they propose that rosette size is determined by a balance in tension, generated by the migration of the primordium, and homotypic adhesion. Alterations in the migration speed modify tension, leading to changes in the number and size of rosettes. The authors provide some experimental evidence to support their model.

The significance of this work lies in describing the role of mechanics in a system where the biochemical signaling system has been extensively studied. This offers an opportunity to understand the relationship and integration between mechanics and biochemical signals in morphogenesis.

Overall, this is an exciting study, yet a few aspects could be clarified and strengthened prior to publication.

SUGGESTIONS TO AUTHORS

Major points:

1. The simulation and experimental results suggest that initial rosette formation is a distinct phenomenon from the consolidation of rosettes. The authors show that the consolidation process is determined by fusion events that depend on migration speed, as well as adhesion strength (Fig. S2 simulations). Could they provide some characterization of the initial formation as well? Is there a characteristic starting number, position and length scale of the rosettes in simulations? What does it depend on?

2. One of the key assumptions of the model is that cells adhere with higher affinity to cells of their own type. Could the authors provide some experimental evidence to support this assumption?

3. Currently, the simulation results are presented mostly in terms of snapshots from the simulation run, but no comprehensive quantitative characterization of the results is provided. The authors could extract parameters such as rosette size, number, position, fusion events over time for different conditions and plot this to achieve a clearer overview of the results. This would also enable more direct comparison with experimental data.

4. The HS:sdf1a condition and PP1 treatment in figure 1 are interpreted as compression and stretching of the primordium due to the changing speed. Can the authors provide some quantification of the compression/ stretch factor in these conditions? How does that compare to simulations?

5. The model parameters are listed in the Supp tables but no rationale has been provided for how they are selected. What is the model robustness for parameter variation? How do specific behaviors depend on the choice of parameters?

Minor:

6. Can you please provide some units for the parameters listed in Supp table 1. At the very least it should be clear whether the listed parameters are specified in comparable units (e.g. are time units the same between different parameters?).

7. In Fig. 2 - it is a bit difficult to understand how it was defined that atoh1 is expressed in one or more cells. Please provide methodological details of the way the assignment of phenotypes was performed.

8. The adhesion parameters to ECM and skin are set differently for Wnt and Fgf cells (Table 3). What is the rationale behind this? How does it influence the behavior of the model?

© 2026. Published by The Company of Biologists under the terms of the Creative Commons Attribution License (https://creativecommons.org/licenses/by/4.0/).

9. The simulation scenario in which there is no migration at all is a nice illustration of the idea that initial rosette formation is independent of polarized cues and migration. Could the authors support this experimentally by analyzing a condition in which migration is blocked from early times?

10. Fig. 1 - when was the heatshock performed? Please clarify in fig legend and methods.

11. Fig. S2 - provide information about the other parameters in the fig legend - is there migration in this case?

12. Fig. 3B and C- the images do not convincingly convey the stated relationship between cell density and color - the cyan colored cells seem to sometimes be more loosely packed and somewhat comparable to some of the pink cells. Is this coloring done automatically? How exactly is the density of neighbors defined? What are the asterisks in 3C? Please clarify.

13. Line 329 - reference is missing

Reviewer 2

SUMMARY OF THE ADVANCE MADE IN THIS PAPER AND ITS POTENTIAL SIGNIFICANCE TO THE FIELD

This manuscript investigates the dynamics of epithelial rosette formation in the zebrafish posterior lateral line primordium (pLLP), revealing that rosettes are initially unstable and can undergo fusion or splitting. The authors demonstrate that slowing the migration of leading or trailing cells influence rosette behavior, promoting either fusion or splitting, respectively, suggesting that rosette number and size are influenced by migration-dependent mechanical forces. The study also uncovers a potential decoupling between apical constriction and atoh1a expression in sensory hair cell progenitors (SHCPs), suggesting initially independent mechanisms that converge over time. To support these findings, the authors develop two computational models to test the influence of mechanical tension along the migration axis on rosette morphogenesis. Overall, the study proposes that mechanical tension works alongside FGF-mediated signaling to shape collective cell behavior during pLLP migration.

The manuscript addresses a biologically interesting and complex question using a combination of live imaging, genetic perturbation, and modeling. The integration of computational simulations is a strength. However, several aspects of the experimental design (in particular regarding the sample size and reproducibility) and interpretation require clarification or additional experiments to support the main conclusions.

SUGGESTIONS TO AUTHORS

Major comments:

1. Interpretation of manipulations in Cxcl12a signaling (Figure 1):
* The section title ("Manipulation of Cxcl12a chemokine signaling slows leading cells in the PLLp") does not accurately reflect the novel findings. The fact that Cxcl12a manipulations affect leading cell behavior is well established. Here, the key observation is that this slowing leads to rosette fusion. The title should be revised accordingly.
* For all three manipulations (hs:sdf1, sdf1 morpholino, and PP1 treatment), quantitative data on experimental reproducibility is lacking. How many independent experiments were performed? In how many embryos was rosette fusion or splitting observed? These "n" values should be clearly indicated in the figure legends or methods.
* The inclusion of a time-lapse movie illustrating rosette splitting in PP1-treated embryos (Figure 1C) would strengthen this claim.
* To support the conclusion that slowing trailing cells induces rosette splitting, an independent perturbation strategy should be tested. For example, use of low-dose cxcr7 morpholino could provide additional validation that reduced rear cell motility induces splitting of rosettes.

© 2026. Published by The Company of Biologists under the terms of the Creative Commons Attribution License (https://creativecommons.org/licenses/by/4.0/).

* The proposed model implies that changes in mechanical tension along the anteroposterior axis influence rosette behavior. Could this tension be directly assessed (e.g., via laser ablation or other biomechanical measurements)? Even a discussion of this limitation would be valuable.
* It would also be informative to test whether dual perturbation (e.g., low-dose cxcl12 morpholino + PP1) could rescue the splitting phenotype by restoring balanced tension. This would help support the tension-based model.

2. Coupling between atoh1a expression and apical constriction (Figure 2):
* The authors report that atoh1a-positive cells can exceed the number of apical constrictions following cxcl12 overexpression, consistent with rosette fusion. Given that Notch signaling limits SHCP number via lateral inhibition, one would expect this initial excess to resolve over time. It would be informative to examine whether SHCP numbers normalize at later stages.
* cxcr4b mutants exhibit impaired migration and likely undergo rosette fusion. Quantifying atoh1a-positive cells per rosette in these mutants could provide additional support for the model.
* Similarly, does the sdf1 morpholino induce a comparable excess in atoh1a-positive cells, consistent with fusion events?
* The authors state that apical constrictions are not always associated with a single, well-defined atoh1a-expressing cell. However, clustered expression of atoh1a in newly forming rosettes has been described previously (e.g., Lecaudey et al., 2008; Nechiporuk et al., 2008; Matsuda et al., 2010). The current phrasing may mislead readers into thinking this is a novel observation. The authors should clarify this and appropriately cite prior literature.

3. Inhibition of Src signaling (PP1 treatment):
* This section is brief and complement the initial results on cxcl12a perturbation. Consider integrating it into the first section for clarity and flow.
* The authors should examine atoh1a expression in PP1-treated embryos: does a new atoh1a-expressing cell appear in the split rosettes? This would clarify whether rosette splitting leads to re-specification of SHCPs.
* See additional comments above regarding reproducibility and possible rescue experiments.

4. Modeling approaches and interpretation (Figures 3 & 5):
* The authors describe the Wnt-active domain as encompassing the leading 60% of the primordium. This is inconsistent with previous studies (e.g., Aman & Piotrowski, 2008; studies from the Chitnis lab), which place Wnt activity in the leading third and FGF signaling in the trailing two-thirds. This discrepancy should be acknowledged and justified.
* The model shown in Figure 3 simulates cell aggregation rather than apical constriction. As the authors acknowledge, proximity does not necessarily correlate with rosette organization (as evidenced by nuclear positioning). The implications of this model should be clarified: what does it capture, and what are its limitations?
* In the Cellular Potts Model (Figure 5), it would be informative to simulate slower migration of trailing cells (to mimic PP1 treatment) and examine whether this leads to rosette splitting. This would strengthen the link between experimental observations and model behavior.
* The rationale for associating increased contractility and adhesion in the model with Notch overactivation is unclear. The authors should elaborate on why this parameterization reflects Notch gain-of-function and how it relates to in vivo data.

Additional minor comments:

* Line 76-77: "Posterior Lateral Line" should be in lowercase.
* Line 78: "follow" should be "flow".
* Line 854: "µm" should be written as "µM".
* Line 904: "apical (pink)" should be "apical (red)".

Conclusion:

This manuscript presents evidence that mechanical forces influence the spatial organization of epithelial rosettes in the migrating lateral line primordium. The experimental framework and modeling are valuable, but key aspects require further quantification, clarification, and, where feasible, additional validation. With revision, this work has the potential to make a significant

© 2026. Published by The Company of Biologists under the terms of the Creative Commons Attribution License (https://creativecommons.org/licenses/by/4.0/).

contribution to our understanding of how signaling and mechanics interact during collective epithelial morphogenesis.

Reviewer 3

SUMMARY OF THE ADVANCE MADE IN THIS PAPER AND ITS POTENTIAL SIGNIFICANCE TO THE FIELD

Mukherjee et al describe experiments to test a model where interactions between migration and cellular interactions combine to form rosettes in the migrating lateral line primordium. They demonstrate how manipulation of migration rates of leading and trailing regions of the moving primordium result in changes in apical constrictions and altered rosette patterning. Assessing gene expression demonstrates a decoupling of rosette formation and atoh1a expression. Using agent-based models, the authors demonstrate how changes in migration and cellular interactions is sufficient to predict observed patterning differences. Using Cellular Potts models they model how apical constriction and migration contribute emergent rosette formation as migration proceeds. Overall the combination of embryonic manipulation and computational modeling demosntrate how mechanical coupling combined with changes in migration can influence dynamic patterning. Accompanying movies contribute to provide the overall picture.

SUGGESTIONS TO AUTHORS

The authors manipulate cxcl12a by heatshock overexpression or morpholino knockdown to slow leading cells compared to trailing cells. The authors also describe that treatment with PP1 results in slowing trailing cells compared to leading cells. These manipulations in turn affect the number of rosettes. While the examples in Figure 1 and movies are clear, it would be good to have a quantitative assessment to describe the variation in rates of movement and numbers of rosettes. Does changes in migration precede changes in rosette number?

In figure 2, it would be nice if labels were included for the color-coded images to make it easier to understand.

In figure 5 I found it difficult to make out the individual cells - I wonder whether a close-up of a group of cells could be included as an inset perhaps in the black space of each panel.

First revision

Author response to reviewers' comments

Dear Editor,

We are pleased to resubmit our paper “Signaling and Mechanics influence the number and size of epithelial rosettes in the migrating zebrafish Posterior Lateral Line primordium” to Development. We thank you for the comments of the reviewers. We have responded to their suggestions with several additional experiments, analyses and changes to the text, which we believe significantly improve the paper. We hope that the reviewers will find that these changes now make the paper suitable for publication. We would also like to thank the editors for their generosity in giving us adequate time for making these changes. A detailed response to reviewers’ comments is provided below. Given the focus of the paper on the role of dynamic mechanical interactions in the morphogenesis of epithelial rosettes, we also suggest an alternate title that we feel better describes the paper, “Mechanical interactions dynamically influence rosette morphogenesis in the migrating zebrafish Posterior Lateral Line primordium”

On behalf of the authors, Abhishek Mukherjee and Ajay Chitnis

Reviewer 1:

© 2026. Published by The Company of Biologists under the terms of the Creative Commons Attribution License (https://creativecommons.org/licenses/by/4.0/).

SUMMARY OF THE ADVANCE MADE IN THIS PAPER AND ITS POTENTIAL SIGNIFICANCE TO THE FIELD:

In this manuscript, Mukherjee et al address an interesting and important question of how signaling and mechanics are coordinated to drive the morphogenesis of the zebrafish lateral line primordium. In particular, the authors focus on the process of rosette formation within emerging neuromasts. Using an agent-based and a cellular potts mechanical models, they propose that rosette size is determined by a balance in tension, generated by the migration of the primordium, and homotypic adhesion. Alterations in the migration speed modify tension, leading to changes in the number and size of rosettes. The authors provide some experimental evidence to support their model.

The significance of this work lies in describing the role of mechanics in a system where the biochemical signaling system has been extensively studied. This offers an opportunity to understand the relationship and integration between mechanics and biochemical signals in morphogenesis.

Overall, this is an exciting study, yet a few aspects could be clarified and strengthened prior to publication.

We thank Reviewer 1 for their comments

SUGGESTIONS TO AUTHORS:

Major points:

1. The simulation and experimental results suggest that initial rosette formation is a distinct phenomenon from the consolidation of rosettes. The authors show that the consolidation process is determined by fusion events that depend on migration speed, as well as adhesion strength (Fig. S2 simulations). Could they provide some characterization of the initial formation as well? Is there a characteristic starting number, position and length scale of the rosettes in simulations? What does it depend on?

Response - We have now added graphs to Fig. 3 and Fig. S2 showing how the primordium length, number of clusters, and number of cells per cluster changes in the three conditions as a function of time, a) when there is no active movement of cells, b) when the leading Wnters are slightly faster than the trailing FGFers (w= 0.018, f= 0.016), and c) when leading Wnters are slower than FGFers (w=0.016, f=0.018). Asterisks show cell clusters. In general, a faster leading domain results in greater stretch and fewer clusters. Greater spring strength results in shorter length, fewer clusters and more cells in each cluster.

2. One of the key assumptions of the model is that cells adhere with higher affinity to cells of their own type. Could the authors provide some experimental evidence to support this assumption?

Response - We had no direct experimental evidence for this assumption or for any of the parameters defining differential adhesion between different cells. It was simply based on our observation from in toto imaging of the primordium that there was minimal mixing of cells in the primordium. We further hypothesized that to minimize mixing, cells of the same type would be most adherent to each other. However, as described in the much-expanded section on how contact energies were specified in MATERIALS and METHODS, this rule was not always followed and additional considerations, described the new section, detail the reasoning behind some of the initial assumptions made to specify contact energies associated with different pairs of cells or cell compartments. Eventually these initial parameters were tweaked till we had the relevant emergent behavior of the primordium described in this study.

3. Currently, the simulation results are presented mostly in terms of snapshots from the simulation run, but no comprehensive quantitative characterization of the results is provided. The authors could extract parameters such as rosette size, number, position, fusion events over time for different conditions and plot this to achieve a clearer overview of the results. This would also enable more direct comparison with experimental data.
Response - Graphs have been added to figures 2, 3 and S2 to capture some of these dynamics. The simplicity of the models compared to complexity of the system in vivo makes direct comparison

© 2026. Published by The Company of Biologists under the terms of the Creative Commons Attribution License (https://creativecommons.org/licenses/by/4.0/).

between some details of the results in the two situations difficult and potentially misleading. We used the model simulation to illustrate general concepts rather than make direct quantitative comparisons with the in vivo observations.

4. The HS:sdf1a condition and PP1 treatment in figure 1 are interpreted as compression and stretching of the primordium due to the changing speed. Can the authors provide some quantification of the compression/ stretch factor in these conditions? How does that compare to simulations?
Response - We have now included graphs that show compression or stretching that preceded fusion or splitting events in Figures 2D-F and S1.

5. The model parameters are listed in the Supp tables but no rationale has been provided for how they are selected. What is the model robustness for parameter variation? How do specific behaviors depend on the choice of parameters?
Response - We have expanded the section in MATERIALS and METHODS to elaborate on how model parameters were specified. Though sensitivity to the parameters was not systematically examined, the sensitivity to specific parameters became evident during the development of the model and some of those details have now been added to the results section.

Minor:
6. Can you please provide some units for the parameters listed in Supp table 1. At the very least it should be clear whether the listed parameters are specified in comparable units (e.g. are time units the same between different parameters?).
Response -Units have been added (patches or pixels for length scale and monte carlo steps (mcs) or ticks for time).

7. In Fig. 2 - it is a bit difficult to understand how it was defined that atoh1 is expressed in one or more cells. Please provide methodological details of the way the assignment of phenotypes was performed.
Response - The entire section using atoh1a cells has been removed from the results section and the reasons have now been elaborated on in the discussion section. As we examined the number and distribution of atoh1a in over 100 primordia we came to appreciate that variability in the pattern number and distribution of atoh1a cells is more than we had appreciated, even in control embryos. As a result, it was difficult to use them as simple proxies for the number and position of Fgf signaling centers that normally initiate rosette formation. Nevertheless, some more obvious examples have been included in the supplementary Figure S5.

8. The adhesion parameters to ECM and skin are set differently for Wnt and Fgf cells (Table 3). What is the rationale behind this? How does it influence the behavior of the model?
Response - This is now described in more detail in the MATERIALS and METHODS section. The lower contact energy associated with Fgf cell basal compartments to the ECM (Fgfbas-ECM = 2) , compared higher contact energy associated with Wnt to the ECM (Wnt-ECM= 5) was introduced in the model to increase drag on trailing cells and potentially increase stretch of the primordium. This feature could be related to the more recent observation that there are stronger traction forces associated with interactions of trailing cells and the underlying ECM reported in Yamaguchi et al 2022, but this was not the original motivation for this feature. Furthermore, though these parameters were used in the version of the model used for most simulations in this study, this difference is not a critical feature.

9. The simulation scenario in which there is no migration at all is a nice illustration of the idea that initial rosette formation is independent of polarized cues and migration. Could the authors support this experimentally by analyzing a condition in which migration is blocked from early times?
Response - Inhibiting initial migration delays polarization of Wnt activity, which remains broad and there is a delay in initiation of Fgf signaling (Neelathi et al 2018), which is critical for initiating changes that promote epithelialization and rosette formation. So simply stalling early migration cannot easily allow us to do what would otherwise be an attractive experiment. We have not found an experimental condition in which we could easily demonstrate rosette formation in a stationary primordium independent of the formation of the prepattern Fgf signaling that normally plays a primary role in determining where the protoneuromasts form. The complexity of stalling the primordium, erasing the prepattern of FGF signaling and visualizing spontaneous rosette formation

© 2026. Published by The Company of Biologists under the terms of the Creative Commons Attribution License (https://creativecommons.org/licenses/by/4.0/).

independent of reforming Fgf signaling centers, is difficult. It could be done in principle with inducible controlled Rho activity and contractility in a stalled primordium. We were unable to include such an experiment in this revision. Though this is not an example in which migration is absent, the experiment in Kozlovskaja-Gumbriene 2017, where Notch activity is induced broadly in the primordium and 2-3 rosettes form in the absence of an Fgf signaling prepattern, illustrates how such rosettes have the potential to self-organize in the absence of a prepattern and how changes in mechanical features affects their number and size.

10. Fig. 1 - when was the heatshock performed? Please clarify in fig legend and methods.
Response - Heat shock was performed when the embryos reached ~27-28 h.p.f. All embryos used in this study were in this range. We have now added this information in the Fig.1 legend and the section on Materials and Methods.

11. Fig. S2 - provide information about the other parameters in the fig legend - is there migration in this case?
Response - Migration parameter was not added to these simulations. This has now been added to the Fig. S2 legend.

12. Fig. 3B and C- the images do not convincingly convey the stated relationship between cell density and color - the cyan colored cells seem to sometimes be more loosely packed and somewhat comparable to some of the pink cells. Is this coloring done automatically? How exactly is the density of neighbors defined? What are the asterisks in 3C? Please clarify.
Response - The cellular agents in Figure 3 are point structures. Cells are represented as hexagons for visualization purposes. Cell neighbor density within a radius of 3 patches is computed automatically for each cell at every timestep (tick). Coloring is set automatically and is dynamic, where darker shades of red indicate low density, while lighter tints of red indicate high density. Cells are colored cyan if density is greater than 32.
Asterisks in Fig. 3C signify aggregation of cells into local clusters, done manually upon visual assessment. Local high-density clusters can be visualized as localized lateral bulges in the column of cells during simulation as they evolve from cell interactions. These clusters can either fuse or remain localized as a result of compression or tension in the system.

13. Line 329 - reference is missing
Response - Dalle Nogare et al 2020 reference has been added

Reviewer 2:

SUMMARY OF THE ADVANCE MADE IN THIS PAPER AND ITS POTENTIAL SIGNIFICANCE TO THE FIELD

This manuscript investigates the dynamics of epithelial rosette formation in the zebrafish posterior lateral line primordium (pLLP), revealing that rosettes are initially unstable and can undergo fusion or splitting. The authors demonstrate that slowing the migration of leading or trailing cells influence rosette behavior, promoting either fusion or splitting, respectively, suggesting that rosette number and size are influenced by migration-dependent mechanical forces. The study also uncovers a potential decoupling between apical constriction and atoh1a expression in sensory hair cell progenitors (SHCPs), suggesting initially independent mechanisms that converge over time. To support these findings, the authors develop two computational models to test the influence of mechanical tension along the migration axis on rosette morphogenesis. Overall, the study proposes that mechanical tension works alongside FGF-mediated signaling to shape collective cell behavior during pLLP migration.
The manuscript addresses a biologically interesting and complex question using a combination of live imaging, genetic perturbation, and modeling. The integration of computational simulations is a strength. However, several aspects of the experimental design (in particular regarding the sample size and reproducibility) and interpretation require clarification or additional experiments to support the main conclusions.

SUGGESTIONS TO AUTHORS

© 2026. Published by The Company of Biologists under the terms of the Creative Commons Attribution License (https://creativecommons.org/licenses/by/4.0/).

Major comments:

1. Interpretation of manipulations in Cxcl12a signaling (Figure 1):
* The section title ("Manipulation of Cxcl12a chemokine signaling slows leading cells in the PLLp") does not accurately reflect the novel findings. The fact that Cxcl12a manipulations affect leading cell behavior is well established. Here, the key observation is that this slowing leads to rosette fusion. The title should be revised accordingly.

Response - We thank the reviewer for this comment. We have now changed the section title to **“Selective slowing of the leading PLLp cells results in fusion of rosettes”**

* For all three manipulations (hs:sdf1, sdf1 morpholino, and PP1 treatment), quantitative data on experimental reproducibility is lacking. How many independent experiments were performed? In how many embryos was rosette fusion or splitting observed? These "n" values should be clearly indicated in the figure legends or methods.
Response The total number of primordia examined and the fraction showing fusion or splitting of constrictions has now been provided in the text.

* The inclusion of a time-lapse movie illustrating rosette splitting in PP1-treated embryos (Figure 1C) would strengthen this claim.
Response - We have included a movie (Movie S7) that illustrates a constriction splitting into two smaller constrictions following exposure to PP1. This experiment is now in Figure 2A. The additional experiments and graphs now included in the paper necessitated splitting the original Figure 1 into two, Figure 1 now illustrates fusion of rosettes flowing selective slowing of leading cells, while Figure 2 describes experiments where slowing of trailing cells resulted in splitting of rosettes.

* To support the conclusion that slowing trailing cells induces rosette splitting, an independent perturbation strategy should be tested. For example, use of low-dose cxcr7 morpholino could provide additional validation that reduced rear cell motility induces splitting of rosettes.
Response - We have now included an additional experiment to selectively slow trailing cells transiently. Just as overexpression of cxcl12a transiently selectively slows leading cells before the entire primordium stalls, we used heat shock to over express Fgf10, the directional cue for trailing cells. As with heat shock induced over-expression of cxcl12a, heat shock induced broad expression of Fgf obfuscates directional information normally provided by leading cells that produce Fgf. This transiently slows trailing cells more than leading cells and this, as with PP1, also results in the splitting of rosettes.

* The proposed model implies that changes in mechanical tension along the anteroposterior axis influence rosette behavior. Could this tension be directly assessed (e.g., via laser ablation or other biomechanical measurements)? Even a discussion of this limitation would be valuable.
Response - We were unable to do this experiment as the time and length scale over which the stretching and splitting of rosettes occurs made it difficult experimentally to combine with laser ablation. Furthermore, we have now shown in new graphs (Figure 2 D-F) how fusion and splitting events are consistently preceded by compression and stretching of the primordium, respectively. We believe this helps relate fusion and splitting events to expected changes in tension along the length of the primordium.

* It would also be informative to test whether dual perturbation (e.g., low-dose cxcl12 morpholino + PP1) could rescue the splitting phenotype by restoring balanced tension. This would help support the tension-based model.
Response - Figure 2C now includes such an experiment where PP1-dependent slowing of trailing cells is associated with splitting of a leading constriction, and subsequent heat shock induced cxcl12a, slows leading cells, which is associated with reconstitution of the leading constriction.

2. Coupling between atoh1a expression and apical constriction (Figure 2):
* The authors report that atoh1a-positive cells can exceed the number of apical constrictions following cxcl12 overexpression, consistent with rosette fusion. Given that Notch signaling limits SHCP number via lateral inhibition, one would expect this initial excess to resolve over time. It would be informative to examine whether SHCP numbers normalize at later stages.

© 2026. Published by The Company of Biologists under the terms of the Creative Commons Attribution License (https://creativecommons.org/licenses/by/4.0/).

Response - As noted in the response to Reviewer1, examination of the pattern of *atoh1a* expression in a large numbers of primordia (≈ 100) revealed that the *atoh1a* pattern is more complex and dynamic than we had appreciated, as both the degree to which the pattern is diffuse or discrete in association with one or more specific cell varied too much in controls to make it a useful proxy for identifying discrete Fgf signaling centers that normally initiate the formation of epithelial rosettes. Based on this observation we have removed this data from the main results. The dynamic and variable atoh1a expression likely reflects the dynamic process of lateral inhibition which eventually results in restriction of *atoh1a* to a single central cell in the maturing protoneuromast. Recognizing the dynamics of the normal pattern, we have nevertheless included an illustrative example of a control primordium, where individual atoh1a expressing cells are associated with individual constrictions, and an experimental primordium, where multiple Fgf signaling centers have potentially fused to form a constriction in association with a relatively large number of atoh1a cells, in the Supplementary (Figure S5).

* cxcr4b mutants exhibit impaired migration and likely undergo rosette fusion. Quantifying atoh1a-positive cells per rosette in these mutants could provide additional support for the model.
Response - Given the complexity of atoh1a expression discussed above, we decided not to pursue this suggestion.

* Similarly, does the sdf1 morpholino induce a comparable excess in atoh1a-positive cells, consistent with fusion events?
Response - These kinds of experiments will be pursued in the future once we have developed appropriate live reporters of Atoh1 expression or FGF activity

* The authors state that apical constrictions are not always associated with a single, well-defined atoh1a-expressing cell. However, clustered expression of atoh1a in newly forming rosettes has been described previously (e.g., Lecaudey et al., 2008; Nechiporuk et al., 2008; Matsuda et al., 2010). The current phrasing may mislead readers into thinking this is a novel observation. The authors should clarify this and appropriately cite prior literature.
Response - See above discussion of atoh1 which has been removed from the results section

3. Inhibition of Src signaling (PP1 treatment):
* This section is brief and complement the initial results on cxcl12a perturbation. Consider integrating it into the first section for clarity and flow.
Response - There are now two sections: one discussing cxcl12a over-expression and knock-down , both of which slow leading cells and promote fusion of constriction, and another, discussing PP1 or FGF over-expression, both of which transiently slow trailing cells and result in splitting of constrictions.

* The authors should examine atoh1a expression in PP1-treated embryos: does a new atoh1a-expressing cell appear in the split rosettes? This would clarify whether rosette splitting leads to re-specification of SHCPs.
Response – see responses about atoh1a above.

* See additional comments above regarding reproducibility and possible rescue experiments.

4. Modeling approaches and interpretation (Figures 3 & 5):
* The authors describe the Wnt-active domain as encompassing the leading 60% of the primordium. This is inconsistent with previous studies (e.g., Aman & Piotrowski, 2008; studies from the Chitnis lab), which place Wnt activity in the leading third and FGF signaling in the trailing two-thirds. This discrepancy should be acknowledged and justified.
Response - Nogare and Chitnis (2017) have used a Wnt reporter line to show the Wnt-active domain is typically associated with the leading 60%. This is the basis for associating the Wnt-active domain in the models with the leading 60%. It is important to recognize that these descriptions are caricatures and only as much detail has been incorporated in the Netlogo and CPM models to illustrate how mechanics can influence the pattern of clustering. For illustrative purposes, two compartments with potentially different speeds and with different spring strength were specified in the model primordia to illustrate how the balance of mechanical interactions that pull cells (Netlogo) together, coupled with tension associated with differential migration of the two compartments could determine and influence cell clustering. The model allows you to change the

© 2026. Published by The Company of Biologists under the terms of the Creative Commons Attribution License (https://creativecommons.org/licenses/by/4.0/).

relative proportion of these domains and observe any effect if desired.

In reality at least three different domains with distinct behaviors and expression patterns can be identified in the primordium. The primordium can be roughly divided into three domains, a leading third that typically has only Wnt and no rosettes, a middle third that has an overlap of Wnt and Fgf and is where new rosettes form, and a trailing third that typically has no Wnt and has only Fgf and is where protoneuromasts mature. However, much of this complexity is not incorporated in our “toy” models.

* The model shown in Figure 3 simulates cell aggregation rather than apical constriction. As the authors acknowledge, proximity does not necessarily correlate with rosette organization (as evidenced by nuclear positioning). The implications of this model should be clarified: what does it capture, and what are its limitations?
Response - We addressed some of these issues when discussing the limitations of the Netlogo models and the motivations for development of Cellular Potts models but have now included a longer discussion as noted below.
The Netlogo models represent complex apicobasally polarized cells as simple points in a 2D universe connected to their neighbors with spring-like links. The simplicity of these models makes it easier to visualize how the mechanical interactions that pull cells together, coupled with tension that pull cells apart, can result in self-organized clustering. In the context of this simple 2D representation, clustering of *turtles* correlates with rosette formation. The development of the sideways representation of the primordium in the Cellular Potts Model, helps extend the ideas developed with this initial simple representation with Netlogo *turtles* and *links* , where cell shape, polarization, and distinctions between apical constriction and cell adhesion could not be represented. Nevertheless, the simple Netlogo model helps develop a conceptual framework for understanding how a balance of interactions that pull apical compartments together, coupled with tension along the length of the primordium associated with migration could influence the pattern of cell clustering to form “rosettes”. By representing FGF cells as tripartite composites with basal, lateral, apical compartments, the CPM model was better able to explore emergent organization because of interactions between these distinct compartments in adjacent cells. It was possible to visualize how for a set of selected initial conditions, a relatively stable pattern of rosettes emerges, how slowing of leading or trailing domains results, respectively, in fusion or splitting of rosettes, and how increasing adhesive interactions between adjacent lateral compartments of Fgf cells can promote their reorganization to form a large trailing cluster. While these simulations recapitulate many of the experimental observations, these 2D representations remain caricatures and the primary strength of these models is conceptual. Much of the complexity of the cell biology associated with apical constriction, rosette formation, collective migration is not incorporated in these models. Furthermore, while *turtles* associated with spring-like *links* in Netlogo, and energy minimization associated with target parameters in Cellular Potts models, simulates emergent organization and collective behavior of primordium cells, these models are not based on a literal representation of measured real world physical interactions, space or time. Rather these models illustrate the power of Agent Based models, which can be used to predict emergent organization based on rules that define how the agents interact and behave at different scales.

* In the Cellular Potts Model (Figure 5), it would be informative to simulate slower migration of trailing cells (to mimic PP1 treatment) and examine whether this leads to rosette splitting. This would strengthen the link between experimental observations and model behavior.
Response - We have now added the suggested simulation to Fig. 5E.

* The rationale for associating increased contractility and adhesion in the model with Notch overactivation is unclear. The authors should elaborate on why this parameterization reflects Notch gain-of-function and how it relates to in vivo data.
Response - Previous studies had shown that loss of Notch signaling results in poor cohesion of neuromasts and a primordium that eventually falls apart (Matsuda et al 2009, Kozlovskaja-Gumbriene et al 2017) Data from Kozlovskaja-Gumbriene et al. (2017) demonstrated that Notch promotes expression of several cell adhesion molecules like EpCAM and E-Cadherin, as well as a number of junctional proteins. They also showed that broad exaggerated Notch activity results in reorganization of primordium cells so that they form one or two very large trailing rosettes. They hypothesized that this reorganization could result from the upregulation of such adhesion molecules. Our model illustrates how increasing adhesive interactions could indeed result in such

© 2026. Published by The Company of Biologists under the terms of the Creative Commons Attribution License (https://creativecommons.org/licenses/by/4.0/).

reorganization. This role for Notch in regulating adhesion in the primordium was a unique observation, distinct from its previously recognized well known role in lateral inhibition and restriction of atoh1a expression.

Additional minor comments:

* Line 76-77: "Posterior Lateral Line" should be in lowercase.
Response - They have been changed wherever they appeared.

* Line 78: "follow" should be "flow".
Response - Corrected in the revised version.

* Line 854: "μm" should be written as "μM".
Response - Corrected in the revised version.

* Line 904: "apical (pink)" should be "apical (red)".
Response - Corrected in the revised version.

Conclusion:
This manuscript presents evidence that mechanical forces influence the spatial organization of epithelial rosettes in the migrating lateral line primordium. The experimental framework and modeling are valuable, but key aspects require further quantification, clarification, and, where feasible, additional validation. With revision, this work has the potential to make a significant contribution to our understanding of how signaling and mechanics interact during collective epithelial morphogenesis.

Reviewer 3:

SUMMARY OF THE ADVANCE MADE IN THIS PAPER AND ITS POTENTIAL SIGNIFICANCE TO THE FIELD

Mukherjee et al describe experiments to test a model where interactions between migration and cellular interactions combine to form rosettes in the migrating lateral line primordium. They demonstrate how manipulation of migration rates of leading and trailing regions of the moving primordium result in changes in apical constrictions and altered rosette patterning. Assessing gene expression demonstrates a decoupling of rosette formation and atoh1a expression. Using agent-based models, the authors demonstrate how changes in migration and cellular interactions is sufficient to predict observed patterning differences. Using Cellular Potts models they model how apical constriction and migration contribute emergent rosette formation as migration proceeds. Overall the combination of embryonic manipulation and computational modeling demonstrate how mechanical coupling combined with changes in migration can influence dynamic patterning. Accompanying movies contribute to provide the overall picture.

Response- We thank the reviewer for their comments

SUGGESTIONS TO AUTHORS:

The authors manipulate cxcl12a by heat shock overexpression or morpholino knockdown to slow leading cells compared to trailing cells. The authors also describe that treatment with PP1 results in slowing trailing cells compared to leading cells. These manipulations in turn affect the number of rosettes. While the examples in Figure 1 and movies are clear, it would be good to have a quantitative assessment to describe the variation in rates of movement and numbers of rosettes. Does changes in migration precede changes in rosette number?
Response - We have now provided graphs that show how compression or stretching of the primordium precedes observed fusion or splitting of constrictions.

In figure 2, it would be nice if labels were included for the color-coded images to make it easier to understand.
Response - Figure 2 in the original manuscript has now been removed from the revised manuscript. We found the atoh1a expression pattern to be complex and variable, even in controls. Hence, we

© 2026. Published by The Company of Biologists under the terms of the Creative Commons Attribution License (https://creativecommons.org/licenses/by/4.0/).

decided to remove the original Figure 2. A detailed description of the complexities and limitations of using atoh1a expression as proxy for FGF patterning has been included in the Discussion section.

In figure 5, I found it difficult to make out the individual cells - I wonder whether a close-up of a group of cells could be included as an inset perhaps in the black space of each panel.
Response – Insets have been provided

Second decision letter

MS ID#: dev.204973R1

MS TITLE: Mechanical interactions dynamically influence rosette morphogenesis in the migrating zebrafish Posterior Lateral Line primordium

AUTHORS: Abhishek Mukherjee; Michael Hilzendeger; Arin Rinvelt; Sana Fatma; Megan Schupp; Damian Dalle Nogare; Ajay Balwant Chitnis

Dear Ajay,

You will be pleased to see that the reviewers are happy with your revisions and there are only a couple of very minor suggestions to address before proceeding to publication.

Reviewer 1

The authors have performed additional quantifications and have extended the Materials and methods, addressing the rationale for parameter choice in the model. Overall, the revised manuscript has been substantially improved, and my concerns have been appropriately addressed.

Reviewer 2

I thank the authors for addressing most of my comments. The added experiments and information on sample size contribute add support to the main conclusions and I consider the manuscript suitable for publication.

Minor comments:

I) the first paragraph of the discussion (lines 428 to 453) is very redundant with part of the introduction with some sentences being almost identical. For example:

Introduction: "The local activation of Wnt signaling, coupled with its long-range inhibition by FGF signaling, constitutes a patterning system that has the potential to initiate FGF signaling in a center-biased pattern (Dalle Nogare and Chitnis, 2020). This center-biased FGF signaling center initiates formation of a nascent protoneuromast by coordinating both the specification of a central atoh1a-expressing SHCP and reorganization of its surrounding cells to form an epithelial rosette"

Discussion: "local self-activation of Wnt signaling, coupled with long range inhibition of Wnt activity by FGF-dependent Dkk1b expression, has the potential to determine self-organization of center-biased FGF signaling centers in the wake of a shrinking Wnt activity domain (Dalle Nogare and Chitnis, 2020). These signaling centers create a prepattern of FGF signaling that initiates formation of protoneuromasts with their central atoh1a-expressing cell and surrounding cells organized to form epithelial rosettes".

This part of the discussion should be revised and reduced to avoid repetition of information given in the introduction

© 2026. Published by The Company of Biologists under the terms of the Creative Commons Attribution License (https://creativecommons.org/licenses/by/4.0/).

ii) The abbreviation SHCP (line 108) is not explained

iii) "it became apparent that such fusion and splitting events were also sometimes be observed in control PLLps" (line 222-223): this sentence should be revised.

Reviewer 3

SUMMARY OF THE ADVANCE MADE IN THIS PAPER AND ITS POTENTIAL SIGNIFICANCE TO THE FIELD

The authors have addressed my previous concerns.

SUGGESTIONS TO AUTHORS

Second revision

Author response to reviewers' comments

Dear Steve,

We are pleased that revisions to our paper "Signaling and Mechanics influence the number and size of epithelial rosettes in the migrating zebrafish Posterior Lateral Line primordium" now make it suitable for publication with minor changes. We have made the following additional changes based on the recommendation of the reviewers.

1. The discussion has been edited to eliminate a section that repeated a description provided in the introduction.
2. The acronym SHCP used inadvertently has been expanded to Sensory Hair Cell Progenitor.
3. Additional text, as noted in the attached manuscript with highlighted changes, has been edited to reduce the number of words.

We look forward to final acceptance of the paper and seeing the manuscript in Press.

Third decision letter

MS ID#: dev.204973R2

MS TITLE: Mechanical interactions dynamically influence rosette morphogenesis in the migrating zebrafish Posterior Lateral Line primordium

AUTHORS: Abhishek Mukherjee; Michael Hilzendeger; Arin Rinvelt; Sana Fatma; Megan Schupp; Damian Dalle Nogare; Ajay Balwant Chitnis

Dear Ajay,

I am happy to tell you that your manuscript has been accepted for publication in Development, pending our standard publication integrity checks.

© 2026. Published by The Company of Biologists under the terms of the Creative Commons Attribution License (https://creativecommons.org/licenses/by/4.0/).
